# Supplementary figures and images for: Morphological and phylogenetic appraisal of Ophioceras (Ophioceraceae, Magnaporthales)
Source: PLoS One. 2021 Aug 25;16(8):e0253853. doi: 10.1371/journal.pone.0253853 (PMC8386851; doi:10.1371/journal.pone.0253853)

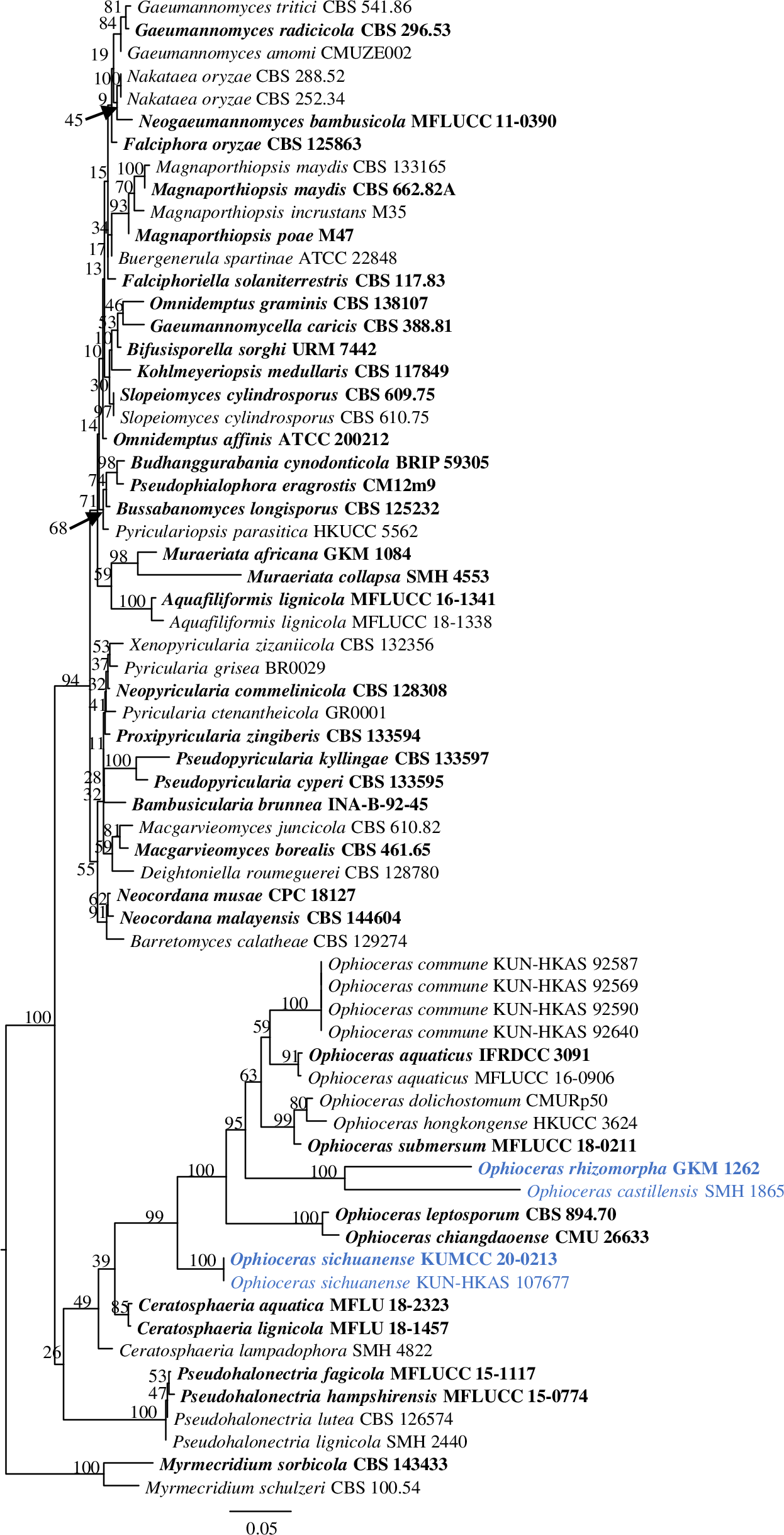

Supplement: S1 Fig — (TIF) [file pone.0253853.s001.tif]

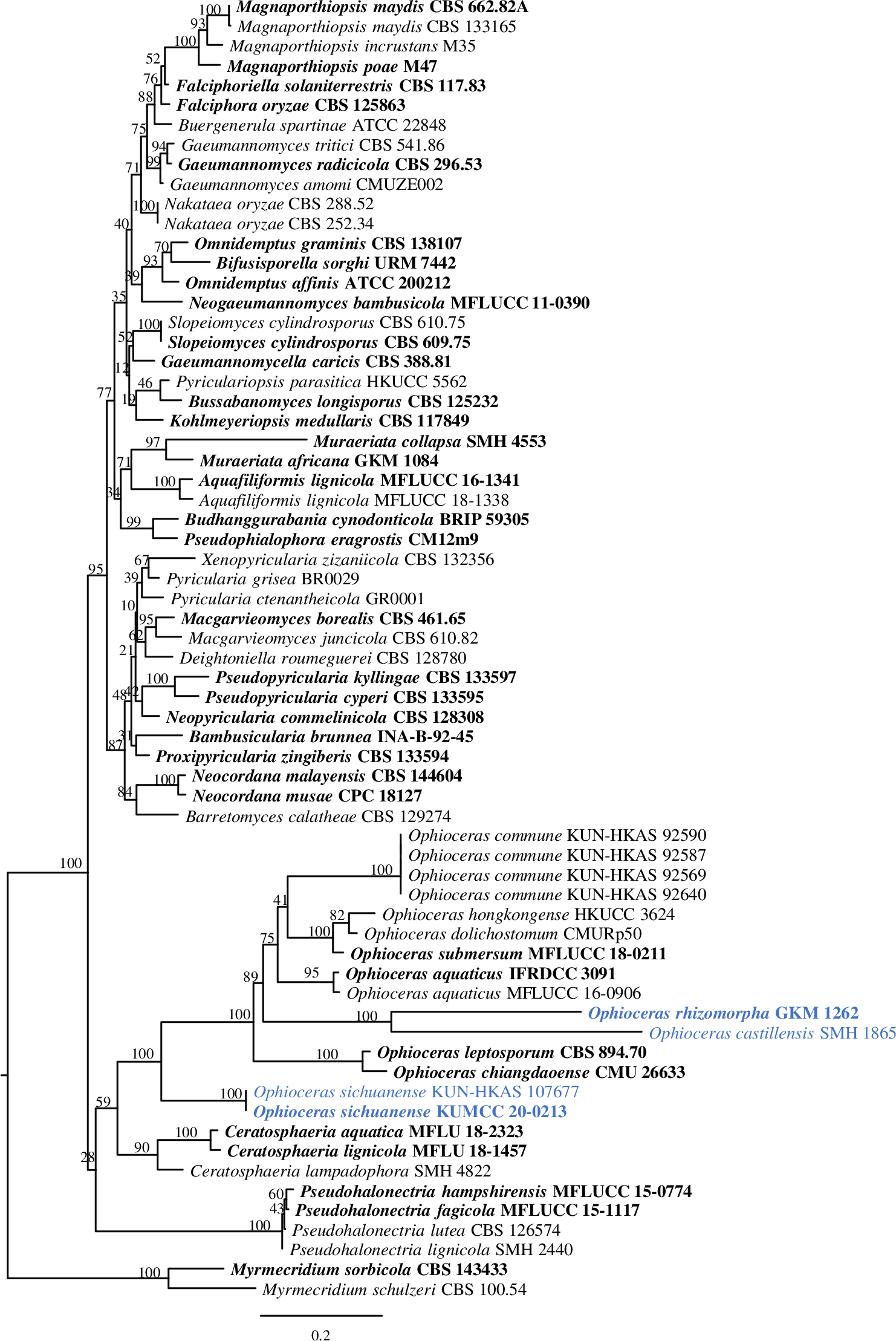

Supplement: S2 Fig — (TIF) [file pone.0253853.s002.tif]
