# Supplementary material for: Morphological and phylogenetic appraisal of Ophioceras (Ophioceraceae, Magnaporthales)
Source: PLoS One. 2021 Aug 25;16(8):e0253853. doi: 10.1371/journal.pone.0253853 (PMC8386851; doi:10.1371/journal.pone.0253853)
Supplement: Attachment — Submitted filename: PONE-D-20-37408_Reviewer_2.pdf [file pone.0253853.s005.pdf]

PLOS ONE

Morphological and phylogenetic appraisal of Ophioceras (Ophioceraceae, Magnaporthales)

--Manuscript Draft--

**Manuscript Number:**	PONE-D-20-37408R1
**Article Type:**	Research Article
**Full Title:**	Morphological and phylogenetic appraisal of Ophioceras (Ophioceraceae, Magnaporthales)
**Short Title:**	Ophioceras (Ophioceraceae, Magnaporthales)
**Corresponding Author:**	Rungtiwa Phookamsak<br>Kunming Institute of Botany Chinese Academy of Sciences<br>Kunming, Yunnan, China CHINA
**Keywords:**	1 novel taxon, 2 new combinations, bambusicolous fungi, Ceratosphaerella, Sordariomycetes, taxonomy
**Abstract:**	Ophioceras is accommodated in the monotypic family Ophioceraceae (Magnaporthales, Sordariomycetes), and the genus is delimited based on molecular data. During an ongoing survey of bambusicolous fungi in southwest China and Thailand, we collected a submerged decaying branch of bamboo from Sichuan Province, China and an Ophioceras species occurring on this substrate was observed and isolated. An Ophioceras taxon was delimited based on morphological characteristics and combined LSU, RPB1 and ITS sequence analyses and is described as Ophioceras sichuanense sp. nov. in this paper. The species formed a well-supported clade basal to Ophioceras (100% ML, 1.00 PP). Based on the updated phylogenetic tree of Magnaporthales, Ceratosphaerella castillensis (generic type) and C. rhizomorpha formed a clade within Ophioceras and morphologically resemble Ophioceras . Therefore, Ceratosphaerella is synonymized under Ophioceras . The phylogenetic relationships of Ophioceras are discussed in relation to morphological similarities of genera in Magnaporthales. The generic circumscription of Ophioceras is emended.
**Order of Authors:**	Hong-Bo Jiang
	Kevin D. Hyde
	Er-Fu Yang
	Pattana Kakumyan
	Ali H. Bahkali
	Abdallah M. Elgorban
	Samantha C. Karunarathna
	Rungtiwa Phookamsak
	Saisamorn Lumyong
**Response to Reviewers:**	We have revised our manuscript based on the reviewer's comments and hope that the updated version is satisfied to published in Plos One.
**Additional Information:**	
**Question**	**Response**
**Financial Disclosure**<br><br>Enter a financial disclosure statement that describes the sources of funding for the work included in this submission. Review the submission guidelines for detailed	The authors are grateful for the Mushroom Research Foundation, Chiang Rai, Thailand and the Key Research Program of Frontier Sciences of the Chinese Academy of Sciences (grant no. QYZDY-SSW-SMC014) for supporting this research. Kevin D. Hyde thanks the Foreign Experts Bureau of Yunnan Province, Foreign Talents Program (2018; grant no. YNZ2018002), Impact of Climate Change on Fungal Diversity and Biogeography in the Greater Mekong Subregion (grant no:

Powered by Editorial Manager® and ProduXion Manager® from Aries Systems Corporation

requirements. View published research articles from *PLOS ONE* for specific examples.<br><br>This statement is required for submission and **will appear in the published article** if the submission is accepted. Please make sure it is accurate.<br><br>**Unfunded studies**<br>Enter: *The author(s) received no specific funding for this work.*<br><br>**Funded studies**<br>Enter a statement with the following details:<br>• Initials of the authors who received each award<br>• Grant numbers awarded to each author<br>• The full name of each funder<br>• URL of each funder website<br>• Did the sponsors or funders play any role in the study design, data collection and analysis, decision to publish, or preparation of the manuscript?<br>• **NO** - Include this sentence at the end of your statement: *The funders had no role in study design, data collection and analysis, decision to publish, or preparation of the manuscript.*<br>• **YES** - Specify the role(s) played.<br><br>* typeset	RDG6130001). Rungtiwa Phookamsak sincerely acknowledges the CAS President's International Fellowship Initiative (PIFI) for young staff (grant no. Y9215811Q1), the National Science Foundation of China (NSFC) project code 31850410489 (grant no. Y81I982211) and Chiang Mai University for financial support. Samantha C. Karunarathna thanks the CAS President's International Fellowship Initiative (PIFI) young staff under the grant number: 2020FYC0002 and the National Science Foundation of China (NSFC) under the project code 31851110759 for funding. Ali H. Bahkali and Abdallah M. Elgorban thanks the International Scientific Partnership Program ISPP at King Saud University through ISPP#0089. The funders had no role in study design, data collection and analysis, decision to publish, or preparation of the manuscript.
**Competing Interests**<br><br>Use the instructions below to enter a competing interest statement for this submission. On behalf of all authors, disclose any competing interests that could be perceived to bias this work—acknowledging all financial support and any other relevant financial or non-financial competing interests.<br><br>This statement **will appear in the published article** if the submission is accepted. Please make sure it is accurate. View published research articles from *PLOS ONE* for specific examples.	The authors have declared that no competing interests exist.

Powered by Editorial Manager® and ProduXion Manager® from Aries Systems Corporation

**NO authors have competing interests**<br><br>Enter: *The authors have declared that no competing interests exist.*<br><br>**Authors with competing interests**<br><br>Enter competing interest details beginning with this statement:<br><br>*I have read the journal's policy and the authors of this manuscript have the following competing interests: [insert competing interests here]*<br><br>* typeset	
**Ethics Statement**<br><br>Enter an ethics statement for this submission. This statement is required if the study involved:<br><br>• Human participants<br>• Human specimens or tissue<br>• Vertebrate animals or cephalopods<br>• Vertebrate embryos or tissues<br>• Field research<br><br>Write "N/A" if the submission does not require an ethics statement.<br><br>General guidance is provided below. Consult the submission guidelines for detailed instructions. **Make sure that all information entered here is included in the Methods section of the manuscript.**	N/A

**Format for specific study types**<br><br>**Human Subject Research (involving human participants and/or tissue)**<br>• Give the name of the institutional review board or ethics committee that approved the study<br>• Include the approval number and/or a statement indicating approval of this research<br>• Indicate the form of consent obtained (written/oral) or the reason that consent was not obtained (e.g. the data were analyzed anonymously)<br><br>**Animal Research (involving vertebrate animals, embryos or tissues)**<br>• Provide the name of the Institutional Animal Care and Use Committee (IACUC) or other relevant ethics board that reviewed the study protocol, and indicate whether they approved this research or granted a formal waiver of ethical approval<br>• Include an approval number if one was obtained<br>• If the study involved *non-human primates*, add *additional details* about animal welfare and steps taken to ameliorate suffering<br>• If anesthesia, euthanasia, or any kind of animal sacrifice is part of the study, include briefly which substances and/or methods were applied<br><br>**Field Research**<br><br>Include the following details if this study involves the collection of plant, animal, or other materials from a natural setting:<br>• Field permit number<br>• Name of the institution or relevant body that granted permission	
**Data Availability**<br><br>Authors are required to make all data underlying the findings described fully available, without restriction, and from the time of publication. PLOS allows rare exceptions to address legal and ethical concerns. See the PLOS Data Policy and FAQ for detailed information.	Yes - all data are fully available without restriction

A Data Availability Statement describing where the data can be found is required at submission. Your answers to this question constitute the Data Availability Statement and **will be published in the article**, if accepted.<br><br>**Important:** Stating 'data available on request from the author' is not sufficient. If your data are only available upon request, select 'No' for the first question and explain your exceptional situation in the text box.<br><br>Do the authors confirm that all data underlying the findings described in their manuscript are fully available without restriction?	
**Describe where the data may be found in full sentences. If you are copying our sample text, replace any instances of XXX with the appropriate details.**<br><br>• If the data are **held or will be held in a public repository**, include URLs, accession numbers or DOIs. If this information will only be available after acceptance, indicate this by ticking the box below. For example: *All XXX files are available from the XXX database (accession number(s) XXX, XXX.*).<br>• If the data are all contained **within the manuscript and/or Supporting Information files**, enter the following: *All relevant data are within the manuscript and its Supporting Information files.*<br>• If neither of these applies but you are able to provide **details of access elsewhere**, with or without limitations, please do so. For example:<br><br>*Data cannot be shared publicly because of [XXX]. Data are available from the XXX Institutional Data Access / Ethics Committee (contact via XXX) for researchers who meet the criteria for access to confidential data.*<br><br>*The data underlying the results presented in the study are available from (include the name of the third party*	All relevant data are within the manuscript and its Supporting Information files.

and contact information or URL).<br>• This text is appropriate if the data are owned by a third party and authors do not have permission to share the data.<br><br>* typeset	
Additional data availability information:	Tick here if the URLs/accession numbers/DOIs will be available only after acceptance of the manuscript for publication so that we can ensure their inclusion before publication.

Powered by Editorial Manager® and ProduXion Manager® from Aries Systems Corporation

Manuscript Click here to access/download;Manuscript;Manuscript.docx

Morphological and phylogenetic appraisal of *Ophioceras* (Ophioceraceae, Magnaporthales)

Hong-Bo Jiang[1,3,4,5,7], Kevin D. Hyde[1,3,4,8], Er-Fu Yang[3,7,9], Pattana Kakumyan[5], Ali H. Bahkali[8], Abdallah M. Elgorban[8], Samantha C. Karunarathna[3,6,7], Rungtiwa Phookamsak[1,3,6,7]*, Saisamorn Lumyong[1,2]*

[1] Research Center of Microbial Diversity and Sustainable Utilization, Faculty of Sciences, Chiang Mai University, Chiang Mai 50200, Thailand

[2] Academy of Science, The Royal Society of Thailand, Bangkok 10300, Thailand

[3] CAS Key Laboratory for Plant Diversity and Biogeography of East Asia, Kunming Institute of Botany, Chinese Academy of Sciences, Kunming 650201, Yunnan, P.R. China

[4] Center of Excellence in Fungal Research, Mae Fah Luang University, Chiang Rai 57100, Thailand

[5] School of Science, Mae Fah Luang University, Chiang Rai 57100, Thailand

[6] CIFOR-ICRAF China Program, World Agroforestry (ICRAF), Kunming 650201, Yunnan, China

[7] Honghe Center for Mountain Futures, Kunming Institute of Botany, Chinese Academy of Sciences, Honghe County 654400, Yunnan, P.R. China

[8] Department of Botany and Microbiology, College of Science, King Saud University, P.O. Box: 2455, Riyadh, 1145, Saudi Arabia

[9] Department of Biology, Faculty of Science, Chiang Mai University, Chiang Mai 50200, Thailand

*Corresponding Author:
scboi009@gmail.com (SL)
jomjam.rp2@gmail.com (RP)

Abstract

Ophioceras is accommodated in the monotypic family Ophioceraceae (Magnaporthales, Sordariomycetes), and the genus is delimited based on molecular data. During an ongoing survey of bambusicolous fungi in southwest China and Thailand, we collected a submerged decaying branch of bamboo from Sichuan Province, China and an *Ophioceras* species occurring on this substrate was observed and isolated. An *Ophioceras* taxon was delimited based on morphological characteristics and combined LSU, RPB1 and ITS sequence analyses and is described as *Ophioceras sichuanense* sp. nov. ~~in this paper~~. The species formed a well-supported clade basal to *Ophioceras* (100% ML, 1.00 PP). Based on the updated phylogenetic tree of Magnaporthales, *Ceratosphaerella castillensis* (generic type) and *C. rhizomorpha* formed a clade within *Ophioceras* and morphologically resemble *Ophioceras*. Therefore, *Ceratosphaerella* is synonymized under *Ophioceras*. The phylogenetic relationships of *Ophioceras* are discussed in relation to morphological similarities of genera in Magnaporthales. The generic circumscription of *Ophioceras* is emended.

Keywords – 1 novel taxon, 2 new combinations, bambusicolous fungi, *Ceratosphaerella*, Sordariomycetes, taxonomy

Introduction

Klaubauf et al. [1] introduced the family Ophioceraceae to accommodate *Ophioceras* Sacc. which is typified by *O. dolichostomum* (Berk. & M.A. Curtis) Sacc. The family is recognized as black, immersed to superficial, globose to subglobose, perithecial ascomata with long, periphysate necks, 8-spored, unitunicate, subcylindrical to narrowly fusoid asci with a J-, apical ring, and filiform, hyaline to olivaceous, septate ascospores without sheaths [1,2,3]. Species of Ophioceraceae are saprobes on wood and herbaceous plants in aquatic or terrestrial habitats [1,2,3,4,5,6].

Saccardo [7] introduced *Ophioceras* to accommodate taxa with immersed, sub carbonaceous, globose perithecia, with conical-cylindrical, filiform ostioles, elongate asci and filiform, septate ascospores. The genus initially accommodated *O. bacillatum* (Cooke) Sacc. *O. dolichostomum*, *O. friesii* (Mont.) Sacc., *O. hystrix* (Ces.) Sacc., *O. macrocarpum* (Sacc.) Sacc., *O. longisporum* Sacc. and *O. therryanum* (Sacc. & Roum.) Sacc [7]. *Ophioceras* was accommodated in Magnaporthaceae based on limited molecular data [8,9,10]. However, the genus was excluded from Magnaporthaceae and accommodated in the separate family Ophioceraceae in Magnaporthales based on combined LSU and RPB1 phylogenetic analyses [1]. To date, the asexual morph of *Ophioceras* has not yet been reported [1,2,6], and only 40 epithets are listed under this genus in Index Fungorum [11]. However, only eleven *Ophioceras* species have molecular data in GenBank, and only LSU and ITS sequence data are most available for these species [13].

Ophioceras species are commonly discovered from wood in freshwater and are generally clumped under the name *Ophioceras* sp. [40,41], *O. commune* [44,45,46] or *O. dolichostomum* [51,52,53,54]. Twenty-three *Ophioceras* species are listed in Species Fungorum [55] and accepted in Hyde et al. [3]. *Ophioceras* species occur in disparate streams in different islands and continents and are therefore likely to have separated for millions of years ago, potentially explaining their

evolution into distinct species. The number of existing *Ophioceras* species is therefore likely to exceed more than presently known. Hyde et al. [56] has shown that numerous new taxa await description across most under-collected and -studied.

Huhndorf et al. [8] introduced *Ceratosphaerella castillensis* (≡ *Ceratosphaeria castillensis*) when the new genus *Ceratosphaerella* was introduced, and this species was designated as the generic type. Huhndorf et al. [8] examined the isotype of *Ceratosphaeria castillensis* and compared it with the morphology of their collection (SMH1865) and confirmed the conspecific status based only on morphological characteristics. Molecular data of *C. castillensis* was obtained from Huhndorf's collection (SMH1865), but the type specimen of *C. castillensis* has not yet been sequenced.

Ceratosphaerella comprises *C. castillensis* (C.L. Sm.) Huhndorf, Greif, Mugambi & A.N. Mill. and *C. rhizomorpha* Huhndorf & Mugambi, introduced by Huhndorf et al. [8]. Phylogenetic analyses of the LSU sequence dataset showed that *Ceratosphaerella* grouped with *Ophioceras* in Magnaporthaceae [8]. Klaubauf et al. [1] only accommodated *Ophioceras* into Ophioceraceae, but they did not incorporate *Ceratosphaerella* in their analyses. Thus, *Ceratosphaerella* remains in Magnaporthaceae. To date, there are only two species included in *Ceratosphaerella* [11], and these taxa only have LSU sequence data in GenBank [13]. *Ceratosphaerella castillensis* has been reported only as a sexual morph, while *C. rhizomorpha* is holomorphic and has a Didymobotryum-like asexual morph [8]. In Luo et al. [2], *Ceratosphaerella* did not group within Magnaporthaceae but clustered with *Ophioceras* in Ophioceraceae. However, Luo et al. [2] did not verify the phylogenetic status of *Ceratosphaerella* in Ophioceraceae, leading to the uncertain placement of the genus.

This study aims to introduce the novel taxon, *Ophioceras sichuanense* sp. nov., as a saprobe on a submerged bamboo branch in Sichuan Province, China based on morphological characteristics and multi-locus phylogeny while also resolving the congeneric status of *Ceratosphaerella* and *Ophioceras* in Ophioceraceae based on a morpho-molecular approach. *Ceratosphaerella* is synonymized under *Ophioceras*, and two species combinations are introduced, including an updated generic description, illustration, and multi-locus phylogeny.

Materials and methods

Sample collection, observation and isolation

Decaying branches of bamboo submerged in freshwater were collected in the stream in the Shunan Artificial Bamboo Forest, Sichuan Province, P.R. China in July 2019. Samples were kept in a paper bag for further morphological examination in the laboratory. Pseudostromata visualized on decaying branches of bamboo were observed and examined under a stereo microscope (Motic series SMZ 140) and captured via digital phone camera (iPhone 7, Apple Inc., USA). Microscopic features (e.g., asci, ascospores and paraphyses) were prepared using the squashing mount technique in sterilized distilled water on clean slides for morphological study. Sections of pseudostromatic ascomata, ostiolar necks and peridial structures were by free-hand sectioning using Gillette razor blades. Melzer's reagent and Indian ink were used to detect the J-/J+ apical ring of the asci and mucilaginous sheath surrounding the ascospores, respectively. Morphological features visualized under a Nikon ECLIPSE Ni compound microscope were photographed using a Canon EOS 600D digital camera. Measurements (n = 10–20) of pseudostromata, locules, peridium, paraphyses, asci and ascospores were carried out using Tarosoft (R) Image Frame Work

version 0.9.7. Photographic plate and line drawings of fungal morphologies were edited and combined using Adobe Photoshop CS6 (Adobe Systems Inc., USA).

Single spore isolation based on the spore suspension technique [15] was carried out to obtain a pure fungal culture. Germinated ascospores were transferred to the new potato dextrose agar plates (PDA; Qingdao Daily Water Biotechnology co. LTD. Shandong, P.R. China) under aseptic conditions and grown under normal day/nightlight at room temperature. Culture characteristics (e.g., growth, shape, colour, margin, elevation, consistency) were checked and recorded after one week and four weeks.

The holotypic specimen is conserved in the herbarium of Cryptogams Kunming Institute of Botany Academia Sinica (KUN-HKAS), Yunnan, P.R. China. The isotype is stored in the herbarium of Mae Fah Luang University, Chiang Rai, Thailand (MFLU). Ex-type living cultures are preserved in the Kunming Institute of Botany Culture Collection (KUMCC) and Mae Fah Luang University Culture Collection (MFLUCC). Facesoffungi and Index Fungorum numbers were registered for the new taxon [11,16].

DNA extraction, amplification and sequencing

Fungal genomic DNA was extracted from fresh mycelia using the Biospin Fungus Genomic DNA Extraction Kit (BioFlux®, P.R. China) following manufacturer's instructions (Hangzhou, P.R. China) and also extracted from fruiting bodies (= pseudostromata) directly using the Forensic DNA Kit (Omega®, USA) for a duplicated strain. DNA amplification was performed by polymerase chain reaction (PCR). Four gene regions including the internal transcribed spacer (ITS), 28S large subunit rDNA (LSU), 18S small subunit rDNA (SSU) and translation elongation factor 1-alpha (TEF1-α) were used to amplify PCR fragments using forward and reward primer pairs: ITS5/ITS4 [17], LR0R/LR5 [18], NS1/NS4 [17], EF1-983F/EF1-2218R [19], respectively. PCR

reactions were conducted in a 25 μL total volume, consisted of 2 μl of DNA template, 1 μl of each forward and reverse primer, 12.5 μl of 2× Power Taq PCR Master Mix (Beijing BioTeke Corporation, P.R. China) and 8.5 μl double-distilled water (ddH$_2$O). The PCR thermal cycle program for ITS, LSU, SSU and TEF1-α gene was set up following Jiang et al. [20]. PCR fragments were purified and sequenced at TsingKe Biological Technology (Beijing) Co., Ltd, P.R. China.

Molecular phylogeny

The newly generated sequences (ITS, LSU and SSU) of fungal strains were initially subjected to the basic local alignment search tool (BLASTn) via the National Center for Biotechnology Information web portal (NCBI; https://blast.ncbi.nlm.nih.gov) for discovering closely related fungal taxa. In order to clarify the phylogenetic placement of the new isolate, the representative taxa in Magnaporthales were incorporated with the new taxon to perform the sequence data matrix for further analysis. These representative taxa of Magnaporthales were downloaded from the GenBank database (Table 1) based on recent publications [2,14].

Preliminary single-gene data matrixes were aligned via MAFFT v. 7.452 [21] and improved manually in BioEdit v. 5.0.6 [22]. The single-gene alignments of LSU and ITS data matrixes were prior analyzed by maximum-likelihood (ML) criterion using RAxML v. 7.0.3 [23, 24] for checking if there are any conflicts between the tree topologies. The concatenated LSU-ITS and LSU-RPB1-ITS sequence datasets were further analyzed based on maximum-likelihood (ML) and Bayesian inference (BI) criteria and the tree topologies of these combined gene datasets were compared for checking the congruence of the tree topologies. The concatenated LSU-ITS and LSU-RPB1-ITS sequence datasets comprise 64 strains of ingroup taxa in Magnaporthales. *Myrmecridium schulzeri*

(CBS 100.54) and *M. sorbicola* (CBS 143433) were selected as the outgroup taxa. It is notable that the SSU and TEF1-α loci are not included in the present analyses due to fewer available sequences of SSU and TEF1-α loci in our complied dataset, and thus, these two loci were excluded to avoid missing data or gaps which may affect tree topology.

Maximum-likelihood (ML) criterion was analyzed by the online tool RAxML-HPC v.8 on XSEDE (8.2.12) via CIPRES Science Gateway v. 3.3 web portal [25]. The ML + thorough bootstrap parameters were set at default values but modified as 1000 replications of bootstraps (-N 1000) and using the GTRGAMMA model.

The best-fit evolutionary models of nucleotide substitution for LSU, RPB1 and ITS loci were evaluated by MrModeltest 2.3 [26], and the GTR+I+G substitution model under the Akaike Information Criterion (AIC) was the best-fit evolutionary model for each locus. Bayesian inference (BI) analysis was performed by MrBayes v. 3.1.2 [27]. The Markov Chain Monte Carlo sampling (MCMC) sampling method was used to determine posterior probabilities (PP) [28,29]. One million generations of six simultaneous Markov chains were run and sampled every 100th generation. MCMC heated chain was set up with a “temperature” value at 0.15. The burn-in was set to 20% of all sampled trees, meaning that sampled trees beneath the asymptote (20%) were discarded. Posterior probabilities values were then calculated from the remaining 8000 trees in the majority rule consensus tree.

The final phylogram presented in this study was visualized in FigTree v. 1.4.0 (http://tree.bio.ed.ac.uk/software/figtree/). The phylogenetic tree was edited in Microsoft Office PowerPoint 2016 (Microsoft Inc., USA) and converted to jpeg file using Adobe Photoshop CS6 (Adobe Systems Inc., USA). New sequences generated from the present study were registered for

GenBank accession numbers (Table 1). The final alignment and phylogram are submitted in TreeBASE (https://www.treebase.org/, submission ID: 26989).

Table 1. Detailed information of fungal taxa used in the phylogenetic analyses. The newly generated sequences are indicated in **blue**, and the ex-type strains are in **bold**.

Species name	Culture collection/	GenBank accession numbers		
	Voucher no.	LSU	RPB1	ITS
Aquafiliformis lignicola	**MFLUCC 16-1341**	MK835815	/	MK828615
Aquafiliformis lignicola	MFLUCC 18-1338	MK835814	/	MK828614
Bambusicularia brunnea	**INA-B-92-45**	NG_058671	KM485043	NR_145387
Barretomyces calatheae	CBS 129274	MH876639	KM485045	MH865202
Bifusisporella sorghi	**URM 7442**	NG_067852	MK060159	NR_164042
Budhanggurabania cynodonticola	**BRIP 59305**	NG_058678	KP162143	NR_137952
Buergenerula spartinae	ATCC 22848	DQ341492	JX134720	JX134666
Bussabanomyces longisporus	**CBS 125232**	NG_058668	KM485046	NR_145385
Ceratosphaeria aquatica	**MFLU 18-2323**	MK835812	/	MK828612
Ceratosphaeria lampadophora	SMH 4822	AY346270	/	/
Ceratosphaeria lignicola	**MFLU 18-1457**	MK835813	/	MK828613
Deightoniella roumeguerei	CBS 128780	MH876533	KM485047	MH865092
Falciphora oryzae	**CBS 125863**	NG_064356	KJ026706	NR_153972
Falciphoriella solaniterrestris	**CBS 117.83**	NG_058108	KM485058	NR_153995
Gaeumannomycella caricis	**CBS 388.81**	NG_058109	KM485059	NR_146245
Gaeumannomyces amomi	CMUZE002	DQ341493	/	AY265318
Gaeumannomyces radicicola	**CBS 296.53**	NG_058089	KM009194	NR_146246
Gaeumannomyces tritici	CBS 541.86	DQ341497	/	/
Kohlmeyeriopsis medullaris	**CBS 117849**	NG_058110	KM485068	NR_154068
Macgarvieomyces borealis	**CBS 461.65**	NG_058088	KM485070	NR_145384

**Species name**	**Culture collection/ Voucher no.**	**GenBank accession numbers**		
		**LSU**	**RPB1**	**ITS**
Macgarvieomyces juncicola	CBS 610.82	KM484970	KM485071	KM484855
Magnaporthiopsis incrustans	M35	JF414892	JF710437	JF414843
Magnaporthiopsis maydis	CBS 133165	KX306614	/	KX306544
Magnaporthiopsis maydis	**CBS 662.82A**	NG_058111	KM485072	NR_154175
Magnaporthiopsis poae	**M47**	JF414885	JF710433	JF414836
Muraeriata africana	**GKM 1084**	EU527995	/	/
Muraeriata collapsa	**SMH 4553**	EU527996	/	/
Myrmecridium schulzeri	CBS 100.54	EU041826	/	EU041769
Myrmecridium sorbicola	**CBS 143433**	NG_063957	/	NR_158871
Nakataea oryzae	CBS 252.34	MH867001	KM485078	KM484862
Nakataea oryzae	CBS 288.52	MH868571	KM485080	MH857040
Neocordana malayensis	**CBS 144604**	NG_066327	/	NR_163364
Neocordana musae	**CPC 18127**	LN713290	/	NR_154265
Neogaeumannomyces bambusicola	**MFLUCC 11-0390**	NG_059556	/	NR_146247
Neopyricularia commelinicola	**CBS 128308**	NG_058112	KM485087	NR_154226
Omnidemptus affinis	**ATCC 200212**	NG_059478	JX134728	NR_154292
Omnidemptus graminis	**CBS 138107**	MK487734	/	NR_164058
Ophioceras aquaticus	**IFRDCC 3091**	JQ797433	/	JQ797440
Ophioceras aquaticus	MFLUCC 16-0906	MK835810	/	MK828611
Ophioceras castillensis (as *Ceratosphaeria castillensis*)	SMH 1865	EU527997	/	/
Ophioceras chiangdaoense	**CMU 26633**	NG_066356	/	/
Ophioceras commune	KUN-HKAS 92569	MH795820	/	MH795815
Ophioceras commune	KUN-HKAS 92587	MH795819	/	MH795814
Ophioceras commune	KUN-HKAS 92590	MK835809	/	MK828610
Ophioceras commune	KUN-HKAS 92640	MH795818	/	MH795813

Species name	Culture collection/ Voucher no.	GenBank accession numbers		
		LSU	RPB1	ITS
Ophioceras dolichostomum	CMURp50	DQ341504	/	/
Ophioceras hongkongense	HKUCC 3624	DQ341509	/	/
Ophioceras leptosporum	**CBS 894.70**	NG_057959	JX134732	NR_111768
Ophioceras rhizomorpha (as *Ceratosphaerella rhizomorpha*)	**GKM 1262**	EU527998	/	/
Ophioceras sichuanense	KUN-HKAS 107677	MW057782	/	MW057779
Ophioceras sichuanense	**KUMCC 20-0213**	MT995046	/	MT995045
Ophioceras submersum	**MFLUCC 18-0211**	MK835811	/	/
Proxipyricularia zingiberis	**CBS 133594**	NG_063934	KM485091	AB274434
Pseudohalonectria fagicola	**MFLUCC 15-1117**	KX426219	/	/
Pseudohalonectria hampshirensis	**MFLUCC 15-0774**	KX426218	/	/
Pseudohalonectria lignicola	SMH 2440	AY346299	/	/
Pseudohalonectria lutea	CBS 126574	MH875622	/	MH864160
Pseudophialophora eragrostis	**CM12m9**	KF689638	KF689618	NR_146240
Pseudopyricularia cyperi	**CBS 133595**	NG_058113	/	NR_137920
Pseudopyricularia kyllingae	**CBS 133597**	NG_058114	KM485096	NR_155645
Pyricularia ctenantheicola	GR0001	KM484994	KM485098	KM484878
Pyricularia grisea	BR0029	KM484995	KM485100	KM484880
Pyriculariopsis parasitica	HKUCC 5562	DQ341514	/	/
Slopeiomyces cylindrosporus	**CBS 609.75**	KM485040	KM485158	KM484944
Slopeiomyces cylindrosporus	CBS 610.75	NG_057751	JX134721	NR_120170
Xenopyricularia zizaniicola	CBS 132356	KM485042	KM485160	KM484946

Abbreviations: **ATCC**: American Type Culture Collection, Virginia, USA; **CBS**: Westerdijk Fungal Biodiversity Institute, Utrecht, Netherlands; **CMU**: Chiang Mai University, Chiang Mai, Thailand; **CPC**: Culture Collection of Pedro Crous, Netherlands; **KUN-HKAS**: the Herbarium of Cryptogams Kunming Institute of Botany Academia Sinica, Yunnan, P.R. China; **HKUCC**: Hong

Kong University Culture Collection, Hong Kong, P.R. China; **IFRDCC**: Fungal Research & Development Centre Culture Collection, P.R. China; **KUMCC**: Kunming Culture Collection, Yunnan, P.R. China; **MFLU**: the Herbarium of Mae Fah Luang University, Chiang Rai, Thailand; **MFLUCC**: Mae Fah Luang University Culture Collection, Chiang Rai, Thailand.

Nomenclature

The electronic version of this article in Portable Document Format (PDF) in a work with an ISSN or ISBN will represent a published work according to the International Code of Nomenclature for algae, fungi, and plants, and hence the new names contained in the electronic publication of a PLOS ONE article are effectively published under that Code from the electronic edition alone, so there is no longer any need to provide printed copies.

In addition, new names contained in this work have been submitted to Index Fungorum from where they will be made available to the Global Names Index. The unique Index Fungorum number can be resolved, and the associated information viewed through any standard web browser by appending the Index Fungorum number contained in this publication to the prefix www.indexfungorum.org/. The online version of this work is archived and available from the following digital repositories: PubMed Central and LOCKSS.

Compliance with Ethical Standards

There is no conflict of interest (financial or non-financial) and all authors have agreed to submission of paper. The authors also declare that they have no conflict of interest and confirm that the field studies did not involve endangered or protected species.

Results

Molecular phylogeny

Based on the results from the nucleotide BLAST search tool of LSU and SSU sequences, our new strains (KUMCC 20-0213 and KUN-HKAS 107677) are closely related to species of *Ophioceras*, whereas ITS sequence revealed that our new strains are closely related to the unidentified fungal endophyte isolate 4583 (86.79% similarity) and other taxa in Magnaporthales. The concatenated LSU-RPB1-ITS dataset included 2594 total characters with gaps (LSU: 1–905 bp, RPB1: 906–1877 bp, ITS: 1878–2594 bp). The best scoring ML tree with the final ML optimization likelihood value of -23570.931644 (ln) was selected to represent the phylogenetic relationships of taxa in Magnaporthales (Fig. 1). All free model parameters were estimated using the GTRGAMMA model, with 1354 distinct alignment patterns and 40.89% of undetermined characters or gaps. Estimated base frequencies were as follows: A = 0.247576, C = 0.255381, G = 0.292293, T = 0.204750, with substitution rates AC = 1.508478, AG = 2.781197, AT = 1.835341, CG = 0.983246, CT = 6.427316, GT = 1.000000. The Tree-Length = 7.381586 and the gamma distribution shape parameter α = 0.605644. The evaluation of Bayesian posterior probabilities (BYPP) from MCMC was carried out with the final average standard deviation of split frequencies reached 0.009466.

The tree topology from ML analysis showed similar results with the BI analysis and comparing LSU-ITS, and LSU-RPB1-ITS phylograms also revealed similarities in overall topologies (Figs. 1 and S2). Thus, we will use the LSU-RPB1-ITS topology for further discussion. Five families of Magnaporthales were included in the presented phylogenetic analyses viz. Ceratosphaeriaceae, Magnaporthaceae, Ophioceraceae, Pseudohalonectriaceae and

Pyriculariaceae. These five families formed well-resolved monophyletic clades within Magnaporthales with significant support (greater than 70% ML and 0.95 PP) in our combined gene analyses (Figs. 1 and S2). Ophioceraceae has a close relationship with Ceratosphaeriaceae and Pseudohalonectriaceae. However, the phylogenetic relationships of these three families are not well resolved and pending further clarification.

Phylogenetic analyses of the LSU-RPB1-ITS sequence matrix revealed that the investigated specimen (KUN-HKAS 107677) and its pure culture (KUMCC 20-0213) are grouped together and form an independent lineage basal to *Ophioceras* in Ophioceraceae with high statistical support (100% ML, 1.00 PP; Fig. 1). Considering the phylogenetic results and morphology, we propose a novel species, *Ophioceras sichuanense*, occurring on submerged bamboo in Sichuan Province, China.

Ceratosphaeria castillensis (SMH 1865) formed a robust clade with *C. rhizomorpha* (GKM 1262) (100% ML, 1.00 PP; Fig. 1) within *Ophioceras* (81% ML, 0.91 PP; Fig. 1). *Ceratosphaeria castillensis* (SMH 1865) and *C. rhizomorpha* (GKM 1262) clustered with *Ophioceras aquaticus* (IFRDCC 3091, MFLUCC 16-0906), *O. dolichostomum* (CMURp50), *O. hongkongense* (HKUCC 3624), *O. submersum* (MFLUCC 18-0211) and *O. commune* (KUN-HKAS 92569, KUN-HKAS 92587, KUN-HKAS 92590, KUN-HKAS 92640) in our all analyses (Figs. 1, S1 and S2) and separated distantly from taxa in Magnaporthaceae. Thus, *Ceratosphaerella* is treated as a synonym of *Ophioceras*, the prior introduced genus, in Ophioceraceae.

Fig 1. Maximum likelihood tree ~~revealed by RAxML analysis~~ based on a combined LSU, RPB1 and ITS sequence matrix for taxa in Magnaporthales. Bootstrap support values for ML equal to or higher than 70% and the Bayesian posterior probabilities equal to or higher than 0.90 PP are defined above the nodes as ML/PP. Ex-type strains are in black bold, and

new species and new combinations are indicated in red bold.

Taxonomy

Ophioceraceae Klaubauf, Lebrun & Crous, Studies in mycology 79: 103 [1]

Type genus: *Ophioceras* Sacc.

Notes – To date, Ophioceraceae includes a single genus, *Ophioceras*. In the present phylogenetic study, Ophioceraceae formed a stable clade within Magnaporthales and distinguished from other families of Magnaporthales.

Ophioceras Sacc., Sylloge Fungorum 2: 358 [7] emend. H.B. Jiang & Phookamsak [urn:lsid:indexfungorum.org:names:3595]

Facesoffungi number: FoF01255

Synonym: *Ceratosphaerella* Huhndorf, Greif, Mugambi & A.N. Mill., Mycologia 100(6): 941 [8].

Type species: *Ophioceras dolichostomum* (Berk. & M.A. Curtis) Sacc.

Saprobic on bamboo, palm, bark or wood, and other herbaceous plants from aquatic or terrestrial environments. **Sexual morph**: *Ascomata* black, perithecial, immersed to superficial, scattered or gregarious, globose to subglobose, or ampulliform, glabrous with ostiolar necks, somewhat forming uni- to multi-loculate pseudostromata. *Pseudostromata if present*: locules immersed in pseudostroma, dark brown to black, subglobose to ampulliform, or irregular in shape, with a long, cylindrical, black, brittle, curved or straight, periphysate neck. *Peridium* composed of several layers, of pseudoparenchymatous cells, arranged in *textura angularis*, inner layers composed of hyaline, elongate cells, with compressed, dark brown to black cells towards the outer

layers. *Paraphyses* filiform, hyaline, unbranched, septate, broad at the base, tapering at the tip. *Asci* unitunicate, 8-spored, subcylindrical to acerose or clavate, pedicellate or sessile, with a refractive J-, apical ring. *Ascospores* filiform or narrow fusiform, with rounded ends, slightly curved or sigmoidal, hyaline, pale brown or olivaceous, aseptate or septate, with or without guttulate, lacking a sheath. **Asexual morph**: Hyphomycetous, Didymobotryum-like. *Colony on substrates* dark brown to black, rhizomorphic-like threads, radiated from central of clustered ascomata on patched subiculum. *Synnemata* formed on rhizomorphic strands, dichotomously branched hyphae, straight or flexuous, branched, lighter brown head, black in mass, with conidiophores at the apical region. *Conidiophores* elongate, septate, with dark brown bands at the septa, verrucose. *Conidiogenous cells* pale brown, cylindrical, tretic, integrated, terminal, verrucose. *Conidia* pale brown, with darker brown at the septa, ellipsoid to cylindrical, 1–3 transverse septa, verrucose [8].

Notes – *Ophioceras* occurs on a wide range of hosts mainly distributed in America, Asia, Africa and Oceania [2,5,8,9,12,31,32,33,34,35,36,37,38,39,40,41,42,43,44,45,46,47,48,49]. To date, only *O. bambusae* and *O. guttulatum* have been reported from bamboo [47,49,50]. Eleven *Ophioceras* species have been reported from freshwater [2,10], of which nine species were found in China [10,32,46,49]. In this study, *O. sichuanense* is introduced as the second species occurring on submerged decaying branches of bamboo in China.

Ceratosphaerella is morphologically different from *Ophioceras* in having clavate asci and hyaline to pale brown, narrow fusiform ascospores, whereas *Ophioceras* has subcylindrical to acerose asci and hyaline to olivaceous, filiform ascospores [1,8]. However, *Ophioceras* resembles *Ceratosphaerella* in the ascomatal morphology and is also supported by phylogenetic analyses. Through ML and BI phylogenetic analyses based on a concatenated LSU-RPB1-ITS sequence

matrix (Fig. 1), *C. castillensis* (SMH 1865), which was previously treated in Magnaporthaceae, is phylogenetically closely related to *C. rhizomorpha* and clustered within *Ophioceras* in Ophioceraceae. Therefore, we treat *Ceratosphaerella* as a synonym of *Ophioceras* instead of a genus in Ophioceraceae. Using a morpho-phylogenetic approach, *Ophioceras castillensis* comb. nov. and *O. rhizomorpha* comb. nov. are hereby introduced.

Ophioceras sichuanense H.B. Jiang, Phookamsak & K.D. Hyde, *sp. nov.* Fig. 2

[urn:lsid:indexfungorum.org:names:557956]

Facesoffungi number: FoF09404

Etymology – The specific epithet "*sichuanense*" refers to Sichuan Province, P.R. China, where the species was collected.

Holotype – KUN-HKAS 107677

Saprobic on decaying branches of bamboo submerged in freshwater. **Sexual morph**: *Pseudostromata* 300–750 μm diam., 230–350 μm high (excluding necks), black, scattered, solitary, semi-immersed to superficial, 1–5-loculate, glabrous, ostiolate, papillate, carbonaceous. *Locules* 150–300 μm diam., 150–250 μm high (excluding necks), immersed within pseudostroma, clustered, subglobose to ampulliform, blackened, with a long black, periphysate neck, up to 1 cm. *Peridium* 20–35 μm wide, thick-walled, composed of several layers, of flattened to broad, pseudoparenchymatous cells, arranged in *textura angularis* to *textura prismatica*, inner layers composed of hyaline cells, outer layers composed of dark brown to black pseudoparenchymatous cells. *Paraphyses* 4–8 μm wide, filiform, hyaline, indistinct septate, unbranched, slightly rough with small guttules, broad at the base, tapering toward the tip. *Asci* 90–115 × 5–6.5 μm ($\bar{x}$ = 103 × 5.8 μm, n = 20), 8-spored, unitunicate, cylindrical, sessile to subsessile, with short broad bulb-like

at the base, apically rounded with a J-, apical ring. *Ascospores* 80–90 × 1–1.5 μm ($\overline{x}$ = 85 × 1.3 μm, n = 15), overlapping, or in parallel, hyaline, filiform, slightly curved to sigmoidal, thin-walled, aseptate, smooth-walled, multi-guttulate. **Asexual morph**: not observed.

Culture characteristics: Ascospores germinated on PDA within 24 hours at room temperature under normal condition. Mycelium superficial to immersed in agar medium, branched, septate, smooth hyphae. Colonies growing slowly on PDA, reaching 20 mm in two weeks, cottony, circular, with entire edge, raised, white from above and below.

Material examined: P.R. China, Sichuan Province, Yibin City, Shunan Artificial Bamboo Forest, on submerged decaying branches of bamboo, 25 July 2019, H.B. Jiang & R. Phookamsak, SC011 (KUN-HKAS 107677, holotype), ex-type living culture, KUMCC 20-0213.

GenBank accession numbers: ITS = MT995045, LSU = MT995046, SSU = MT995047 (KUMCC 20-0213; from pure culture); ITS = MW057779, LSU = MW057782, SSU = MW057847, TEF1-α = MW082017 (KUN-HKAS 107677; from fruiting bodies).

Notes – *Ophioceras sichuanense* can be distinguished from other *Ophioceras* species because it forms multi-loculate pseudostromata. *Ophioceras sichuanense* resembles *O. guttulatum*, *O. leptosporum* and *O. tenuisporum*. However, *O. sichuanense* differs from *O. guttulatum* in having smaller asci and ascospores (asci: 90–115 × 5–6.5 μm vs. 130–160 × 14–17 μm; ascospores: 80–90 × 1–1.5 μm vs. 100–128 × 4–5 μm) [46]. *Ophioceras sichuanense* resembles *O. leptosporum* and *O. tenuisporum* due to the size ranges of asci and ascospores and extremely long ostiolar necks. However, the species differs from the latter two species in their ascospore septation (Table 2). Although *O. bambusae* and *O. sichuanense* were collected from bamboos, *O. sichuanense* has longer asci and ascospores (asci: 90–115 × 5–6.5 μm vs. 90–95 × 5.5–6.5 μm; ascospores: 80–90 × 1–1.5 μm vs. 75–80 × 1.5 μm) [50]. Present phylogenetic analyses show *O.*

sichuanense formed a clade basal to *Ophioceras* and close to *O. chiangdaoense* and *O. leptosporum*. *Ophioceras sichuanense* and *O. chiangdaoense* are different in the dimensions of the ostiolar neck, asci and ascospores (Table 2).

Fig 2. *Ophioceras sichuanense* (KUN-HKAS 107677, holotype). a, b Pseudostromata with long ostiolar necks on host. c–e Section through pseudostromata. f Ascoma. g Ostiole. h Apical part of neck. i Peridium. j–l Asci. m Ascospores. n Paraphyses. Scale bars: d, e = 200 µm, f–h = 50 µm, i = 30 µm, j–n = 20 µm.

Ophioceras castillensis (C.L. Sm.) H.B. Jiang, Phookamsak & K.D. Hyde, *comb. nov.* Fig. 3

[urn:lsid:indexfungorum.org:names:557957]

Facesoffungi number: FoF09405

Basionym: *Ceratosphaeria castillensis* C.L. Sm., Bull. Lab. Nat. Hist. Iowa State Univ. 2: 403 (1893)

Synonym: *Ceratosphaerella castillensis* (C.L. Sm.) Huhndorf, Greif, Mugambi & A.N. Mill., Mycologia 100(6): 944 [8]

Type information: Nicaragua, Castillo Viejo, on bark, Feb–Mar 1893, C.L. Smith, Central American Fungi 13, (isotype, NY).

Detailed description and illustration: see Huhndorf et al. [8].

Known hosts/ habitat and distribution: Saprobic on bark or wood in terrestrial habitat. To date, *Ophioceras castillensis* is only reported from Costa Rica, Nicaragua, and Puerto Rico [8].

Notes – In this study, *Ceratosphaerella castillensis* is transferred to *Ophioceras* as *O. castillensis* based on a concatenated LSU-RPB1-ITS analyses coupled with morphological similarity of the ascomata and ascomatal wall which is typical *Ophioceras*. *Ophioceras castillensis*

can be separated from other *Ophioceras* species in forming ascomata on large clusters, superficial on sparse, subicular hyphae and having clavate asci and hyaline to pale brown, fusiform, 3-septate ascospores [8]. Detailed morphological comparison and taxa habitats in *Ophioceras* are described in Table 2.

Fig 3. *Ophioceras castillensis* (redrawn from Huhndorf et al. [8], NY isotype). a, b Ascomata. c Ascus. d Apical ring. e Ascospores. f Peridium. g Paraphyses. Scale bars: a = 1 mm, b = 200 µm, f = 20 µm, c, e, g = 10 µm, d = 5 µm.

Ophioceras rhizomorpha (Huhndorf & Mugambi) H.B. Jiang, Phookamsak & K.D. Hyde, *comb. nov.* Fig. 4

[urn:lsid:indexfungorum.org:names:557958]

Facesoffungi number: FoF09406

Basionym: *Ceratosphaerella rhizomorpha* Huhndorf & Mugambi, Mycologia 100(6): 944 [8]

Type information: Kenya, Kagamega National Park, on decaying wood on the ground, 17 January 2007, G.K. Mugambi, GKM1262 (holotype EA, isotype F).

Detailed description and illustration: see Huhndorf et al. [8].

Known hosts/ habitat and distribution: Saprobic on decaying wood of terrestrial habitat. To date, *Ophioceras rhizomorpha* is only reported from Kenya.

Notes – *Ophioceras rhizomorpha* was reported with a Didymobotryum-like asexual morph on a natural substrate [8]. The species resembles *O. castillensis* in forming ascomata on the large subicular, and having clavate asci and hyaline to pale brown, fusiform, 3-septate ascospores [8].

Ophiocera rhizomorpha, however, differs from *O. castillensis* in having larger ascomata, necks, peridia, paraphyses, asci and ascospores [8] (see Table 2).

Fig 4. *Ophioceras rhizomorpha* (redrawn from Huhndorf et al. [8], all from Mugambi 1262). a Ascomata and synnemata on the substrate. b Ascoma. c Peridium. d Ascus. e Apical ring. f Hyaline to pale brown ascospores. g Paraphyses. h, i Conidiophores. j Conidiogenous cell bearing conidium. k Conidium. Scale bars: a = 1 mm, b = 300 µm, c = 50 µm, g = 30 µm, d = 20 µm, i, k = 15 µm, f, h, j = 10 µm, e = 5 µm.

Discussion

Ophioceraceae currently accommodates only *Ophioceras*. Maharachchikumbura et al. [57] accommodated the family in Magnaporthales (Diaporthomycetidae, Sordariomycetes) based on literature review and phylogenetic analysis. The studies of fungal ranking based on molecular clock evidence showed that Ophioceraceae shares a common ancestor with Ceratosphaeriaceae at 65 MYA in Magnaporthales (130–190 MYA) and the subclass Diaporthomycetidae (247–250 MYA) was the youngest subclass in Sordariomycetes (130–250 MYA) [3,58]. However, the stem age and crown age of the familial ranking are not flexible depending on taxon sampling, gene selection in data matrix, models and parameters selection as well as the calibration points [58,59].

Ophioceraceae has limited taxon sampling, and most taxa in this family lack reliable protein coding genes to clarify phylogenetic affinities. For example, *Ophioceras arcuatisporum* (strains A9-1, A167-1B) has only SSU sequence data available in GenBank. In preliminary phylogenetic analysis of SSU sequence matrix, the species formed a stable clade within Magnaporthaceae rather

than Ophioceraceae. *Ophioceras arcuatisporum* needs to be re-visited and re-illustrated, incorporating details from molecular data.

Luo et al. [2] performed combined LSU and TEF1-α phylogenetic analyses to investigate the relationships of taxa in Magnaporthales. In their phylogeny, *Ceratosphaeria* grouped with *Pseudohalonectria* (Pseudohalonectriaceae) and separated from *Ophioceras* (Ophioceraceae). Based on the fact that *Ceratosphaeria* differs from *Pseudohalonectria* in having narrow cylindric-fusiform to filiform and longer ascospores, Ceratosphaeriaceae was thus introduced as a new family within Magnaporthales to accommodate *Ceratosphaeria* [2]. In the present study, we performed an updated phylogenetic tree based on a concatenated LSU-RPB1-ITS sequences and showed that *Ceratosphaeria* (Ceratosphaeriaceae) clustered with *Ophioceras* (Ophioceraceae) with low statistical support, suggesting that gene selection in the data matrix affects the tree topology at the familial levels in Magnaporthaceae. *Ceratosphaeria* is morphologically similar to *Ceratosphaerella* [8]. Although *Ceratosphaeria* clustered with *Ophioceras* with low statistical support, *Ceratosphaeria* possibly belongs to *Ophioceraceae*. However, the phylogenetic status of *Ceratosphaeria* needs to be clarified with more evidence in the future studies.

In the present study, we synonymize *Ceratosphaerella* under *Ophioceras* based on molecular phylogeny coupled with similar ascomatal morphology. Phylogenetic analyses based on the LSU sequence dataset (Fig. S1) and the concatenated LSU-ITS (Fig. S2) and the LSU-RPB1-ITS (Fig. 1) sequence datasets have always shown that *O. castillensis* (≡ *C. castillensis*) and *O. rhizomorpha* (≡ *C. rhizomorpha*) formed a stable clade within *Ophioceras*. *Ophioceras castillensis*, *O. rhizomorpha* and most *Ophioceras* species lack protein coding genes and other reliable genes to clarify phylogenetic placement as well as limited taxon sampling. The ex-type strain of *O. rhizomorpha* and the reference strain of *O. castillensis* were sequenced only for LSU locus. Hence,

more reliable gene loci (e.g., SSU, ITS, RPB1 and TEF1-α) from the ex-type strain of *O. rhizomorpha* should be obtained and the epitype of *O. castillensis* should be designated and incorporated with morpho-molecular based taxonomic treatment. Furthermore, the new collections and sequence data of taxa in *Ophioceras* are required to provide a better taxonomic resolution for robust species delineations in this genus.

Many genera in Magnaporthales have similar morphological characteristics with *Ophioceras* (Table 3). However, these genera are considered distinct genera based on phylogenetic investigations [1,2,8,60,61,62]. *Pseudohalonectria* (Pseudohalonectriaceae) is also similar to *Ophioceras* in its ascomata and asci. However, *Pseudohalonectria* varied in shape of ascospores, such as ellipsoidal, fusiform or filiform [30]. In the present study, *Pseudohalonectria* (Pseudohalonectriaceae) formed an independent lineage separate from other families in Magnaporthales; however, *Pseudohalonectria* could not be resolved at the species level such as in Perera et al. [30]. It may be because molecular data of most taxa in this genus are unavailable in GenBank database. Moreover, some sequences of *P. lignicola* deposited in GenBank are likely to be misidentified [30]. Therefore, sequences of *Pseudohalonectria* species used for phylogenetic analyses are limited.

Author Contributions

Conceptualization: Hong-Bo Jiang, Rungtiwa Phookamsak, Kevin D. Hyde, Saisamorn Lumyong.

Data curation: Hong-Bo Jiang.

Formal analysis: Hong-Bo Jiang, Rungtiwa Phookamsak.

Funding acquisition: Ali H. Bahkali, Abdallah M. Elgorban, Samantha C. Karunarathna, Rungtiwa Phookamsak, Saisamorn Lumyong.

Investigation: Hong-Bo Jiang, Rungtiwa Phookamsak, Er-Fu Yang, Kevin D. Hyde.

Methodology: Hong-Bo Jiang, Rungtiwa Phookamsak.

Project administration: Rungtiwa Phookamsak.

Supervision: Kevin D. Hyde, Saisamorn Lumyong, Rungtiwa Phookamsak, Pattana Kakumyan.

Writing – original draft: Hong-Bo Jiang, Er-Fu Yang, Rungtiwa Phookamsak.

Writing – review & editing: Kevin D. Hyde, Samantha C. Karunarathna, Pattana Kakumyan, Rungtiwa Phookamsak, Saisamorn Lumyong.

Acknowledgements

The authors gracefully thank the Biology Experimental Center, Germplasm Bank of Wild Species, Kunming Institute of Botany, Chinese Academy of Sciences for providing the facilities of molecular laboratory. We also thank Shaun Pennycook from Manaaki Whenua - Landcare Research for his assistance in naming the new species in the genus *Ophioceras*. Austin G. Smith is thanked for the English proofreading of this manuscript. Hong-Bo Jiang would like to thank Mae Fah Luang University for Ph.D scholarship.

Table 2. **Synopsis of *Ophioceras* species.**

Species name	Ascomata	Asci	Ascospores	Habitat	Origin	Host/substrate	References
Ophioceras aquaticus	310–620 µm diam., 1-loculate, superficial to submerged, with a 500–800 µm long neck	85–100 × 9–10 µm, cylindrical	42–68 × 3–4 µm, filiform, slightly acute at each end, falcate, sigmoid, hyaline, 3–5-septate	Submerged	China: Yunnan	Wood	[2,10]
O. arcuatisporum	313–324 × 252–340 µm, 1-loculate, superficial to immersed, with a long neck, up to 800 µm	276–307 × 15–20 µm, fusoid to narrowly cylindrical	170–239 × 4–7 µm, narrowly fusoid to cylindrical, falcate, hyaline to pale orange, 5–12-septate	Submerged	USA: Minnesota	*Typha* sp., herbaceous debris, grass	[42]
O. bambusae	1 mm long, 2/3 mm diam., 1-loculate, immersed, with a 2–2.5 mm long neck	90–95 × 5.5–6.5 µm, cylindrical	75–80 × 1.5 µm, filiform, with both blunt ends, curved, hyaline, indistinctly septate	Terrestrial	Indonesia: Java	Bamboo	[50]
O. castillensis	525–650 µm diam., 1-loculate, superficial,	70–90 × 10–14 µm, clavate	29–40 × 4–5.5 µm, narrowly fusiform,	Terrestrial	Nicaragua	Bark and wood	[8]

	with a 250–400 µm long neck		slightly curved, hyaline to pale brown, 3-septate				
O. cecropiae	200–250 µm diam., 1-loculate, immersed, with a long neck, up to 2 mm	75–90 × 6.5–7.5 µm, cylindrical to subfusoid	60–70 × 2 µm, filiform, straight to slightly curved, hyaline, septate	Terrestrial	Venezuela	*Cecropia* sp.	[63]
O. chiangdaoense	200–310 × 170–310 µm, 1-loculate, immersed, with a 93–273 µm long neck	85–125 × 11–17 µm, cylindrical	54–75.5 × 4–5.5 µm, filiform, falcate, fusoid at both ends, hyaline, 3-septate	Terrestrial	Thailand: Chiang Mai	Decaying leaves of *Dracaena loureiroi*	[9]
O. commune	150–350 × 260–400 µm, 1-loculate, immersed to superficial, with a 375–1660 µm long neck	64–118 × 4–12 µm, cylindrical	50–110 × 2 µm, filiform, arcuate or sigmoidal, hyaline, 3–7-septate	Submerged	Panama: Barro Colorado Island	Wood, herbaceous debris	[2,42]
O. dolichostomum	500 µm diam., 1-loculate, immersed, with a 1–5 mm long neck	100–130 × 8–12 µm, cylindrical	94–110 × 2–3 µm, filiform, falcate, sigmoid, hyaline, 3–7-septate	Submerged	USA: Florida	Dead wood	[2,37]

O. filiforme	3–5 mm long, 150–180 µm diam., 1-loculate, immersed, erumpent to superficial, with a long neck	100–120 × 10–13 µm, clavate or fusoid	80–100 × 3–3.5 µm, filiform, hyaline to yellowish, multi-septate	Terrestrial	Indonesia: Java	Rotten leaf sheaths of *Amomum* sp.	[64]
O. fusiforme	360–500 × 330–450 µm, 1-loculate, immersed to erumpent, with a 250–800 µm long neck	70–112 × 6–12 µm, cylindrical	64–104 × 1.5–3 µm, filiform, fusoid, tapering at both ends, straight to falcate, 3–5-septate	Submerged	USA: Indiana	Decorticated woody debris	[42]
O. guttulatum	400–600 × 1200–1800 µm, 1-loculate, superficial to immersed, with a 500–1500 µm long neck	130–160 × 14–17 µm, broadly cylindrical	100–128 × 4–5 µm, cylindrical, falcate, pale yellow to hyaline, 3–5-septate	Submerged	China: Hong Kong	Wood	[46]
O. hongkongense	500–640 × 700–800 µm, 1-loculate, superficial to immersed, with a more than 600 µm long neck	100–125 × 12–14 µm, elongated fusoid to broadly	72–101 × 3.5–4.5 µm, cylindrical, falcate, tapered at both ends, hyaline, 3–5-septate	Submerged	China: Hong Kong	Wood	[46]

		cylindrical					
O. indicus	400–650 µm diam., 1-loculate, immersed, with a long neck, up to 1.5 mm	65–90 × 8.5–11.5 µm, cylindrical to subfusoid	60–85 × 2.5–3.5 µm, filiform, tapering towards base, slightly curved, hyaline to subhyaline, 7–10-septate	Terrestrial	India: New Delhi	Dried twigs of *Ficus infectoria*	[38]
O. leptosporum	250–300 µm diam., 1-loculate, immersed or superficial, with a 1–2 mm long neck	70–95 × 5–6 µm, cylindrical	70–80 × 1–1.5 µm, filiform, apex rounded, base acute, hyaline to faintly tinted, straight to slightly curved or sigmoid, 3–7-septate	Submerged	UK: Exeter	Rotten stems of *Umbelliferae* sp.	[4]
O. miyazakiense	Data unavailable	Data unavailable	Data unavailable	Terrestrial	Japan: Kyushu	Decaying litter	[65]
O. palmae	164–320 × 244–288 µm, 1-loculate, partly immersed, with a 180–376 µm long neck	76–96 × 10–14 µm, broadly cylindrical	79–90 × 1.2–2 µm, filiform, tapering at both ends, sigmoid, hyaline, 5-septate	Terrestrial	Philippines: Mt. Makiling	*Calamus ornatus*	[46]

O. parasiticum	600–800 µm diam.	100–140 × 9–11 µm	48–70 × 2.5–3.3 µm, 3–9-septate	Terrestrial (parasite)	China	Data unavailable	[9]
O. petrakii	600–750 × 555–675 µm, 1-loculate, immersed, with a neck	171–182 × 12–15 µm, cylindrical	152–171 × 3–4 µm, filiform, apex rounded, base acute, slightly curved or sigmoid, hyaline, multi-septate	Terrestrial	India: Karnataka	Dead stems of *Vitex negundo*	[36]
O. rhizomorpha	500–900 × 500–750 µm, 1-loculate, superficial, with a 300–600 µm long neck	115–145 × 13–16 µm, clavate	39–49 × 3.5–4.5 µm, narrowly fusiform, slightly curved, hyaline to pale brown, 3-septate	Terrestrial	Kenya	Bark or wood	[8]
O. sichuanense	230–350 × 300–750 µm, pseudostromatic, 1–5-loculate, semi-immersed to superficial, with long necks, up to 1 cm	90–115 × 5–6.5 µm, cylindrical	80–90 × 1–1.5 µm, filiform, slightly curved to sigmoidal, hyaline, aseptate	Submerged	China: Sichuan	Decaying branches of bamboo	This study
O. sorghi	300–400 µm diam., 1-	85–110 × 12–	75–95 × 3–4 µm,	Terrestrial	Central	*Sorghum*	[4,34]

	loculate, immersed, with a 350–700 µm long neck	14 µm, cylindrical to clavate	filiform, cylindrical, with rounded apex and slightly thinner rounded base, slightly curved, hyaline, 3–12-septate		African Republic: M' Baiki	*vulgare*	
O. submersum	300–400 × 500–600 µm, 1-loculate, immersed, with a long neck	115–137 × 10–11 µm, cylindrical	87–109 × 3–4 µm, filiform, rounded at both ends, slightly curved or sigmoid, hyaline, multi-septate	Submerged	Thailand	Wood	[2]
O. tambopataense	Data unavailable	Data unavailable	Data unavailable	Terrestrial	Peru	Decaying leaf of palm	[65]
O. tenuisporum	240–625 × 260–775 µm, 1-loculate, superficial to partially immersed, with a long neck, up to 20 mm	82–114 × 4–6 µm, cylindrical to narrowly fusoid	66–94 × 1–1.5 µm, filiform, more broadly rounded at one end than the other, curved to sigmoid, hyaline, 3-septate	Submerged	Panama: Barro Colorado Island	Twig	[42]

O. venezuelense	730–890 × 745–868 µm, 1-loculate, partially immersed to superficial, with a 250–800 µm long neck	148–180 × 11–18 µm, cylindrical to narrowly fusoid	130–158 × 2–4 µm, filiform, falcate, more broadly rounded at one end than the other, straight to slightly curved, hyaline, (4)–5–(6)-septate	Submerged	Venezuela: Portuguesa	Wood, herbaceous debris	[42]
O. zeae	450–650 µm diam., 1-loculate, superficial or partially immersed, with a 350–900 µm long neck	55–65 × 7–8 µm, cylindrical to narrowly fusoid	39–50 × 2.3–2.5 µm, cylindrical to fusoid, with rounded ends, straight or slightly curved, hyaline, 3-septate	Terrestrial	Central African Republic: Boukoko	Dead *Zea mays*	[4,33]

Table 3. **Morphological comparisons of similar genera to *Ophioceras*.**

Generic name	Ascomata	Asci	Ascospores	References
Aquafiliformis (Magnaporthaceae)	Globose to subglobose, with long beak	Cylindrical to clavate, with an inconspicuous apical ring	Aseptate, filiform, hyaline	[2]
Ceratosphaeria (Ceratosphaeriaceae)	Globose to pyriform, with a long black or yellow neck	Cylindrical, with a conspicuous, non-amyloid, apical ring	Multi-septate, filiform, hyaline	[2]
Gaeumannomycella (Magnaporthaceae)	Subglobose to elliptical, with a lateral, central cylindrical neck	Cylindrical to elongated clavate, apical ring not observed	0–3-septate, narrowly fusiform, hyaline	[62]
Gaeumannomyces (Magnaporthaceae)	Subglobose to elliptical, with a cylindrical neck	Cylindrical to elongated clavate, with an apical refringent ring	Indistinctly septate, filiform, hyaline to pale brown	[61]
Kohlmeyeriopsis (Magnaporthaceae)	Ellipsoid, with a long cylindrical periphysate neck	Fusoid to cylindrical, with a large apical ring	Indistinctly septate, filamentous, tapering towards the base, hyaline	[1,66]
Muraeriata (Magnaporthaceae)	Lageniform to globose, with long beak	Cylindrical to ventricose, with a tall, narrow, apical ring	3-septate, narrowly fusiform, ends slightly curved, hyaline	[8]
Neogaeumannomyces	Globose to subglobose, with a	Cylindrical, with an apical	2–3-septate, filiform to long	[60]

(Magnaporthaceae)	long, periphysate neck	ring	fusiform, hyaline	
Ophioceras (Ophioceraceae)	Subglobose to ampulliform, with a long cylindrical periphysate neck	Subcylindrical to narrowly fusoid or clavate asci with a small, refractive, non-amyloid apical ring	Aseptate to multi-septate, filiform, narrowly fusiform, hyaline pale brown or olivaceous	[2,8, this study]
Slopeiomyces (Magnaporthaceae)	Globose, with periphysate neck bearing hyphae	Clavate, with a non-amyloid apical ring	3–4-septate cylindrical to fusoid, tapering somewhat to base, hyaline	[1]

References

1. Klaubauf S, Tharreau D, Fournier E, Groenewald JZ, Crous PW, de Vries RP, et al. Resolving the polyphyletic nature of *Pyricularia* (Pyriculariaceae). Stud Mycol. 2014;79:85–120. doi: 10.1016/j.simyco.2014.09.004
2. Luo ZL, Hyde KD, Liu JK, Maharachchikumbura SSN, Jeewon R, Bao DF, et al. Freshwater Sordariomycetes. Fungal Divers. 2019;99:451–660. doi: 10.1007/s13225-019-00438-1
3. Hyde KD, Norphanphoun C, Maharachchikumbura SSN, Bhat DJ, Jones EBG, Bundhun D, et al. Refined families of Sordariomycetes. Mycosphere. 2020;11:305–1059. doi: 10.1017/10.5943/mycosphere/11/1/7
4. Walker J. *Gaeumannomyces*, *Linocarpon*, *Ophiobolus* and several other genera of scolecospored Ascomycetes and *Phialophora* conidial states, with a note on hyphopodia. Mycotaxon. 1980;11:1–129.
5. Chen W, Shearer CA, Crane JL. Phylogeny of *Ophioceras* spp. based on morphological and molecular data. Mycologia. 1999;91:84–94. doi: 10.1080/00275514.1999.12060995
6. Wijayawardene NN, Hyde KD, Rajeshkumar KC, Hawksworth DL, Madrid H, Kirk PM, et al. Notes for genera: Ascomycota. Fungal. Divers. 2017;86:1–594. doi: 10.1007/s13225-017-0386-0
7. Saccardo PA. Sylloge Pyrenomycetum, Vol. II. Sylloge Fungorum. 1883;2:1–813.
8. Huhndorf SM, Greif M, Mugambi GK, Miller AN. Two new genera in the Magnaporthaceae, a new addition to *Ceratosphaeria* and two new species of *Lentomitella*. Mycologia. 2008;100:940–955. doi: 10.3852/08-037
9. Thongkantha S, Jeewon R, Vijaykrishna D, Lumyong S, McKenzie EHC, Hyde KD. Molecular phylogeny of Magnaporthaceae (Sordariomycetes) with a new species,

Ophioceras chiangdaoense from *Dracaena loureiroi* in Thailand. Fungal Divers. 2009;34:157–173. doi: 10.1002/yea.1657

10. Hu DM, Cai L, Hyde KD. Three new ascomycetes from freshwater in China. Mycologia. 2012;104:1478–1489. doi: 10.3852/11-430

11. Index Fungorum. http://www.indexfungorum.org/Names/Names.asp. 2021 (accessed on 8 April 2021).

12. Hu HL, Fournier J, Jeewon R, Bahkali AH, Hyde KD. Revisiting the taxonomy of *Daruvedia bacillata*. Mycotaxon. 2010;114:135–144. doi: 10.5248/114.135

13. NCBI: National Center for Biotechnology Information (GenBank). https://www.ncbi.nlm.nih.gov/nuccore/. 2020 (accessed on 10 August 2020).

14. Hernández-Restrepo M, Bezerra JDP, Tan YP, Wiederhold N, Crous PW, Guarro J, et al. Re-evaluation of *Mycoleptodiscus* species and morphologically similar fungi. Persoonia. 2019;42:205–227. doi: 10.3767/persoonia.2019.42.08

15. Dai DQ, Phookamsak R, Wijayawardene NN, Li WJ, Bhat DJ, Xu JC, et al. Bambusicolous fungi. Fungal Divers. 2017;82:1–105. doi: 10.1007/s13225-016-0367-8

16. Jayasiri SC, Hyde KD, Ariyawansa HA, Bhat DJ, Buyck B, Cai L, et al. The Faces of Fungi database: fungal names linked with morphology, phylogeny and human impacts. Fungal Divers. 2015;74:3–18. doi: 10.1007/s13225-015-0351-8

17. White TJ, Bruns T, Lee SJWT, Taylor JW. Amplification and direct sequencing of fungal ribosomal RNA genes for phylogenetics. PCR—Protocols and Applications. 1990;18:315–322.

18. Vilgalys R, Hester M. Rapid genetic identification and mapping of enzymatically amplified ribosomal DNA from several *Cryptococcus* species. J Bacteriol. 1990;172:4238−4246. doi:

10.1128/jb.172.8.4238-4246.1990

19. Rehner SA. Primers for elongation factor 1-alpha (EF1-alpha). 2001. Available from: http://ocid.NACSE.ORG/research/deephyphae/EF1primer.pdf.

20. Jiang HB, Phookamsak R, Xu JC, Karunarathna SC, Mortimer PE, Hyde KD. Taxonomic and phylogenetic characterizations reveal three new species of *Mendogia* (Myriangiaceae, Myriangiales). Mycol Prog. 2020;19:41–51. doi: 10.1007/s11557-019-01540-z

21. Katoh K, Rozewicki J, Yamada KD. Mafft online service: multiple sequence alignment, interactive sequence choice and visualization. Brief Bioinform. 2017. doi: 10.1093/bib/bbx108

22. Hall T. BioEdit version 5.0.6. North Carolina State University. 2001.

23. Stamatakis A. RAxML-VI-HPC: maximum likelihood-based phylogenetic analyses with thousands of taxa and mixed models. Bioinformatics. 2006;22:2688–2690. doi: 10.1093/bioinformatics/btl446

24. Silvestro D, Michalak I. raxmlGUI: a graphical front-end for RAxML. Org Divers Evol. 2011;12:335–337. doi: 10.1007/s13127-011-0056-0

25. Miller MA, Pfeiffer W, Schwartz T. Creating the CIPRES Science Gateway for inference of large phylogenetic trees. In: Gateway Computing Environments Workshop 2010 (GCE). 2010; pp.1–8. doi: 10.1109/GCE.2010.5676129

26. Nylander J. MrModeltest2 v. 2.3 (Program for selecting DNA substitution models using PAUP*). Evolutionary Biology Centre, Uppsala. 2008.

27. Ronquist F, Huelsenbeck JP. MrBayes 3: Bayesian phylogenetic inference under mixed models. Bioinformatics. 2003;19:1572–1574. doi: 10.1093/bioinformatics/btg180

28. Rannala B, Yang Z. Probability distribution of molecular evolutionary trees: a new method

of phylogenetic inference. J Mol Evol. 1996;43:304–311. doi: 10.1007/BF02338839

29. Zhaxybayeva O, Gogarten JP. Bootstrap, Bayesian probability and maximum likelihood mapping: exploring new tools for comparative genome analyses. Genomics. 2002;3:1–15. doi: 10.1186/1471-2164-3-4

30. Perera RH, Maharachchikumbura SSN, Ariyawansa HA, Bahkali AH, Jones EBG, Al-Sadi AM, et al. Two new *Pseudohalonectria* species on beech cupules (*Fagus sylvatica*) and a new genus to accommodate *P. suthepensis*. Phytotaxa. 2016;278:115–131. doi: 10.11646/phytotaxa.278.2.2

31. Ellis JB, Everhart BM. The North American Pyrenomycetes. 1892;1–793.

32. Teng SJ. Notes on Sphaeriales from China. Sinensia. 1934;4:359–449.

33. Saccas AM. À propos de quelques champignons nouveaux parasites et saprophytes sur mais. Revue de Pathologe et d'Entomologie Agricole de France. 1951;30:161–196.

34. Saccas AM. Les champignons parasites des sorghos (Sorghum vulgare) et des penicillaires (Pennisetum typhoideum) en Afrique Equatoriale Française. Agronomie Tropicale. 1954;9:263–301.

35. Saccas AM. Étude de la Flore Cryptogamique des Caféirs en Afrique Centrale. 1981;1–522.

36. Tilak ST, Kale SB. Contribution to our knowledge of Ascomycetes of India - XX. Sydowia. 1969;23:17–23.

37. Conway KE, Barr ME. Classification of *Ophioceras dolichostomum*. Mycotaxon. 1977;5:376–380.

38. Lal SP. A new species of *Ophioceras* Sacc. from India. Kavaka. 1987;15:7–8.

39. Hyde KD. Tropical Australian freshwater fungi. IV. *Halosarpheia aquatica* sp. nov., *Garethjonesia lacunosispora* gen. et. sp. nov. and *Ophioceras dolichostomum*

(Ascomycetes). Aust Syst Bot. 1992;5:407–414. doi: 10.1071/SB9920407

40. Hyde KD, Goh TK. Fungi on submerged wood in the Riviere St Marie-Louis, the Seychelles. S Afr J Bot. 1998;64:330–336. doi: 10.1016/S0254-6299(15)30920-0

41. Hyde KD, Goh TK. Fungi on submerged wood in Lake Barrine, north Queensland, Australia. Mycol Res. 1998;102:739–749. doi: 10.1017/S0953756297005868

42. Shearer CA, Crane JL, Chen W. Freshwater Ascomycetes: *Ophioceras* species. Mycologia. 1999;91:145–156. doi: 10.1080/00275514.1999.12061004

43. Matsushima T. Matsushima Mycological Memoirs 9. *Matsushima*. Mycol Mem. 2001;10:1–214.

44. Tsui CKM, Hyde KD, Fukushima K. Fungi on submerged wood in the Koito River, Japan. Mycoscience. 2001;44:55–59. doi: 10.1007/s10267-002-0083-y

45. Tsui CKM, Hyde KD, Hodgkiss IJ. Longitudinal and temporal distribution of freshwater ascomycetes and dematiaceous hyphomycetes on submerged wood in the Lam Tsuen River, Hong Kong. J N Am Benthol Soc. 2001;20:533–549.

46. Tsui CKM, Leung YM, Hyde KD, Hodgkiss IJ. Three new *Ophioceras* species (Ascomycetes) from the tropics. Mycoscience. 2001;42:321–326. doi: 10.1007/BF02461213

47. Cai L, Ji KF, Hyde KD. Variation between freshwater and terrestrial fungal communities on decaying bamboo culms. Antonie Van Leeuwenhoek. 2006;89:293–301. doi: 10.1007/s10482-005-9030-1

48. Abdel-Aziz FA. Freshwater fungi from the River Nile, Egypt. Mycosphere. 2016;7:741–756. doi: 10.5943/mycosphere/7/6/4

49. Farr DF, Rossman AY. Fungal Databases, U.S. National Fungus Collections, ARS, USDA. 2020. Available online: https://nt.ars-grin.gov/fungaldatabases/ (accessed on 1 June 2020).

50. Höhnel FV. Fragmente zur Mykologie: VI. Mitteilung (Nr. 182 bis 288). Sitzungsberichte der Kaiserlichen Akademie der Wissenschaften Math.-naturw. Klasse Abteilung I. 1909;118:275–452.

51. Tsui CKM, Hyde KD, Hodgkiss IJ. Biodiversity of fungi on submerged wood in Hong Kong streams. Aquat Microb Ecol. 2000;21:289–298. doi: 10.3354/ame021289

52. Ho WH, Hyde KD, Hodgkiss IJ. Fungal communities on submerged wood from streams in Brunei, Hong Kong, and Malaysia. Mycol Res. 2001;105:1492–1501. doi: 10.1017/S095375620100507X

53. Ho WH, Yanna, Hyde KD, Hodgkiss IJ. Seasonality and sequential occurrence of fungi on wood submerged in Tai Po Kau Forest Stream, Hong Kong. In: Fungal Succession (eds. K.D. Hyde and E.B.G. Jones). Fungal Divers. 2002;10:21–43.

54. Cai L, Zhang KQ, McKenzie EHC, Hyde KD. Freshwater fungi from bamboo and wood submerged in the Liput River in the Philippines. Fungal Divers. 2003;13:1–12. doi: 10.1016/S1567-1356(03)00107-7

55. Species Fungorum. http://www.speciesfungorum.org/Names/Names.asp. 2021 (accessed on 8 April 2021).

56. Hyde KD, Jeewon R, Chen YJ, Bhunjun CS, Calabon MS, Jiang HB, et al. The numbers of fungi: is the descriptive curve flattening? Fungal Divers. 2020. doi: 10.1007/s13225-020-00458-2

57. Maharachchikumbura SSN, Hyde KD, Jones EBG, McKenzie EHC, Bhat DJ, Dayarathne MC, et al. Fungal Divers. 2016;79:1–317. doi: 10.1007/s13225-016-0369-6

58. Hyde KD, Maharachchikumbura SSN, Hongsanan S, Samarakoon MC, Lücking R, Pem D, et al. The ranking of fungi: a tribute to David L. Hawksworth on his 70th birthday. Fungal

Divers. 2017;84:1–23. doi: 10.1007/s13225-017-0383-3

59. Hongsanan S, Maharachchikumbura SSN, Hyde KD, Samarakoon MC, Jeewon R, Zhao Q, et al. An updated phylogeny of Sordariomycetes based on phylogenetic and molecular clock evidence. Fungal Divers. 2017;84:25–41. doi: 10.1007/s13225-017-0384-2

60. Liu JK, Hyde KD, Jones EBG, Ariyawansa HA, Bhat DJ, Boonmee S, et al. Fungal diversity notes 1–110: taxonomic and phylogenetic contributions to fungal species. Fungal Divers. 2015;72:1–197. doi: 10.1007/s13225-015-0324-y

61. Hernández-Restrepo M, Groenewald JZ, Elliott ML, Canning G, McMillan VE, Crous PW. Take-all or nothing. Stud Mycol. 2016;83:19–48. doi: 10.1016/j.simyco.2016.06.002

62. Crous PW, Schumacher RK, Akulov A, Thangavel R, Hernández-Restrepo M, Carnegie AJ, et al. New and interesting fungi. 2. Fungal Syst Evol. 2019;3:57–134. doi: 10.3114/fuse.2019.03.06

63. Müller E, Dennis RWG. Fungi venezuelani: VIII: Plectascales, Sphaeriales, Loculoascomycetes. Kew Bulletin. 1965;19:357–386. doi: 10.2307/4108161

64. Hennings P. Fungi monsunenses. Monsunia. 1900;1:137–174.

65. Matsushima T. *Matsushima.* Mycol Mem. 2003; 10. CD ROM [214 pp.+ incl. colour plates].

66. Kohlmeyer J, Volkmann-Kohlmeyer B, Eriksson OE. Fungi on *Juncus roemerianus*. 4. New marine ascomycetes. Mycologia. 1995;87:532–542. doi: 10.1080/00275514.1995.12026565

Supporting information

S1 Fig. RAxML tree generated from an analysis of the LSU matrix of taxa in Magnaporthales.

S2 Fig. RAxML tree generated from an analysis of the LSU-ITS matrix of taxa in Magnaporthales.

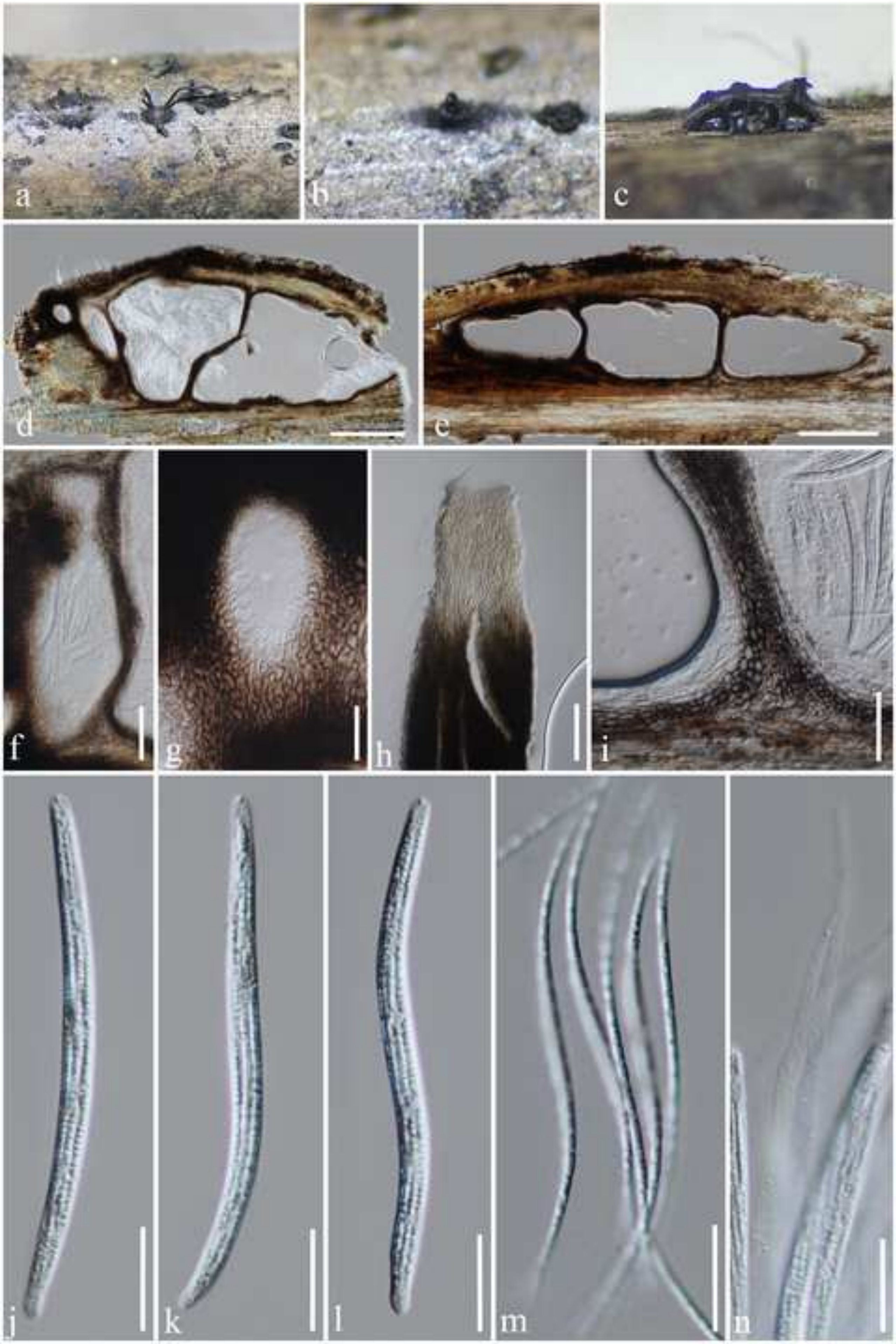
a
b
c
d
e
f
g
h
i
j
k
l
m
n

Figure Click here to access/download;Figure;Fig 3.tif

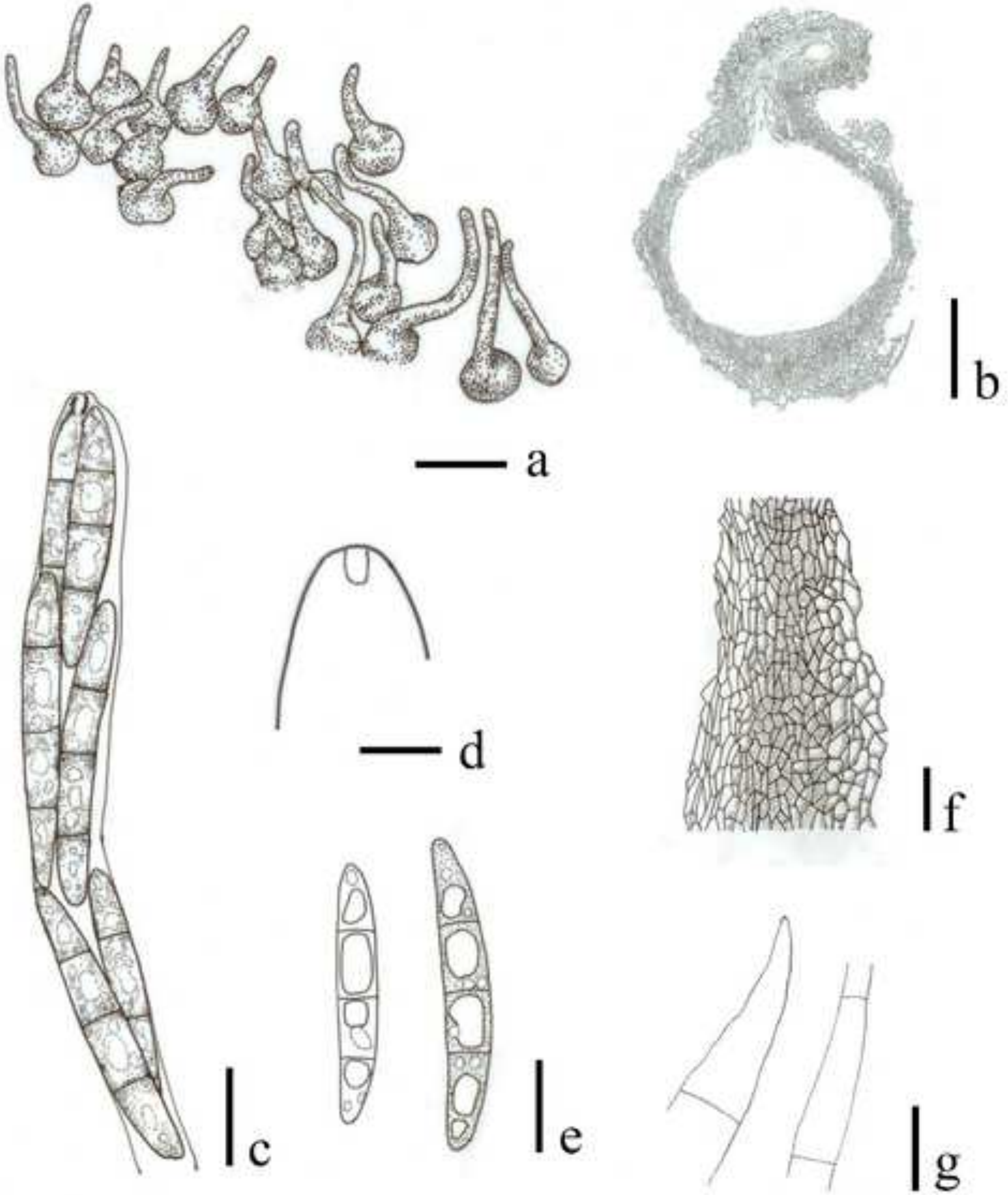

 Click here to access/download;Figure;Fig 4.tif

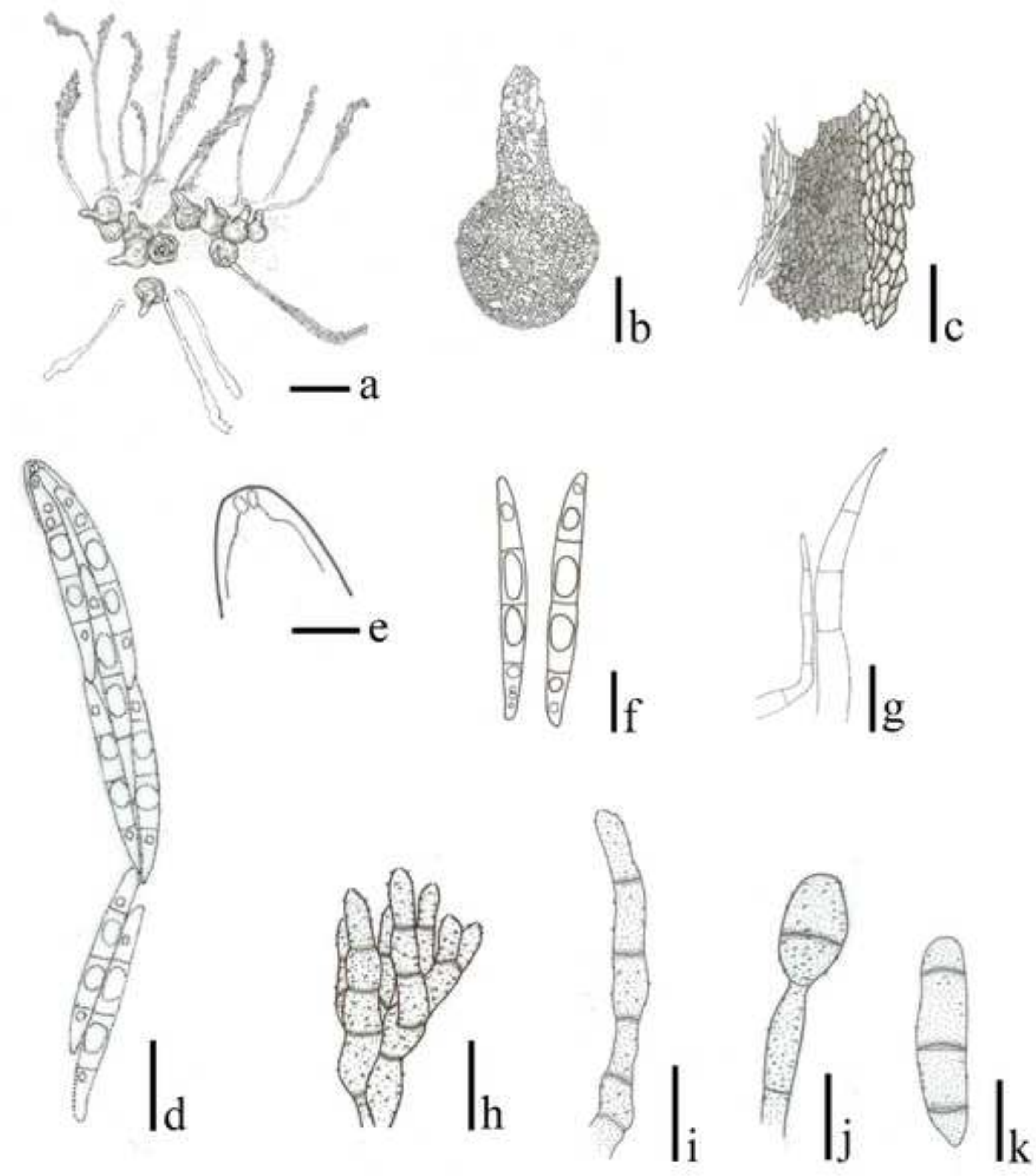

Figure

Click here to access/download;Figure;Fig1 updated.tif

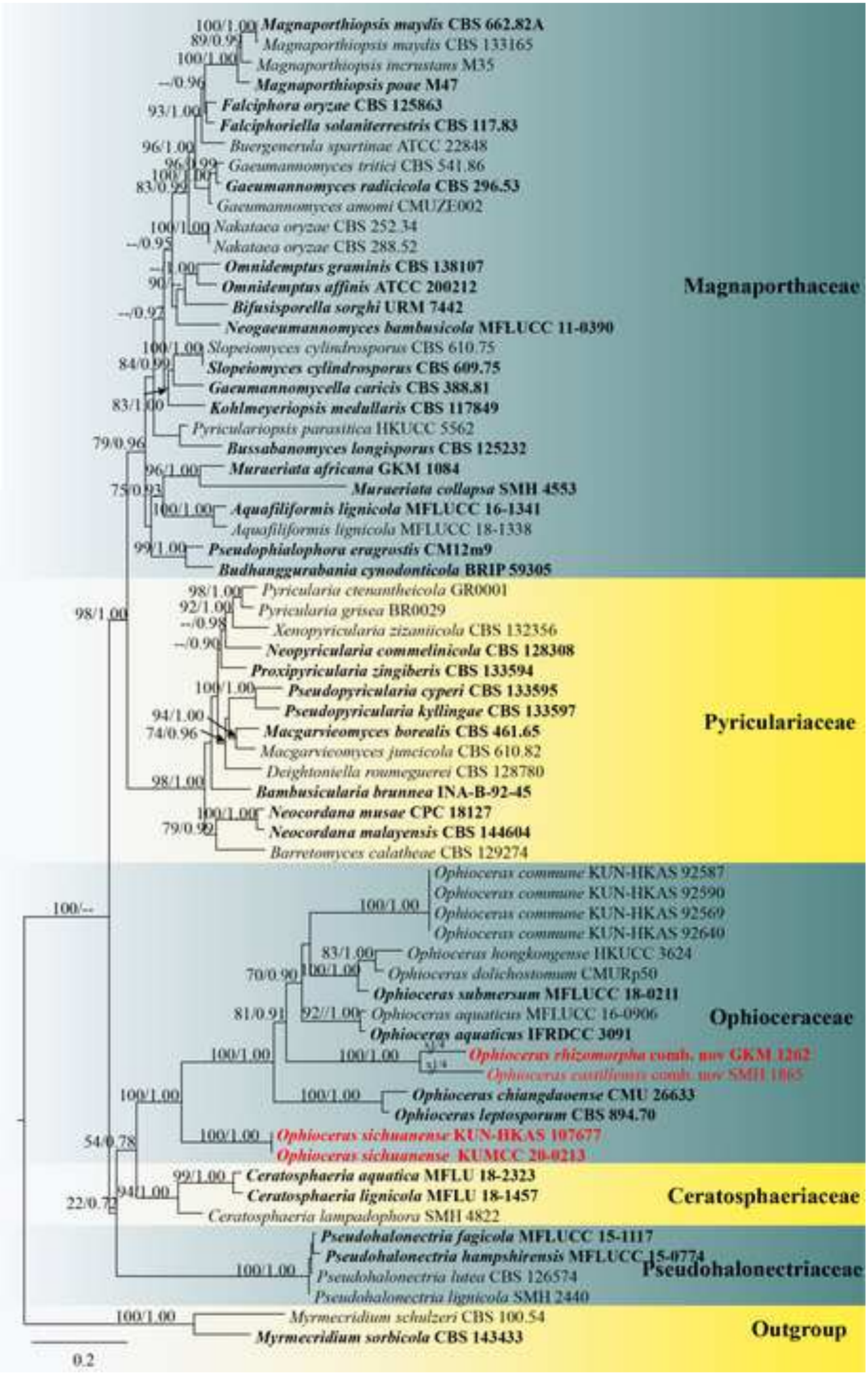

Figure

Click here to access/download
Supporting Information
Fig S1.tif

Figure

Click here to access/download
Supporting Information
Fig S2.tif

Revised Manuscript with Track Changes

Morphological and phylogenetic appraisal of *Ophioceras* (Ophioceraceae, Magnaporthales)

Hong-Bo Jiang[1,3,4,5,7], Kevin D. Hyde[1,3,4,8], Er-Fu Yang[3,7,9], Pattana Kakumyan[5], Ali H. Bahkali[8], Abdallah M. Elgorban[8], Samantha C. Karunarathna[3,6,7~~,10~~], Rungtiwa Phookamsak[1,3,6,7~~,10~~]*, Saisamorn Lumyong[1,2]*

[1] Research Center of Microbial Diversity and Sustainable Utilization, Faculty of Sciences, Chiang Mai University, Chiang Mai 50200, Thailand

[2] Academy of Science, The Royal Society of Thailand, Bangkok 10300, Thailand

[3] CAS Key Laboratory for Plant Diversity and Biogeography of East Asia, Kunming Institute of Botany, Chinese Academy of Sciences, Kunming 650201, Yunnan, P.R. China

[4] Center of Excellence in Fungal Research, Mae Fah Luang University, Chiang Rai 57100, Thailand

[5] School of Science, Mae Fah Luang University, Chiang Rai 57100, Thailand

[6] CIFOR-ICRAF China Program, World Agroforestry (ICRAF), Kunming 650201, Yunnan, China ~~East and Central Asia Regional Office, World Agroforestry Centre (ICRAF), Kunming 650201, Yunnan, P.R. China~~

[7] Honghe Center for Mountain Futures, Kunming Institute of Botany, Chinese Academy of Sciences, Honghe County 654400, Yunnan, P.R. China

[8] Department of Botany and Microbiology, College of Science, King Saud University, P.O. Box:

2455, Riyadh, 1145, Saudi Arabia

[9] Department of Biology, Faculty of Science, Chiang Mai University, Chiang Mai 50200, Thailand

[10] Centre for Mountain Futures (CMF), Kunming Institute of Botany, Kunming 650201, Yunnan, P.R. China

*Corresponding Author:

scboi009@gmail.com (SL)

jomjam.rp2@gmail.com (RP)

Abstract

Ophioceras is accommodated in the monotypic family Ophioceraceae (Magnaporthales, Sordariomycetes), and. tThe genus is delimited based on molecular data. *Ophioceras* forms black, immersed to superficial, subglobose ascomata, in some cases under a multi-loculate pseudostromata, with long cylindrical necks, . Asci are 8-spored, unitunicate, and 8-spored subcylindrical to clavate asci with a small, refractive, J-, apical ring, and ascospores are filiform or narrowly fusiform, hyaline to olivaceous or pale brown, aseptate or septate ascospores and without lack any sheaths or appendages. During an ongoing survey of bambusicolous fungi in southwest China and Thailand, we collected a submerged decaying culm branch of bamboo from Sichuan Province, China and an examined an *Ophioceras* species occurring on this specimensubstrate was observed and isolated. An *Ophioceras* taxon was We determined delimited the taxon based on morphological characteristics and the combined LSU, RPB1 and ITS sequence analyses. and*Ophioceras sichuanense* sp. nov., then is described as *Ophioceras*

Commented [JHB1]: Reviewer#2 comment: Are all this characters essential for the delimitation of the group? It would be nice if the authors could shorten this?

Commented [JHB2R1]: We have removed the generic description following the reviewer#1

Formatted: Not Highlight

sichuanense sp. nov.~~,~~ ~~introduced~~ in th~~e present study~~is paper. The species formed~~s~~ a well-supported clade basal to *Ophioceras* (100% ML, 1.00 PP). ~~Although *Ceratosphaerella* has little differences of ascospore characters from *Ophioceras*, *Ceratosphaerella* resembles *Ophioceras* in ascoma and ascus morphology~~ ~~*Ceratosphaerella* resembles *Ophioceras*, but differs in the ascospore characters.~~ Based on the updated phylogenetic tree of Magnaporthales, *Ceratosphaerella castillensis* (generic type) and *C. rhizomorpha* formed a clade within *Ophioceras* and morphologically resemble *Ophioceras*. Therefore, *Ceratosphaerella* is synonymized under *Ophioceras*. The phylogenetic relationships of *Ophioceras* are discussed in relation to morphological similarities of genera in Magnaporthales. The generic circumscription of *Ophioceras* is emended.

Keywords – 1 novel taxon, 2 new combinations, bambusicolous fungi, *Ceratosphaerella*, Sordariomycetes, taxonomy

Introduction

Klaubauf et al. [1] introduced the family Ophioceraceae to accommodate *Ophioceras* Sacc. which is typified by *O. dolichostomum* (Berk. & M.A. Curtis) Sacc. The family~~ial concept~~ is recognized as black, immersed to superficial, globose to subglobose, perithecial ascomata with long, periphysate necks, 8-spored, unitunicate, subcylindrical to narrowly fusoid asci with a J-,~~n~~ apical ring, and filiform, hyaline to olivaceous, septate ascospores without sheaths [1,2,3]. Species of Ophioceraceae are saprobes on wood and herbaceous plants in aquatic or terrestrial habitats [1,2,3,4,5,6].

Commented [MOU3]: Reviewer#2 comment: This info could be omitted in the abstract

Commented [MOU4R3]: We have removed this sentence from the abstract.

Commented [JHB5]: Reviewer comment: This info could be ommited in the abstract.

Commented [JHB6R5]:

Commented [JHB7]: Reviewer#2 comment: Are any of these species the type of genus?

Commented [JHB8R7]: *Ceratosphaerella castillensis* is the type of *Ceratosphaerella*. For *Ophioceras*, *Ophioceras dolichostomum* is the type. However, in this study, Ceratosphaerella and Ophioceras were found not much differences in these 2 genera from morphology, only have a little distance in ascospores character. In addition, from phylogeny, *Ceratosphaerella* is always clustered with *Ophioceras*, therefore, we synonymize *Ceratosphaerella* as *Ophioceras*.

Formatted: Not Highlight

Formatted: Not Highlight

Commented [JHB9]: Reviewer#2 comment: sound unusual

Commented [JHB10R9]: We have rewritten it.

Commented [JHB11]: Reviewer#2 comment: what about the J- in the abstract?

Commented [JHB12R11]: a J-, apical ring is included in the description.

Saccardo [7] introduced *Ophioceras* to accommodate taxa with immersed, subcarbonaceous, globose perithecia, with conical-cylindrical, filiform ostioles, elongate asci and filiform, septate ascospores. The genus initially accommodated *O. bacillatum* (Cooke) Sacc. *O. dolichostomum*, *O. friesii* (Mont.) Sacc., *O. hystrix* (Ces.) Sacc., *O. macrocarpum* (Sacc.) Sacc., *O. longisporum* Sacc. and *O. therryanum* (Sacc. & Roum.) Sacc [7]. *Ophioceras* was accommodated in Magnaporthaceae based on limited molecular data [8,9,10]. However, ~~*Ophioceras*, however,~~ the genus was excluded from Magnaporthaceae and accommodated in ~~a~~ the separate family Ophioceraceae in Magnaporthales based on combined LSU and RPB1 phylogenetic analyses [1]. To date, the asexual morph of *Ophioceras* has not yet been reported [1,2,6], and only 40 epithets are listed under this genus in Index Fungorum [11]. However, only e~~However, *O. bacillatum* is currently treated as *Daruvedia bacillata* (Cooke) Dennis [12]. E~~leven *Ophioceras* species ~~of *Ophioceras*~~ have molecular data in GenBank, ~~and in addition~~and only ~~, the~~ LSU and ITS sequence data are ~~mostly~~ available ~~to the majority of the species of *Ophiocera*~~for these species ~~for this genus~~ [13].

Ophioceras species are commonly discovered from wood in freshwater and are generally clumped under the name *Ophioceras* sp. [40,41], *O. commune* [44,45,46] or *O. dolichostomum* [51,52,53,54]. Twenty-three *Ophioceras* species are listed in Species Fungorum [55] and are accepted in Hyde et al. [3]. *Ophioceras* species occur in disparate streams in different islands and are therefore likely to have separated for millions of years ago, potentially explaining their evolution into distinct species. The number of existing *Ophioceras* species is therefore likely to exceed more than presently known. Hyde et al. [56] has shown that numerous new taxa await description across most under-collected and -studied.

Formatted: Font: Not Italic

Formatted: Font: Not Italic

Commented [JHB13]: Reviewer#2 comment: Is this relevant for the discussion of the results presented in this MS?

Commented [JHB14R13]: We have removed the sentence.

Formatted: Font: Not Italic

Commented [JHB15]: Reviewer#2 comment: what does it mean? LSU and ITS are the regions with greater number of sequences in the genus? Or that LSU and ITS are available to the majority of the species of the genus. I suggest rewriting the sentence.

Commented [JHB16R15]: We would like to inform that mostly LSU and ITS sequence data are available for eleven species of the genus. We have rewritten the sentence.

Commented [JHB17]: Reviewer#2 comment: I believe that the information in this paragraph would be better in the introduction. It could reinforce the problematic and gap of knowledge that this manuscript aims to shed light.

Commented [JHB18R17]: We have removed this paragraph from the discussion and stated it in the introduction following the reviewer suggestion.

Commented [JHB19]: Reviewer#2 comment: Is this the correct word?

Commented [JHB20R19]: We have changed "estimated" to "are accepted".

Formatted: Not Highlight

Formatted: Not Highlight

Formatted: Not Highlight

Huhndorf et al. [8] introduced *Ceratosphaerella castillensis* (≡ *Ceratosphaeria castillensis*) when the new genus *Ceratosphaerella* was introduced, and this species was designated as the generic type. Huhndorf et al. [8] examined the isotype of *Ceratosphaeria castillensis* and compared it with the morphology of their collection (SMH1865) and confirmed the conspecific status based only on morphological characteristics. Molecular data of *C. castillensis* was obtained from Huhndorf's collection (SMH1865), but the type specimen of *C. castillensis* has not yet been sequenced.

Ceratosphaerella comprises *C. castillensis* (C.L. Sm.) Huhndorf, Greif, Mugambi & A.N. Mill. and *C. rhizomorpha* Huhndorf & Mugambi, introduced by Huhndorf et al. [8]. Phylogenetic analyses of the LSU and SSU sequence datasets showed that *Ceratosphaerella* grouped with *Ophioceras* in Magnaporthaceae [8]. Klaubauf et al. [1] only accommodated only *Ophioceras* into Ophioceraceae, but they did not incorporate with *Ceratosphaerella* in their analyses. Thus, *Ceratosphaerella* has remaineds in Magnaporthaceae. To date, there are only two species included in *Ceratosphaerella* [11], and these taxa only have LSU sequence data in GenBank [13]. *Ceratosphaerella castillensis* has been reported only as a sexual morph, while *C. rhizomorpha* is holomorphic and has a didymobotryumDidymobotryum-like asexual morph [8]. In Luo et al. [2], *Ceratosphaerella* did not group within Magnaporthaceae but clustered with *Ophioceras* in Ophioceraceae. However, Luo et al. [2] did not verify the phylogenetic status of *Ceratosphaerella* in Ophioceraceae, leading to the uncertain placement of the genus.clustered with *Ophioceras* and *Mycoleptodiscus* formed a clade separated from the other families in Magnaporthales. Currently, *Mycoleptodiscus* has been placed in Muyocopronaceae (Muyocopronales) [14]. While, the placement of *Ceratosphaerella* is uncertain.

Formatted: Font: Italic

Formatted: Font: Italic

Formatted: Font: Italic

Formatted: Font: Italic

Formatted: Font: Italic

Formatted: Font: Italic

Commented [MOU21]: Reviewer#2: Are you questioning one of your main results? "Hence, molecular data from the epitype of *C. castillensis* is required to confirm the conspecificity of the type specimen and Huhndorf's collection (SMH1865) as well as its phylogenetic position in *Ophioceras*"

Commented [MOU22R21]: In this sentence, we would like to discuss that even though *C. castillensis* is synonymized under *Ophioceras* based on current phylogenetic analyses in current study. But as mentioned above that the type specimen has not yet been sequenced. Therefore, this will bring an open up question how to make sure that this species is real *C. castillensis* as it is not the extype stain. Therefore, the epitype of *C. castillensis* should be designated for confirm and support our result. However, we remove this sentence in introduction part and rewritten the discussion

Commented [MOU23R21]:

Formatted: Not Highlight

Commented [JHB24]: Reviewer#2 comment: Is this relevant for the discussion here? If it is, maybe a previous context about this genus should be presented.

Commented [JHB25R24]: We have removed the sentence and rewritten.

This study aims to introduce the novel taxon, *Ophioceras sichuanense* sp. nov., as a saprobe on a submerged bamboo branch in Sichuan Province, China based on morphological characteristics and multi-locus phylogeny while also resolving the congeneric status of *Ceratosphaerella* and *Ophioceras* in Ophioceraceae based on a morpho-molecular approach. *Ceratosphaerella* is synonymized under *Ophioceras*, and two species combinations are introduced, including an updated generic description, illustration, and multi-locus phylogeny.

~~In the present study, we plan to illustrate the taxonomic placement of *Ceratosphaerella* based on morphological characteristics and phylogenetic evidence. In addition, *Ophioceras* is evaluated and compared with similar genera in Magnaporthales, meanwhile, *Ophioceras sichuanense* sp. nov. is introduced and illustrated based on morphological comparisons to similar *Ophioceras* species and Magnaporthales phylogenetic analyses of LSU-RPB1-ITS sequences matrix.~~ ~~In this study, *Ceratosphaerella* is synonymized under *Ophioceras* with evidence from morphological characteristics and phylogenetic evidence. Hence, two new combinations are introduced viz. *Ophioceras castillensis* (C.L. Sm.) H.B. Jiang, Phookamsak & K.D. Hyde comb. nov. and *O. rhizomorpha* (Huhndorf & Mugambi) H.B. Jiang, Phookamsak & K.D. Hyde comb. nov. A novel taxon, *O. sichuanense* sp. nov. is also introduced. *Ophioceras* is compared with similar genera in Magnaporthales.~~

Commented [MOU26]: Reviewer#2 comment: suggest not revealing all the results of the MS here.
I would be better presenting it as the aims of the study.
For ex:
Test the phylogenetic relationship of Ceratosphaerella (or something like that); and something related to the study/identification of the sp nov.

Commented [MOU27R26]: We have rewritten this paragraph.

Formatted: Not Highlight

Formatted: Not Highlight

Formatted: Not Highlight

Formatted: Not Highlight

Formatted: Not Highlight

Formatted: Not Highlight

Commented [JHB28]: Reviewer#2 comment: I suggest not revealing all the results of the MS here. I would be better presenting it as the aims of the study. For ex: Test the phylogenetic relationship of *Ceratosphaerella* (or something like that); and something related to the study/identification of the sp. nov.

Commented [JHB29R28]: Yes, we agree it. We have modified the paragraph.

Materials and methods

Sample collection, observation and isolation

Decaying branches of bamboo submerged in freshwater were collected in the stream in the Shunan Artificial Bamboo Forest~~Submerged decaying branches of bamboo were collected near~~

~~the stream in Shunan artificial bamboo forest~~, Sichuan Province, P.R. China in July 2019. ~~The~~ ~~s~~Samples were kept in a paper bag for further morphological examination in the laboratory. Pseudostromata~~Fungal fruiting bodies~~ visualized on decaying branches of bamboo were observed and examined under ~~the~~ a stereo microscope (Motic series SMZ 140) and captured ~~by~~ via ~~the~~ digital phone camera (iPhone 7, Apple Inc., ~~The~~ USA). Microscopic features (e.g., asci, ascospores and paraphyses) were prepared using the~~by~~ squashing mount technique in sterilized distilled water on clean slides for ~~the~~ morphological study. Sections of pseudostromatic ascomata, ostiolar necks and peridial structures were ~~carried out~~ by ~~free~~ free-hand sectioning using Gillette razor blades. Melzer's reagent and Indian ink were used to detect the J-/J+ apical ring of the asci and ~~the~~ mucilaginous sheath ~~surrounded~~ surrounding the ascospores, respectively. Morphological features visualized under a Nikon ECLIPSE Ni compound microscope~~,~~ were photographed ~~by~~ using a Canon EOS 600D digital camera. Measurements (n = 10–20) of pseudostromata, locules, peridium, paraphyses, asci and ascospores were carried out ~~by~~ using Tarosoft (R) Image Frame Work version 0.9.7. Photographic plate and line drawing of fungal morphologies were edited and combined ~~by~~ using Adobe Photoshop CS6 ~~program~~ (Adobe Systems Inc., ~~The~~ USA).

~~The s~~Single spore isolation based on the spore suspension technique [15] was carried out to obtain a pure fungal culture. Germinated ascospores were transferred to the new potato dextrose agar plates (PDA; Qingdao Daily Water Biotechnology co. LTD. Shandong, P.R. China) under aseptic conditions and grown under normal day/night~~–~~light at room temperature. Culture characteristics (e.g., growth, shape, colour, margin, elevation, consistency) were checked and recorded after one week and four weeks.

The holotypic specimen ~~of *Ophioceras sichuanense*~~ is conserved in the herbarium of Cryptogams Kunming Institute of Botany Academia Sinica (KUN-HKAS), Yunnan, P.R. China.

Commented [MOU30]: Reviewer#2 comment: Ascomata

Commented [MOU31R30]: We have changed it to "Pseudostromata" as our fungus form pseudostromata.

Commented [JHB32]: Reviewer#2 comment: Ascomata

Commented [JHB33R32]:

Commented [JHB34]: Reviewer comment: number of structures measured per specimen?

Commented [JHB35R34]: we have added (n = 10-20) it means that we measure 10-20 per structure.

Commented [JHB36]: Reviewer comment: I would not mention the name of new sp. here.

Commented [JHB37R36]: We have deleted.

The isotype is stored in the herbarium of Mae Fah Luang University, Chiang Rai, Thailand (MFLU). Ex-type living cultures are preserved ~~at~~ in the Kunming Institute of Botany Culture Collection (KUMCC) and Mae Fah Luang University Culture Collection (MFLUCC). Facesoffungi and Index Fungorum numbers were registered for the new taxon [11,16].

DNA extraction, amplification and sequencing

Fungal genomic DNA was extracted from fresh mycelia ~~by~~ using the Biospin Fungus Genomic DNA Extraction Kit (BioFlux®, P.R. China) following manufacturer's instructions (Hangzhou, P.R. China) and also extracted from fruiting bodies (= pseudostromata) directly ~~by~~ using the Forensic DNA Kit (Omega®, USA) for a duplicated strain. DNA amplification was performed by polymerase chain reaction (PCR). Four gene regions~~: –~~ including the internal transcribed spacer (ITS), ~~the~~ 28S large subunit rDNA (LSU), ~~the~~ 18S small subunit rDNA (SSU)~~,~~ and ~~the~~ translation elongation factor 1-alpha (TEF1-α) were used to amplify ~~the~~ PCR fragments using forward and reward primer pairs: ITS5/ITS4 [17], LR0R/LR5 [18], NS1/NS4 [17], EF1-983F/EF1-2218R [19], respectively. PCR reactions were conducted in a 25 μL total volume, consisted of 2 μl of DNA template, 1 μl of each forward and reverse primer, 12.5 μl of 2× Power Taq PCR Master Mix (Beijing BioTeke Corporation, P.R. China) and 8.5 μl double-distilled water (ddH_2O). The PCR thermal cycle program for ITS, LSU, SSU and TEF1-α gene was set up following Jiang et al. [20]. PCR fragments were purified and sequenced at TsingKe Biological Technology (Beijing) Co., Ltd, P.R. China.

Molecular phylogeny

The newly generated sequences (ITS, LSU and SSU) of fungal strains were initially subjected to the basic local alignment search tool (BLASTn) via the National Center for Biotechnology Information web portal (NCBI; https://blast.ncbi.nlm.nih.gov) for discovering the closely related fungal taxa. In order to clarify the phylogenetic placement of the new taxonisolate, the representative taxa in Magnaporthales were then incorporated with the new taxon isolate to perform the sequence data matrix for further analysis. These representative taxa of Magnaporthales were downloaded from the GenBank database (Table 1) based on recent publications [2,14].

Preliminary single-gene data matrixes were aligned via MAFFT v. 7.452 [21] and improved manually in BioEdit v. 5.0.6 [22]. The single-gene alignments of LSU and ITS data matrixes were prior analyzed by maximum-likelihood (ML) criterion using RAxML v. 7.0.3 [23, 24] for checking if there are any conflicts between the tree topologies. The concatenated LSU-ITS–, and LSU-RPB1-ITS sequence datasets were further analyzed based on maximum-likelihood (ML) and Bayesian inference (BI) criteria and the tree topologies of these combined gene datasets were compared for checking the congruence of the tree topologies.were further analyzed based on maximum-likelihood (ML) criteria and the generating tree topology was The concatenated LSU-ITS and LSU-RPB1-ITS sequence datasets comprise 64 strains of ingroup taxa in Magnaporthales. *Myrmecridium schulzeri* (CBS 100.54) and *M. sorbicola* (CBS 143433) were selected as the outgroup taxa. It is notable that the SSU and TEF1-α loci are not included in the present analyses due to fewer available sequences of SSU and TEF1-α loci in our complied dataset, and thus, these two loci were excluded to avoid missing data or gaps which may affect tree topology.compared withand topologies from LSU-RPB1-ITS sequence datasets were

Commented [MOU38]: Reviewer#2 comment: It is a result "Based on the results from BLASTn search tool of LSU and SSU sequences, our new isolate is closest with species of *Ophioceras*, whereas, ITS sequence revealed that our new isolate matches with the unidentified fungal endophyte isolate 4583 (86.79% similarity)."

Commented [MOU39R38]: We have moved it to the results part.

Commented [JHB40]: Reviewer comment: consider avoid "reveal" the new taxon here

Commented [JHB41R40]: We have changed "new taxon" to "new isolate" here.

Commented [JHB42]: Reviewer comment: what about TEF?

Commented [JHB43R42]: 29 strains of our dataset (66 strains) have TEF seq data. TEF seqs are not enough and generating topology cannot be compared with other single gene topology. Therefore, in this study, we did not include TEF in our phylogenetic analyses.

Formatted: Not Highlight

Commented [JHB44]: Reviewer comment: what about TEF and SSU?

Commented [JHB45R44]: 29 strains of our alignment (66 strains) have TEF seq data and 25 strains of our alignment (66 strains) have SSU seq data. The TEF and SSU seq data are severely not enough and respective generating topologies cannot be compared ...

Formatted: Not Highlight

Commented [MOU46]: Reviewer#2 comment: What do ...

Commented [MOU47R46]: We have rewritten the ...

Formatted: Not Highlight

Commented [MOU48]: Reviewer#2 comment: method

Commented [MOU49R48]: We have moved this ...

Formatted: Not Highlight

Formatted: Not Highlight

Formatted: Not Highlight

Formatted: Not Highlight

Formatted: Not Highlight

~~further analyzed based on maximum likelihood (ML) and Bayesian inference (BI) criteria for comparing the tree topologies.~~

Maximum-likelihood (ML) criterion was analyzed by the online tool RAxML-HPC v.8 on XSEDE (8.2.12) via CIPRES Science Gateway v. 3.3 web portal [25]. The ML + thorough bootstrap parameters were set~~up~~ at default values but modified as 1000 replications of bootstraps (-N 1000) and using the GTRGAMMA model.

The best-fit evolutionary models of nucleotide substitution for LSU, RPB1 and ITS loci were evaluated by MrModeltest 2.3 [26], and the GTR+I+G substitution model under the Akaike Information Criterion (AIC) was the best-fit evolutionary models for each locus. Bayesian inference (BI) analysis was performed by MrBayes v. 3.1.2 [27]. The Markov Chain Monte Carlo sampling (MCMC) sampling method was used to determine ~~the~~ posterior probabilities (PP) [28,29].

One million generations of six simultaneous Markov chains were run and sampled every 100th generation. MCMC heated chain was set up with a “temperature” value at 0.15. The burn-in was set to 20% of all sampled trees, meaning that ~~that means~~ sampled trees beneath the asymptote (20%) were discarded. ~~The p~~Posterior probabilities values were then calculated from the remaining 8000 trees in the majority rule consensus tree.

The final phylogram presented in this study was visualized in FigTree v. 1.4.0 (http://tree.bio.ed.ac.uk/software/figtree/). The phylogenetic tree was edited ~~by~~ in Microsoft Office PowerPoint 2016 (Microsoft Inc., ~~The~~ USA) and converted to jpeg file ~~by~~ using Adobe Photoshop CS6 ~~program~~ (Adobe Systems Inc., ~~The~~ USA). New sequences generated from the present study were registered for ~~the~~ GenBank accession numbers (Table 1). The final alignment and phylogram are submitted in TreeBASE (https://www.treebase.org/, submission ID: 26989).

Commented [JHB50]: Reviewer comment: what do you mean with “comparing”?

Commented [JHB51R50]: The sentence has some problem. We have rewritten it.

Commented [JHB52]: Reviewer comment: what about TEF and SSU?

Commented [JHB53R52]: 29 strains of our alignment (66 strains) have TEF seq data and 25 strains of our alignment (66 strains) have SSU seq data. The TEF and SSU seq data are severely not enough and respective generating topologies cannot be compared with each other and other topologies.

Formatted: Font color: Auto

Commented [JHB54]: Reviewer comment: result?

Commented [JHB55R54]: We have stated the results evolution model after test here for further analyze BI. This is a preliminary result for further phylogenetic analyses. Therefore, we did not state it in the results part.

Formatted: Indent: First line: 2 ch, Widow/Orphan control

Table 1. Detailed information of fungal taxa used in ~~the present~~ the phylogenetic analyses ~~incorporated with the GenBank accession numbers~~. The newly generated sequences are indicated in **blue**, and the ex-type strains are in **bold**.

Commented [JHB56]: Reviewer comment: where are SSU and TEF?

Commented [JHB57R56]: This table is included final seq data that we used to infer the phylogenetic tree. Therefore, SSU and TEF are not included in this table.

Species name	Culture collection/ Voucher no.	GenBank accession numbers		
		LSU	RPB1	ITS
Aquafiliformis lignicola	**MFLUCC 16-1341**	MK835815	/	MK828615
Aquafiliformis lignicola	MFLUCC 18-1338	MK835814	/	MK828614
Bambusicularia brunnea	**INA-B-92-45**	NG_058671	KM485043	NR_145387
Barretomyces calatheae	CBS 129274	MH876639	KM485045	MH865202
Bifusisporella sorghi	**URM 7442**	NG_067852	MK060159	NR_164042
Budhanggurabania cynodonticola	**BRIP 59305**	NG_058678	KP162143	NR_137952
Buergenerula spartinae	ATCC 22848	DQ341492	JX134720	JX134666
Bussabanomyces longisporus	**CBS 125232**	NG_058668	KM485046	NR_145385
Ceratosphaeria aquatica	**MFLU 18-2323**	MK835812	/	MK828612
Ceratosphaeria lampadophora	SMH 4822	AY346270	/	/
Ceratosphaeria lignicola	**MFLU 18-1457**	MK835813	/	MK828613
Deightoniella roumeguerei	CBS 128780	MH876533	KM485047	MH865092
Falciphora oryzae	**CBS 125863**	NG_064356	KJ026706	NR_153972
Falciphoriella solaniterrestris	**CBS 117.83**	NG_058108	KM485058	NR_153995
Gaeumannomycella caricis	**CBS 388.81**	NG_058109	KM485059	NR_146245
Gaeumannomyces amomi	CMUZE002	DQ341493	/	AY265318
Gaeumannomyces radicicola	**CBS 296.53**	NG_058089	KM009194	NR_146246
Gaeumannomyces tritici	CBS 541.86	DQ341497	/	/
Kohlmeyeriopsis medullaris	**CBS 117849**	NG_058110	KM485068	NR_154068
Macgarvieomyces borealis	**CBS 461.65**	NG_058088	KM485070	NR_145384
Macgarvieomyces juncicola	CBS 610.82	KM484970	KM485071	KM484855

Species name	Culture collection/ Voucher no.	GenBank accession numbers		
		LSU	RPB1	ITS
Magnaporthiopsis incrustans	M35	JF414892	JF710437	JF414843
Magnaporthiopsis maydis	CBS 133165	KX306614	/	KX306544
Magnaporthiopsis maydis	**CBS 662.82A**	NG_058111	KM485072	NR_154175
Magnaporthiopsis poae	**M47**	JF414885	JF710433	JF414836
Muraeriata africana	**GKM 1084**	EU527995	/	/
Muraeriata collapsa	**SMH 4553**	EU527996	/	/
Myrmecridium schulzeri	CBS 100.54	EU041826	/	EU041769
Myrmecridium sorbicola	**CBS 143433**	NG_063957	/	NR_158871
Nakataea oryzae	CBS 252.34	MH867001	KM485078	KM484862
Nakataea oryzae	CBS 288.52	MH868571	KM485080	MH857040
Neocordana malayensis	**CBS 144604**	NG_066327	/	NR_163364
Neocordana musae	**CPC 18127**	LN713290	/	NR_154265
Neogaeumannomyces bambusicola	**MFLUCC 11-0390**	NG_059556	/	NR_146247
Neopyricularia commelinicola	**CBS 128308**	NG_058112	KM485087	NR_154226
Omnidemptus affinis	**ATCC 200212**	NG_059478	JX134728	NR_154292
Omnidemptus graminis	**CBS 138107**	MK487734	/	NR_164058
Ophioceras aquaticus	**IFRDCC 3091**	JQ797433	/	JQ797440
Ophioceras aquaticus	MFLUCC 16-0906	MK835810	/	MK828611
Ophioceras castillensis (as *Ceratosphaeria castillensis*)	SMH 1865	EU527997	/	/
Ophioceras chiangdaoense	**CMU 26633**	NG_066356	/	/
Ophioceras commune	KUN-HKAS 92569	MH795820	/	MH795815
Ophioceras commune	KUN-HKAS 92587	MH795819	/	MH795814
Ophioceras commune	KUN-HKAS 92590	MK835809	/	MK828610
Ophioceras commune	KUN-HKAS 92640	MH795818	/	MH795813
Ophioceras dolichostomum	CMURp50	DQ341504	/	/

Commented [JHB58]: Reviewer comment: (as Ceratosphaeria castillensis) the same for the other

Commented [JHB59R58]: We have added it."

Formatted: Not Highlight

Species name	Culture collection/ Voucher no.	GenBank accession numbers		
		LSU	RPB1	ITS
Ophioceras hongkongense	HKUCC 3624	DQ341509	/	/
Ophioceras leptosporum	**CBS 894.70**	NG_057959	JX134732	NR_111768
Ophioceras rhizomorpha (as *Ceratosphaerella rhizomorpha*)	**GKM 1262**	EU527998	/	/
Ophioceras sichuanense	KUN-HKAS 107677	MW057782	/	MW057779
Ophioceras sichuanense	**KUMCC 20-0213**	MT995046	/	MT995045
Ophioceras submersum	**MFLUCC 18-0211**	MK835811	/	/
Proxipyricularia zingiberis	**CBS 133594**	NG_063934	KM485091	AB274434
Pseudohalonectria fagicola	**MFLUCC 15-1117**	KX426219	/	/
Pseudohalonectria hampshirensis	**MFLUCC 15-0774**	KX426218	/	/
Pseudohalonectria lignicola	SMH 2440	AY346299	/	/
Pseudohalonectria lutea	CBS 126574	MH875622	/	MH864160
Pseudophialophora eragrostis	**CM12m9**	KF689638	KF689618	NR_146240
Pseudopyricularia cyperi	**CBS 133595**	NG_058113	/	NR_137920
Pseudopyricularia kyllingae	**CBS 133597**	NG_058114	KM485096	NR_155645
Pyricularia ctenantheicola	GR0001	KM484994	KM485098	KM484878
Pyricularia grisea	BR0029	KM484995	KM485100	KM484880
Pyriculariopsis parasitica	HKUCC 5562	DQ341514	/	/
Slopeiomyces cylindrosporus	**CBS 609.75**	KM485040	KM485158	KM484944
Slopeiomyces cylindrosporus	CBS 610.75	NG_057751	JX134721	NR_120170
Xenopyricularia zizaniicola	CBS 132356	KM485042	KM485160	KM484946

Abbreviations: **ATCC**: American Type Culture Collection, Virginia, USA; **CBS**: Westerdijk Fungal Biodiversity Institute, Utrecht, Netherlands; **CMU**: Chiang Mai University, Chiang Mai, Thailand; **CPC**: Culture Collection of Pedro Crous, Netherlands; **KUN-HKAS**: the Herbarium of Cryptogams Kunming Institute of Botany Academia Sinica, Yunnan, P.R. China; **HKUCC**: Hong Kong University Culture Collection, Hong Kong, P.R. China; **IFRDCC**: Fungal Research

& Development Centre Culture Collection, P.R. China; **KUMCC**: Kunming Culture Collection, Yunnan, P.R. China; **MFLU**: the Herbarium of Mae Fah Luang University, Chiang Rai, Thailand; **MFLUCC**: Mae Fah Luang University Culture Collection, Chiang Rai, Thailand.

Nomenclature

The electronic version of this article in Portable Document Format (PDF) in a work with an ISSN or ISBN will represent a published work according to the International Code of Nomenclature for algae, fungi, and plants, and hence the new names contained in the electronic publication of a PLOS ONE article are effectively published under that Code from the electronic edition alone, so there is no longer any need to provide printed copies.

Commented [JHB60]: Reviewer comment: Is this necessary?

Commented [JHB61R60]: It is a journal format.

In addition, new names contained in this work have been submitted to Index Fungorum from where they will be made available to the Global Names Index. The unique Index Fungorum number can be resolved and the associated information viewed through any standard web browser by appending the Index Fungorum number contained in this publication to the prefix www.indexfungorum.org/. The online version of this work is archived and available from the following digital repositories: PubMed Central and LOCKSS.

Compliance with Ethical Standards

There is no conflict of interest (financial or non-financial) and all authors have agreed to submission of paper. The authors also declare that they have no conflict of interest and confirm that the field studies did not involve endangered or protected species.

Results

Molecular phylogeny

Based on the results from the nucleotide BLASTn search tool of LSU and SSU sequences, our new isolate isstrains (KUMCC 20-0213 and KUN-HKAS 107677) closest with species ofare closely related to *Ophioceras*, whereas, ITS sequence revealed that our new strains are closely related to the unidentified fungal endophyte isolate 4583 (86.79% similarity) and other taxa in Magnaporthales. The combined LSU, RPB1 and ITS sequence matrix comprise 64 strains of ingroup taxa in Magnaporthales including the new taxon. *Myrmecridium schulzeri* (CBS 100.54) and *M. sorbicola* (CBS 143433) were selected as the outgroup taxa. The concatenated LSU-RPB1-ITS dataset included 2594 total characters with gaps (LSU: 1–905 bp, RPB1: 906–1877 bp, ITS: 1878–2594 bp). The best scoring ML tree with the final ML optimization likelihood value of -23570.931644 (ln) was selected to represent the phylogenetic relationships of taxa in Magnaporthales (Fig. 1). All free model parameters were estimated using theby GTRGAMMA model, with 1354 distinct alignment patterns and 40.89% of undetermined characters or gaps. Estimated base frequencies were as follows: A = 0.247576, C = 0.255381, G = 0.292293, T = 0.204750, with substitution rates AC = 1.508478, AG = 2.781197, AT = 1.835341, CG = 0.983246, CT = 6.427316, GT = 1.000000. The Tree-Length = 7.381586 and the gamma distribution shape parameter α = 0.605644. and tThe evaluation of Bayesian posterior probabilities (BYPP) from MCMC was carried out with the final average standard deviation of split frequencies reached 0.009466.

The tree topology from ML analysis showed similar results with the BI analysis and a comparing LSU-ITS, and LSU-RPB1-ITS phylograms also revealed similarities were also

Formatted: Not Highlight

Commented [JHB62]: Reviewer comment: It is a result

Commented [JHB63R62]: We have moved it to the results part.

Formatted: Not Highlight

Formatted: Not Highlight

Formatted: Not Highlight

Commented [JHB64]: Reviewer comment: method

Commented [JHB65R64]: We refer to an updated phylogeny of Sordariomycetes (Hongsanan et al. 2017) and find Magnaporthales and Myrmecridiales are phylogenetically close and become sister order each other. Therefore, we choose typical taxa in Myrmecridiales as outgroup.

Formatted: Not Highlight

similar in overall topologies (Figs. 1 and S2). Fig). Thus, we are going towill use the LSU-RPB1-ITS topology for further discussion. Five families of Magnaporthales were included in the presented phylogenetic analyses viz. Ceratosphaeriaceae, Magnaporthaceae, Ophioceraceae, Pseudohalonectriaceae and Pyriculariaceae. These five families formed well-resolved monophyletic clades within Magnaporthales with significant support (greater than 70% ML and 0.95 PP) in our combined gene analyses (Figs. 1 and S2). Ophioceraceae has a close relationship with Ceratosphaeriaceae and Pseudohalonectriaceae. However, the phylogenetic relationships of these three families are not well resolved and pending further clarification. The present phylogenetic topology includes all families of Magnaporthales viz. Ceratosphaeriaceae, Magnaporthaceae, Ophioceraceae, Pseudohalonectriaceae and Pyriculariaceae. Phylogenetic analyses of the LSU-RPB1-ITS sequence matrix revealed that the investigated specimen (KUN-HKAS 107677) and its pure culture *Ophioceras sichuanense* (KUMCC 20-0213 and KUN-HKAS 107677) are grouped together and formed an independent lineage basal to *Ophioceras* in Ophioceraceae with high statistical support (100% ML, 1.00 PP; Fig 1).

Phylogenetic analyses of the LSU-RPB1-ITS sequence matrix revealed that the investigated specimen (KUN-HKAS 107677) and its pure culture (KUMCC 20-0213) are grouped together and form an independent lineage basal to *Ophioceras* in Ophioceraceae with high statistical support (100% ML, 1.00 PP; Fig. 1). Considering the phylogenetic results and morphology, we propose a novel species, *Ophioceras sichuanense*, occurring on submerged bamboo in Sichuan Province, China.

Ceratosphaeria castillensis (SMH 1865) formed a robust clade with *C. rhizomorpha* (GKM 1262) (100% ML, 1.00 PP; Fig. 1) within *Ophioceras* (81% ML, 0.91 PP; Fig. 1).

Commented [JHB66]: Reviewer comment: Thus, we are going to use the LSU-RPB1-ITS topology for further discussion.

Commented [JHB67R66]: We have added the sentence.

Commented [MOU68]: Reviewer#2 comment: “Thus, we are going to use the LSU-RPB1-ITS topology for further discussion.”

Commented [MOU69R68]: We have added the sentence

Formatted: Not Highlight

Formatted: Not Highlight

Formatted: Not Highlight

Formatted: Not Highlight

Commented [JHB70]: Reviewer comment: the specimens investigated here

Commented [JHB71R70]: we have revised the sentence to add “the specimens investigated here” ...

Commented [JHB72]: Reviewer comment: In light of ...

Commented [JHB73R72]: We have added this ...

Formatted

Formatted: Not Highlight

Commented [MOU74]: Reviewer#2 comment: “the ...

Commented [MOU75R74]: We have revised the ...

Formatted: Not Highlight

Formatted: Not Highlight

Formatted: Not Highlight

Commented [MOU76]: Reviewer#2 comment: “In light ...

Commented [MOU77R76]: We have revised the phrase

Formatted: Not Highlight

Formatted: Font: Not Italic

Formatted: Not Highlight

Formatted: Not Highlight

Formatted: Not Highlight

Formatted: Not Highlight

Formatted ...

Formatted: Not Highlight

Ceratosphaeria castillensis (SMH 1865) and *C. rhizomorpha* (GKM 1262) clustered with *Ophioceras aquaticus* (IFRDCC 3091, MFLUCC 16-0906), *O. dolichostomum* (CMURp50), *O. hongkongense* (HKUCC 3624), *O. submersum* (MFLUCC 18-0211) and *O. commune* (KUN-HKAS 92569, KUN-HKAS 92587, KUN-HKAS 92590, KUN-HKAS 92640) in our all analyses (Figs. 1, S1 and S2) and separated distantly from taxa in Magnaporthaceae. Thus, *Ceratosphaerella* is treated as a synonym of *Ophioceras*, the prior introduced genus, in Ophioceraceae. In light of the result, we can propose a novel species, *Ophioceras sichuanense*, from phylogeny. *Ceratosphaerella* cluster *Ophioceras* species in Ophioceraceae with moderate statistical support (81% ML, 0.91 PP; Fig 1) and formed a separated clade far away from taxa in Magnaporthaceae. In term of this phylogenetic result, *Ceratosphaerella* should be placed in Ophioceraceae rather than Magnaporthaceaeand is grouped with other *Ophioceras* species in Ophioceraceae with moderate statistical support (81% ML, 0.91 PP; Fig 1). This phylogenetic result is in agreements with other previous studies by Huhndorf et al. [8], Perera et al. [30] and Luo et al. [2]. However, these authors did not give an explanation on the phylogenetic results for *Ceratosphaerella*.

Fig 1. Maximum likelihood tree revealed by RAxML analysisPhylogram of RAxML analysis based on a combined LSU, RPB1 and ITS sequence matrix for taxa in Magnaporthales. Bootstrap support values for ML equal to or higher than 70% and the Bayesian posterior probabilities equal to or higher than 0.90 PP are defined above the nodes as ML/PP. The tree is rooted with *Myrmecridium schulzeri* (CBS 100.54) and *M. sorbicola* (CBS 143433). Ex-type strains are in black bold, and new species and new

Formatted: Font: (Default) Times New Roman, 12 pt, Not Highlight

Formatted: Not Highlight

Formatted: Font: (Default) Times New Roman, 12 pt, Not Highlight

Formatted: Not Highlight

Commented [MOU78]: Reviewer#2 comment: distantly related?

Commented [MOU79R78]: We have revised the phrase.

Formatted: Not Highlight

Commented [JHB80]: Reviewer comment: distantly related?

Commented [JHB81R80]: The sentence means *Ceratosphaerella* should be positioned at Ophioceraceae rather than Magnaporthaceae. This is not related to distance. This result is based on present ...

Formatted ...

Commented [JHB82]: Reviewer comment: It should be ...

Commented [JHB83R82]: The sentence has been ...

Formatted ...

Commented [JHB84]: Reviewer comment: discussion

Commented [JHB85R84]:

Formatted ...

Commented [JHB86]: Reviewer comment: I strongly ...

Commented [JHB87R86]: We have modified it.

Commented [MOU88]:

Formatted ...

Formatted: Font: 12 pt

Commented [MOU89]: Reviewer#2 comment: I strongly ...

Commented [MOU90R89]: We have revised the ...

Formatted: Font: Bold, Font color: Black

Formatted: Justified

Formatted: Not Highlight

Commented [JHB91]: Reviewer#2 comment: Maximum ...

Commented [JHB92R91]: We have changed it to ...

combinations are indicated in ~~blue~~red bold.

Taxonomy

Ophioceraceae Klaubauf, Lebrun & Crous, Studies in mycology 79: 103 [1]~~ emend.~~

~~[urn:lsid:indexfungorum.org:names:810201]~~

~~Facesoffungi number: FoF01254~~

~~*Saprobic* on woody and herbaceous plants in aquatic or terrestrial environments. **Sexual morph**: *Ascomata* black, perithecial, immersed to superficial, scattered or gregarious, globose to subglobose, or ampulliform, glabrous, with ostiolar necks, somewhat forming uni- to multi-loculate pseudostromata. *Pseudostromata if present*: locules immersed in pseudostroma, dark brown to black, subglobose to ampulliform, or irregular in shape, with a long, cylindrical, black, brittle, curved or straight, periphysate neck. *Peridium* composed of several layers, of pseudoparenchymatous cells, arranged in a *textura angularis*, inner layers composed of hyaline, elongated cells, with compressed, heavily pigmented cells towards the outer layers. *Paraphyses* filiform, hyaline, unbranched, septate, broad at the base, tapering towards the tip. *Asci* unitunicate, 8-spored, subcylindrical to acerose, or clavate, unitunicate, pedicellate or sessile, with a refractive J-, apical ring. *Ascospores* filiform or narrow fusiform, with rounded ends, slightly curved or sigmoidal, hyaline, olivaceous or pale brown, aseptate or septate, with or without guttulate, lacking sheath. **Asexual morph**: Hyphomycetous, didymobotryum-like (as *Ceratosphaerella rhizomorpha* [8]). *Colony on substrates* dark brown to black, rhizomorphic-like threads, radiated from central of clustered ascomata on a patched subiculum. *Synnemata* formed on rhizomorphic strands, dichotomously branched hyphae, straight or flexuous, branched, lighter brown head, black in mass, with conidiophores at the apical region.~~

Commented [JHB93]: What is new in this description? Is it necessary to present the full description of a known taxon. Does the features of the combined species change the circumscription of the group?

Commented [JHB94R93]: We have deleted the family description

~~*Conidiophores* elongate, septate, with darker bands at the septa, verrucose. *Conidiogenous cells* pale brown, cylindrical, tretic, integrated, terminal, verrucose. *Conidia* pale brown, with darker brown at the septa, ellipsoid to cylindrical, septate, verrucose [8].~~

Type genus: *Ophioceras* Sacc.

Notes – To date, Ophioceraceae includes a single genus, *Ophioceras*. In the present phylogenetic study, Ophioceraceae formed a stable clade within Magnaporthales and distinguished from other families of Magnaporthales.

Ophioceras Sacc., Sylloge Fungorum 2: 358 [7] emend. H.B. Jiang & Phookamsak

[urn:lsid:indexfungorum.org:names:3595]

Facesoffungi number: FoF01255

Synonym: *Ceratosphaerella* Huhndorf, Greif, Mugambi & A.N. Mill., Mycologia 100(6): 941 [8].

Type species: *Ophioceras dolichostomum* (Berk. & M.A. Curtis) Sacc.

Saprobic on bamboo, palm, bark or wood, and other herbaceous plants from aquatic or terrestrial environments. **Sexual morph**: *Ascomata* black, perithecial, immersed to superficial, scattered or gregarious, globose to subglobose, or ampulliform, glabrous with ostiolar necks, somewhat forming uni- to multi-loculate pseudostromata. *Pseudostromata if present*: locules immersed in pseudostroma, dark brown to black, subglobose to ampulliform, or irregular in shape, with a long, cylindrical, black, brittle, curved or straight, periphysate neck. *Peridium* composed of several layers, of pseudoparenchymatous cells, arranged in *textura angularis*, inner layers composed of hyaline, elongate cells, with compressed, dark brown to black cells towards the outer layers. *Paraphyses* filiform, hyaline, unbranched, septate, broad at the base, tapering at

Commented [JHB95]: Reviewer#2 comment: same

Commented [JHB96R95]: We have emended the generic description.

Formatted: Not Highlight

the tip. *Asci* unitunicate, 8-spored, subcylindrical to acerose or clavate, pedicellate or sessile, with a refractive J-, apical ring. *Ascospores* filiform or narrow fusiform, with rounded ends, slightly curved or sigmoidal, hyaline, pale brown or olivaceous, aseptate or septate, with or without guttulate, lacking a sheath. **Asexual morph**: Hyphomycetous, ~~didymobotryum~~Didymobotryum-like. *Colony on substrates* dark brown to black, rhizomorphic-like threads, radiated from central of clustered ascomata on patched subiculum. *Synnemata* formed on rhizomorphic strands, dichotomously branched hyphae, straight or flexuous, branched, lighter brown head, black in mass, with conidiophores at the apical region. *Conidiophores* elongate, septate, with dark brown bands at the septa, verrucose. *Conidiogenous cells* pale brown, cylindrical, tretic, integrated, terminal, verrucose. *Conidia* pale brown, with darker brown at the septa, ellipsoid to cylindrical, 1–3 transverse septa, verrucose [8].

Notes – *Ophioceras* occurs on a wide range of hosts mainly distributed in America, Asia, Africa and Oceania [2,5,8,9,12,31,32,33,34,35,36,37,38,39,40,41,42,43,44,45,46,47,48,49]. To date, only ~~*Ophioceras*~~ *O.* *bambusae* and *O. guttulatum* have been reported from bamboo [47,49,50]. Eleven *Ophioceras* species have been reported from freshwater [2,10], of which~~,~~ nine species were found in China [10,32,46,49]. In this study, *O. sichuanense* is introduced as the second species ~~occurred~~ occurring on submerged decaying branches of bamboo in China.

Ceratosphaerella is morphologically different from *Ophioceras* in having clavate asci and hyaline to pale brown, narrow fusiform ascospores, whereas *Ophioceras* has subcylindrical to acerose asci and hyaline to olivaceous, filiform ascospores [1,8]. However, *Ophioceras* resembles *Ceratosphaerella* in the ascomatal morphology and is also supported by phylogenetic analyses. Through ML and BI phylogenetic analyses based on a concatenated LSU-RPB1-ITS sequence matrix (Fig. 1), *C. castillensis* (SMH 1865), which was previously treated in

Commented [MOU97]: Reviewe#2r comment: I suggest to move the phylogenetic "comments" to the discussion.

Commented [MOU98R97]: We have also discussed phylogenetic result of *Ceratosphaerella* in the discussion.

Formatted: Not Highlight

Formatted: Not Highlight

Formatted: Not Highlight

Formatted: Not Highlight

Magnaporthaceae, is phylogenetically closely related to *C. rhizomorpha* and clustered within *Ophioceras* in Ophioceraceae. ~~*Ceratosphaerella* clustered with *Ophioceras* in Ophioceraceae which is separated from Magnaporthaceae (Fig 1).~~ Therefore, ~~here we propose~~we treat *Ceratosphaerella* ~~is~~as ~~likely to be~~ a synonym of *Ophioceras* instead of a genus in Ophioceraceae. ~~Through ML and BI phylogenetic analyses based on a concatenated LSU-RPB1-ITS sequence matrix, *Ceratosphaerella castillensis* (SMH 1865) which was previously treated in Magnaporthaceae, is phylogenetically close to *C. rhizomorpha* and clustered with~~in *Ophioceras* ~~in Ophioceraceae. Therefore, we transfer~~ Using a morpho-phylogenetic approach, *Ophioceras castillensis* comb. nov. and *O. rhizomorpha* comb. nov. are hereby introduced.~~*Ceratosphaerella castillensis* and *C. rhizomorpha* to *Ophioceras* as *Ophioceras castillensis* comb. nov. and *O. rhizomorpha* comb. nov.~~

Ophioceras sichuanense H.B. Jiang, Phookamsak & K.D. Hyde, *sp. nov.* Fig 2

[urn:lsid:indexfungorum.org:names:557956]

Facesoffungi number: FoF09404

Etymology – The specific epithet "*sichuanense*" refers to Sichuan Province, P.R. China, where the species was collected.

Holotype – KUN-HKAS 107677

Saprobic on decaying branches of bamboo submerged in freshwater. **Sexual morph**: *Pseudostromata* 300–750 µm diam., 230–350 µm high (excluding necks), black, scattered, solitary, semi-immersed to superficial, 1–5-loculate, glabrous, ostiolate, papillate, carbonaceous. *Locules* 150–300 µm diam., 150–250 µm high (excluding necks), immersed within pseudostroma,

Commented [JHB99]: Reviewer comment: you are not sure?

Commented [JHB100R99]: We have changed the word and modified the sentence.

Commented [JHB101]: Reviewer comment: I suggest to move the phylogenetic "comments" to the discussion.

Commented [JHB102R101]: These phylogenetic results are strongly support for our idea (*Ceratosphaerella* is a synonym of *Ophioceras*). Therefore, we think they should be remained in the notes of *Ophioceras*.

Formatted: Font: (Default) Times New Roman

Commented [JHB103]: Reviewer comment: within

Commented [JHB104R103]: We have changed "with" to "within".

Formatted: Font: (Default) Times New Roman

Formatted: Not Highlight

Formatted: Not Highlight

Formatted: Indent: First line: 0.39"

clustered, subglobose to ampulliform, blackened, with a long black, periphysate neck, up to 1 cm. *Peridium* 20–35 µm wide, thick-walled, composed of several layers, of flattened to broad, pseudoparenchymatous cells, arranged in *textura angularis* to *textura prismatica*, inner layers composed of hyaline cells, outer layers composed of dark brown to black pseudoparenchymatous cells. *Paraphyses* 4–8 µm wide, filiform, hyaline, indistinct septate, unbranched, slightly rough with small guttules, broad at the base, tapering toward the tip. *Asci* 90–115 × 5–6.5 µm ($\bar{x}$ = 103 × 5.8 µm, n = 20), 8-spored, unitunicate, cylindrical, sessile to subsessile, with short broad bulb-like at the base, apically rounded with a J-, apical ring. *Ascospores* 80–90 × 1–1.5 µm ($\bar{x}$ = 85 × 1.3 µm, n = 15), overlapping, or in parallel, hyaline, filiform, slightly curved to sigmoidal, thin-walled, aseptate, smooth-walled, multi-guttulate. **Asexual morph**: not observed.

Culture characteristics: Ascospores germinated on PDA within 24 hours at room temperature under normal condition. Mycelium superficial to immersed in agar medium, branched, septate, smooth hyphae. Colonies growing slowly on PDA, reaching 20 mm in two weeks, cottony, circular, with entire edge, raised, white from above and below.

Material examined: P.R. China, Sichuan Province, Yibin City, Shunan ~~artificial~~ Artificial ~~bamboo~~ Bamboo F~~f~~orest, on submerged decaying branches of bamboo, 25 July 2019, H.B. Jiang & R. Phookamsak, SC011 (KUN-HKAS 107677, holotype), ex-type living culture, KUMCC 20-0213.

GenBank accession numbers: ITS = MT995045, LSU = MT995046, SSU = MT995047 (KUMCC 20-0213; from pure culture); ITS = MW057779, LSU = MW057782, SSU = MW057847, TEF1-α = MW082017 (KUN-HKAS 107677; from fruiting bodies).

Notes ~~– *Ophioceras sichuanense* resembles *O. guttulatum* and *O. tenuisporum*. However, the new taxon differs from *O. guttulatum* in smaller asci and ascospores, and multi-loculate~~

Commented [JHB105]: Reviewer#2 comment: what about the phylogenetically related species? What are the differences and resemblances?

Commented [JHB106R105]: As requested from reviewer, we have modified the notes of O. sichuanense.

pseudostromata (Asci: 90–115 × 5–6.5 µm vs. 130–160 × 14–17 µm; Ascospores: 80–90 × 1–1.5 µm vs. 100–128 × 4–5 µm) [46]. *Ophioceras sichuanense* differs from *O. tenuisporum* in having multi-loculate pseudostromata and aseptate ascospores [42]. Although *Ophioceras bambusae* Höhn. and our new taxon share the same host, however, our new taxon has longer asci and ascospores and multi-loculate pseudostromata (Asci: 90–115 × 5–6.5 µm versus 90–95 × 5.5–6.5 µm; Ascospores: 80–90 × 1–1.5 µm versus 75–80 × 1.5 µm) [50].

Ophioceras sichuanense can be distinguished from other *Ophioceras* species because it forms multi-loculate pseudostromata. *Ophioceras sichuanense* resembles *O. guttulatum*, *O. leptosporum* and *O. tenuisporum*. However, *O. sichuanense* differs from *O. guttulatum* in having smaller asci and ascospores (asci: 90–115 × 5–6.5 µm vs. 130–160 × 14–17 µm; ascospores: 80–90 × 1–1.5 µm vs. 100–128 × 4–5 µm) [46]. *Ophioceras sichuanense* resembles *O. leptosporum* and *O. tenuisporum* due to the size ranges of asci and ascospores and extremely long ostiolar necks. However, the species differs from the latter two species in their ascospore septation (Table 2). Although *O. bambusae* and *O. sichuanense* were collected from bamboos, *O. sichuanense* has longer asci and ascospores (asci: 90–115 × 5–6.5 µm vs. 90–95 × 5.5–6.5 µm; ascospores: 80–90 × 1–1.5 µm vs. 75–80 × 1.5 µm) [50]. Present phylogenetic analyses show *O. sichuanense* formed a clade basal to *Ophioceras* and close to *O. chiangdaoense* and *O. leptosporum*. *Ophioceras sichuanense* and *O. chiangdaoense* are different in the dimensions of the ostiolar neck, asci and ascospores (Table 2).

Ophioceras sichuanense resembles *O. guttulatum*, *O. leptosporum* and *O. tenuisporum* from morphology. *Ophioceras sichuanense* is similar to *O. guttulatum* in heavy guttules, however, differs in multi-loculate pseudostromata and smaller dimensions of asci and ascospores (Asci: 90–115 × 5–6.5 µm vs. 130–160 × 14–17 µm; Ascospores: 80–90 × 1–1.5 µm vs. 100–128 × 4–5

Formatted: Not Highlight

Formatted: Not Highlight

Formatted: Not Highlight

Formatted: Not Highlight

Formatted: Not Highlight

Formatted: Not Highlight

Formatted: Not Highlight

Formatted: Not Highlight

Formatted: Not Highlight

Formatted: Not Highlight

Formatted: Not Highlight

Formatted: Not Highlight

Formatted: Not Highlight

Formatted: Not Highlight

Formatted: Not Highlight

Formatted: Not Highlight

Formatted: Not Highlight

~~μm) [46]. *Ophioceras sichuanense* is similar to *O. leptosporum* and *O. tenuisporum* in dimensions of asci and ascospores and extremely long neck, however, differs in multi-loculate pseudostromata and number of septation (Table 2). Although *Ophioceras bambusae* Höhn. and our new taxon share the same host, however, our new taxon has longer asci and ascospores and multi-loculate pseudostromata (Asci: 90–115 × 5–6.5 μm vs. 90–95 × 5.5–6.5 μm; Ascospores: 80–90 × 1–1.5 μm vs. 75–80 × 1.5 μm) [50]. Through the present phylogenetic study, *O. sichuanense* is basal to *Ophioceras* clade and close to *O. chiangdaoense* and *O. leptosporum*. *O. sichuanense* and *O. chiangdaoense* share the morphology of *Ophioceras* (immersed ascomata with long neck, cylindrical asci with a refractive J-, apical ring, and filiform, hyaline ascospores), however, they are totally different in dimensions of neck, asci and ascospores, in addition, *O. sichuanense* owns pseudostromatic structure, but *O. chiangdaoense* lacks it (Table 2).~~

Fig 2. *Ophioceras sichuanense* (KUN-HKAS 107677, holotype). a, b Pseudostromata with long ostiolar necks on host. c–e Section through pseudostromata. f Ascoma. g Ostiole. h Apical part of neck. i Peridium. j–l Asci. m Ascospores. n Paraphyses. Scale bars: d, e = 200 μm, f–h = 50 μm, i = 30 μm, j–n = 20 μm.

Ophioceras castillensis (C.L. Sm.) H.B. Jiang, Phookamsak & K.D. Hyde, *comb. nov.* Fig 3

[urn:lsid:indexfungorum.org:names:557957]

Facesoffungi number: FoF09405

Basionym: *Ceratosphaeria castillensis* C.L. Sm., Bull. Lab. Nat. Hist. Iowa State Univ. 2: 403 (1893)

Synonym: *Ceratosphaerella castillensis* (C.L. Sm.) Huhndorf, Greif, Mugambi & A.N.

Mill., Mycologia 100(6): 944 [8]

Type information: Nicaragua, Castillo Viejo, on bark, Feb–Mar 1893, C.L. Smith, Central American Fungi 13, (isotype, NY).

Detailed description and illustration: see Huhndorf et al. [8].

Known hosts/ habitat and distribution: Saprobic on bark or wood in terrestrial habitat. To date, *Ophioceras castillensis* is only reported from Costa Rica, Nicaragua, and Puerto Rico [8].

Notes – ~~Huhndorf et al. [8] introduced *Ceratosphaerella castillensis* (≡ *Ceratosphaeria castillensis*) when they introduced the new genus *Ceratosphaerella* and also designated this species as the generic type. Huhndorf et al. [8] examined the isotype of *Ceratosphaeria castillensis* compared with the morphology of their collection (SMH1865) and confirmed the conspecificity based only on morphological characters. Molecular data of *C. castillensis* was obtained from Huhndorf's collection (SMH1865). Whereas, the type specimen of *Ceratosphaeria castillensis* has not yet been sequenced. Hence, molecular data from the epitype of *C. castillensis* is required to confirm the conspecificity of the type specimen and Huhndorf's collection (SMH1865) as well as its phylogenetic position in *Ophioceras*. Phylogenetic analysis based on a 18S small subunit rDNA sequence data showed *Ceratosphaerella* formed a close relationship with other *Ophioceras* species within the Magnaporthaceae [8]. However, Huhndorf et al. [8] used the differences of the asci and ascospores as significant characters for distinguishing the genus from *Ophioceras*.~~

Commented [JHB107]: Reviewer#2 comment: I suggest to fucus the notes on the morphological characteristics and the comparisons related to it. The remaining info would be better suited in the discussion.

Commented [JHB108R107]: We have moved the paragraph to the introduction

Commented [JHB109]: Reviewer comment: Are you questioning one of your main results?

Commented [JHB110R109]:

In this study, *Ceratosphaerella castillensis* is transferred to *Ophioceras* as *O. castillensis* based on concatenated LSU-RPB1-ITS analyses coupled with morphological similarity of the ascomata and ascomatal wall which is typical *Ophioceras*. *Ophioceras castillensis* can be separated from other *Ophioceras* species in forming ascomata on large clusters, superficial on

Formatted: No widow/orphan control

Commented [JHB111]: Reviewer comment: Perhaps only with one LSU sequence

Commented [JHB112R111]: We have discussed the phylogenetic status of this species based on our final phylogenetic analyses.

sparse, subicular hyphae~~,~~ and having clavate asci and hyaline to pale brown, fusiform, 3-septate ascospores [8]. Detailed morphological ~~morphologically~~ comparison and ~~the~~ taxa habitats ~~of taxa~~ in *Ophioceras* are described in ~~a synopsis~~ Table 2.

Fig 3. *Ophioceras castillensis* (~~≡ *Ceratosphaeria castillensis*;~~ redrawn from Huhndorf et al. [8], NY isotype). a, b Ascomata. c Ascus. d Apical ring. e Ascospores. f Peridium. g Paraphyses. Scale bars: a = 1 mm, b = 200 µm, f = 20 µm, c, e, g = 10 µm, d = 5 µm.

Ophioceras rhizomorpha (Huhndorf & Mugambi) H.B. Jiang, Phookamsak & K.D. Hyde, *comb. nov.* Fig 4

[urn:lsid:indexfungorum.org:names:557958]

Facesoffungi number: FoF09406

Basionym: *Ceratosphaerella rhizomorpha* Huhndorf & Mugambi, Mycologia 100(6): 944 [8]

Type information: Kenya, Kagamega National Park, on decaying wood on the ground, 17 January 2007, G.K. Mugambi, GKM1262 (holotype EA, isotype F).

Detailed description and illustration: see Huhndorf et al. [8].

Known hosts/ habitat and distribution: Saprobic on decaying wood ~~bark or wood~~ of terrestrial habitat. To date, *Ophioceras rhizomorpha* is only reported from Kenya.

Notes – *Ophioceras rhizomorpha* was reported with a ~~didymobotryum~~Didymobotryum-like asexual morph on ~~the~~ a natural substrate [8]. The species resembles *O. castillensis* in forming ascomata on the large subicular, and having clavate asci and hyaline to pale brown, fusiform,

Commented [JHB113]: Reviewer comment: morphological

Commented [JHB114R113]: We have changed "morphologically" to "morphological"

Commented [JHB115]: Reviewer comment: delete

Commented [JHB116R115]: We have deleted.

3-septate ascospores [8]. *Ophiocera rhizomorpha*, however, differs from *O. castillensis* in having larger ascomata, necks, peridia, paraphyses, asci and ascospores [8] (see Table 2).

Fig 4. *Ophioceras rhizomorpha* (redrawn from Huhndorf et al. [8], all from Mugambi 1262). a Ascomata and synnemata on the substrate. b Ascoma. c Peridium. d Ascus. e Apical ring. f Hyaline to pale brown ascospores. g Paraphyses. h, i Conidiophores. j Conidiogenous cell bearing conidium. k Conidium. Scale bars: a = 1 mm, b = 300 µm, c = 50 µm, g = 30 µm, d = 20 µm, i, k = 15 µm, f, h, j = 10 µm, e = 5 µm.

Discussion

~~*Ophioceras* species have commonly been recovered from wood in freshwater and have generally been clumped under the name *Ophioceras* sp. [40,41], *O. commune* [44,45,46] or *O. dolichostomum* [51,52,53,54]. There are of 23 *Ophioceras* species listed in Species Fungorum [55] and estimated~~ mentioned ~~in Hyde et al. [3]. *Ophioceras* species, occurs in disparate streams in different islands and continents and are therefore likely to have become separated for millions of years and may have evolved into distinct species. The number of species in *Ophioceras* is therefore likely to be more than presently known. Hyde et al. [56] has shown that in most genera that are under collected, numerous new taxa await to be described.~~

Ophioceraceae currently accommodates only *Ophioceras*. Maharachchikumbura et al. [57] accommodated the family in Magnaporthales (Diaporthomycetidae, Sordariomycetes) based on ~~the~~ literature ~~studies~~ review and phylogenetic analysis. The studies of fungal ranking based on molecular clock evidence showed that Ophioceraceae shares a common ancestor with Ceratosphaeriaceae at 65 MYA in Magnaporthales (130–190 MYA) and the subclass

Commented [JHB117]: Reviewer comment: I am not sure if I understood this figure. It is a draw based in the original photographs?
I checked the original article, and the photographs are informative. I mean, what is the input of your figure?

Commented [JHB118R117]: We have provided the line drawing based on the type specimen of *Ophioceras castillensis* here for a more convenient checking on morphological features of the species.

Commented [JHB119]: Reviewer comment: same for fig. 3

Commented [JHB120R119]: We have provided the line drawing based on the type specimen of *Ophioceras rhizomorpha* here for a more convenient checking on morphological features of the species.

Commented [JHB121]: Reviewer comment: I suggest to keep the focus of the discussion in Ophioceraceae

Commented [JHB122R121]:

Commented [JHB123]: Reviewer comment: I believe that the information in this paragraph would be better in the introduction. It could reinforce the problematic and gap of knowledge that this manuscript aims to shed light.

Commented [JHB124R123]:

Commented [JHB125]: Reviewer comment: Is this the correct word?

Commented [JHB126R125]: We have changed "estimated" to "mentioned".

Diaporthomycetidae (247–250 MYA) was the youngest subclass in Sordariomycetes (130–250 MYA) [3,58]. However, the stem age and crown age of the familial ranking are not flexible depending on taxon sampling, gene selection in data matrix, models and parameters selection~~,~~ ~~and~~ as well as the calibration points [58,59].

> **Commented [JHB127]:** Reviewer#2 comment: Interesting information, but what is the relevance of this in relation to your results?

> **Commented [JHB128R127]:** Although this information is not relevant to my results in this study. However, we provided this information as addition for further studies with fungal evolution approach and it will be useful for future taxonomic classification in a higher rank.

Ophioceraceae has limited taxon sampling, and most taxa in this family lack reliable protein coding genes to clarify ~~their~~ phylogenetic affinities. For ~~an~~ example, *Ophioceras arcuatisporum* (strains A9-1, A167-1B) has only SSU sequence data available in GenBank. In p~~P~~reliminary phylogenetic analysis of SSU sequence matrix, the species formed a stable clade within Magnaporthaceae rather than Ophioceraceae. *Ophioceras arcuatisporum* needs to be re-visited and re-illustrated, incorporating details from ~~with detailed of~~ molecular data. ~~Generally, taxa in Magnaporthales lacks SSU sequence data. Hence, SSU gene is not included in our dataset in this study for avoiding undetermined characters or gaps which may affect the tree topology.~~

Luo et al. [2] performed combined LSU and TEF1-α phylogenetic analyses to investigate the relationships of taxa in Magnaporthales. In their phylogeny, *Ceratosphaeria* grouped with *Pseudohalonectria* (Pseudohalonectriaceae) and separated from *Ophioceras* (Ophioceraceae). Based on the fact that *Ceratosphaeria* differs from *Pseudohalonectria* in having narrow cylindric-fusiform to filiform and longer ascospores, Ceratosphaeriaceae was thus introduced as a new family within Magnaporthales to accommodate *Ceratosphaeria* [2]. In the present study, we performed an updated phylogenetic tree based on a concatenated LSU-RPB1-ITS sequences and showed that *Ceratosphaeria* (Ceratosphaeriaceae) clustered with *Ophioceras* (Ophioceraceae) with low statistical support, suggesting that gene selection in the data matrix affects the tree topology ~~of~~ at the familial levels in Magnaporthaceae. *Ceratosphaeria* is morphologically similar to *Ceratosphaerella* [8]. Although~~,~~ *Ceratosphaeria* clustered with

Ophioceras~~,~~ with low statistical support, *Ceratosphaeria* possibly belongs to *Ophioceraceae*. However, the phylogenetic status of *Ceratosphaeria* needs to be clarified with~~proven by~~ more evidence~~s~~ in the future studies.

In the present study, we ~~combine~~ synonymize *Ceratosphaerella* ~~with~~ under *Ophioceras* based on molecular phylogeny coupled with similar ascomatal morphology. ~~*Ophioceras castillensis* (= *Ceratosphaerella castillensis*) and *O. rhizomorpha* (≡ *Ceratosphaerella rhizomorpha*) have only LSU sequence data available in GenBank.~~ P~~reliminary p~~hylogenetic analysis based on ~~a single~~the LSU ~~gene~~ dataset (Fig. S1) and ~~a~~the concatenated LSU-ITS (Fig. S2) and the LSU-RPB1-ITS (Fig. 1) sequence datasets ~~gene region~~ have always shown that *O. castillensis* (= *C. castillensis*) and *O. rhizomorpha* (≡ *C. rhizomorpha*) formed a stable clade within *Ophioceras* ~~(S1 and S2 Figs)~~. These show that *Ceratosphaerella* should be a synonym of *Ophioceras*. ~~Thus, we cannot exclude the possibility that *Ceratosphaerella* is a distinct genus in Ophioceraceae.~~ ~~However, *Ophioceras castillensis* and *O. rhizomorpha* are a little different from other *Ophioceras* species in shape of asci and ascospores (Asci: clavate vs. cylindrical; Ascospores: narrowly fusiform vs. filiform, narrowly fusoid to cylindrical; Table 2). *Ophioceras castillensis* (= *Ceratosphaerella castillensis*) and *O. rhizomorpha* (≡ *Ceratosphaerella rhizomorpha*) have only LSU sequence data available in GenBank.~~ *Ophioceras castillensis*, *O. rhizomorpha* and most *Ophioceras* species lack protein coding genes and other reliable genes to clarify phylogenetic placement as well as limited taxon sampling. The ex-type strain of *O. rhizomorpha* and the reference strain of *O. castillensis* were sequenced only for LSU locus. Hence, more reliable gene loci (e.g., SSU, ITS, RPB1 and TEF1-α) from the ex-type strain of *O. rhizomorpha* should be obtained and the epitype of *O. castillensis* should be designated and incorporated with morpho-molecular based taxonomic treatment. Furthermore, the new

Commented [JHB129]: Reviewer#2 comment: I can not see the difference from the fig 1. In fig. 1 *O. castillensis* and *O. rhizomorpha* are grouped within *Ophioceras*.

Commented [JHB130R129]: We have revised this paragraph.

Commented [JHB131]: Reviewer comment: If it is true, your new species is not an Ophioceras, right?
I don't understand the point here, please clarify.

Commented [JHB132R131]: We have updated this paragraph. Please check it

Formatted: Not Highlight

Formatted: Not Highlight

collections and sequence data of taxa in *Ophioceras* are required to provide a better taxonomic resolution for robust species delineations in this genus.

~~Therefore, new collections and sequence data for *O. castillensis* and *O. rhizomorpha* as well as other *Ophioceras* species are required in order to get a better understanding of *Ophioceras* in further study. New collections and sequence data for *O. castillensis* and *O. rhizomorpha* as well as other *Ophioceras* species are required for a better understanding of this genus.~~

Many genera in Magnaporthales have similar morphological characteristics with *Ophioceras* (Table 3). However, these genera ~~were~~ are considered ~~as~~ distinct genera based on ~~the~~ phylogenetic investigations [1,2,8,60,61,62]. *Pseudohalonectria* (Pseudohalonectriaceae) is also similar to *Ophioceras* in its ascomata and asci. However, *Pseudohalonectria* varied in shape of ascospores, such as ellipsoidal, fusiform or filiform [30]. In the present study, *Pseudohalonectria* (Pseudohalonectriaceae) formed an independent lineage separate from other families in Magnaporthales; however, *Pseudohalonectria* could not be resolved ~~in its~~at the species level such as in ~~agreement of~~ Perera et al. [30]. It may be because ~~of that there are only 14 species available in Species Fungorum [55] and the~~ molecular data of most taxa in this genus ~~is~~ are unavailable in GenBank database. Moreover,~~Also,~~ some sequences of *P. lignicola* deposited in GenBank are likely to be misidentified [30]. Therefore, sequences of *Pseudohalonectria* species used for phylogenetic analyses are limited. ~~The new collections of *Pseudohalonectria* species are needed to obtain molecular data for solving the affinities within the genus.~~

Author Contributions

Conceptualization: Hong-Bo Jiang, Rungtiwa Phookamsak, Kevin D. Hyde, Saisamorn Lumyong.

Commented [JHB133]: Reviewer comment: delete

Commented [JHB134R133]: We have deleted.

Commented [JHB135]: Reviewer comment: There is a lot of uncertainty like this in your discussion. I would not enter in this field (not directly relataded to your problem) with vague information.

Commented [JHB136R135]: We have removed the sentence. However, we have provided the discussion that it is not related to our aims because we would like to provide more information of the genera in our target family and related families in the same order, the taxonomic problem and future work for the genera in this group..

Data curation: Hong-Bo Jiang.

Formal analysis: Hong-Bo Jiang, Rungtiwa Phookamsak.

Funding acquisition: Ali H. Bahkali, Abdallah M. Elgorban, Samantha C. Karunarathna, Rungtiwa Phookamsak, Saisamorn Lumyong.

Investigation: Hong-Bo Jiang, Rungtiwa Phookamsak, Er-Fu Yang, Kevin D. Hyde.

Methodology: Hong-Bo Jiang, Rungtiwa Phookamsak.

Project administration: Rungtiwa Phookamsak.

Supervision: Kevin D. Hyde, Saisamorn Lumyong, Rungtiwa Phookamsak, Pattana Kakumyan.

Writing – original draft: Hong-Bo Jiang, Er-Fu Yang, Rungtiwa Phookamsak.

Writing – review & editing: Kevin D. Hyde, Samantha C. Karunarathna, Pattana Kakumyan, Rungtiwa Phookamsak, Saisamorn Lumyong.

Acknowledgements

The authors gracefully thank the Biology Experimental Center, Germplasm Bank of Wild Species, Kunming Institute of Botany, Chinese Academy of Sciences for providing the facilities of molecular laboratory. We also thank Shaun Pennycook from Manaaki Whenua - Landcare Research for his assistance in naming the new species in the genus *Ophioceras*. Austin G. Smith is thanked the English proofreading of this manuscript Hong-Bo Jiang would like to thank Mae Fah Luang University for Ph.D scholarship.

Table 2. Synopsis of *Ophioceras* species.

**Species name**	**Ascomata**	**Asci**	**Ascospores**	**Habitat**	**Origin**	**Host/substrate~~Substrate~~**	**References**
Ophioceras	310–620 µm	85–100	42–68 × 3–4	Submerg	China:	Wood~~Wood~~	[2,10]

Formatted: Not Highlight

Commented [JHB137]: It would be possible to include the substrate?

Commented [JHB138R137]: We have modified the table. Please see updated one.

Formatted: Section start: Continuous, Width: 8.5", Height: 11", Numbering: Continuous

Formatted: Not Highlight

Formatted: Not Highlight

aquaticus	diam., 1-loculate, superficial to submerged, with a 500–800 µm long neck	× 9–10 µm, cylindrical	µm, filiform, slightly acute at each end, falcate, sigmoid, hyaline, 3–5-septate	ed	Yunnan		
O. arcuatisporum	313–324 × 252–340 µm, 1-loculate, superficial to immersed, with a long neck, up to 800 µm	276–307 × 15–20 µm, fusoid to narrowly cylindrical	170–239 × 4–7 µm, narrowly fusoid to cylindrical, falcate, hyaline to pale orange, 5–12-septate	Submerged	USA: Minnesota	*Typha* sp., herbaceous debris, grass Herb	[42]
O. bambusae	1 mm long, 2/3 mm diam., 1-loculate, immersed, with a 2–2.5 mm long neck	90–95 × 5.5–6.5 µm, cylindrical	75–80 × 1.5 µm, filiform, with both blunt ends, curved, hyaline, indistinctly septate	Terrestrial	Indonesia: Java	BambooBamboo	[50]
O. castillensis	525–650 µm diam., 1-loculate,	70–90 × 10–14 µm,	29–40 × 4–5.5 µm, narrowly	Terrestrial	Nicaragua	Bark and woodBark or wood	[8]

Formatted: Not Highlight

Formatted: Not Highlight

Formatted: Not Highlight

	superficial, with a 250–400 µm long neck	clavate	fusiform, slightly curved, hyaline to pale brown, 3-septate				
O. cecropiae	200–250 µm diam., 1-loculate, immersed, with a long neck, up to 2 mm	75–90 × 6.5–7.5 µm, cylindrical to subfusoid	60–70 × 2 µm, filiform, straight to slightly curved, hyaline, septate	Terrestrial	Venezuela	*Cecropia* sp.~~*Cecropia*~~	[63]
O. chiangdaoense	200–310 × 170–310 µm, 1-loculate, immersed, with a 93–273 µm long neck	85–125 × 11–17 µm, cylindrical	54–75.5 × 4–5.5 µm, filiform, falcate, fusoid at both ends, hyaline, 3-septate	Terrestrial	Thailand: Chiang Mai	Decaying leaves of *Dracaena loureiroi*~~*Dracaena loureiroi*~~	[9]
O. commune	150–350 × 260–400 µm, 1-loculate, immersed to superficial, with a 375–1660 µm long	64–118 × 4–12 µm, cylindrical	50–110 × 2 µm, filiform, arcuate or sigmoidal, hyaline, 3–7-septate	Submerged	Panama: Barro Colorado Island	Wood, herbaceous debris~~Wood~~	[2,42]

	neck						
O. dolichostomum	500 µm diam., 1-loculate, immersed, with a 1–5 mm long neck	100–130 × 8–12 µm, cylindrical	94–110 × 2–3 µm, filiform, falcate, sigmoid, hyaline, 3–7-septate	Submerged	USA: Florida	Dead wood Wood	[2,37]
O. filiforme	3–5 mm long, 150–180 µm diam., 1-loculate, immersed, erumpent to superficial, with a long neck	100–120 × 10–13 µm, clavate or fusoid	80–100 × 3–3.5 µm, filiform, hyaline to yellowish, multi-septate	Terrestrial	Indonesia: Java	Rotten leaf sheaths of *Amomum* sp.*Amomum*	[64]
O. fusiforme	360–500 × 330–450 µm, 1-loculate, immersed to erumpent, with a 250–800 µm long neck	70–112 × 6–12 µm, cylindrical	64–104 × 1.5–3 µm, filiform, fusoid, tapering at both ends, straight to falcate, 3–5-septate	Submerged	USA: Indiana	Decorticated woody debrisWood	[42]
O.	400–600 ×	130–160	100–128 ×	Submerged	China:	WoodWood	[46]

Formatted: Not Highlight

Formatted: Not Highlight

Formatted: Not Highlight

Formatted: Not Highlight

guttulatum	1200–1800 µm, 1-loculate, superficial to immersed, with a 500–1500 µm long neck	× 14–17 µm, broadly cylindrical	4–5 µm, cylindrical, falcate, pale yellow to hyaline, 3–5-septate	ed	Hong Kong		
O. hongkongense	500–640 × 700–800 µm, 1-loculate, superficial to immersed, with a more than 600 µm long neck	100–125 × 12–14 µm, elongated fusoid to broadly cylindrical	72–101 × 3.5–4.5 µm, cylindrical, falcate, tapered at both ends, hyaline, 3–5-septate	Submerged	China: Hong Kong	Wood~~Wood~~	[46]
O. indicus	400–650 µm diam., 1-loculate, immersed, with a long neck, up to 1.5 mm	65–90 × 8.5–11.5 µm, cylindrical to subfusoid	60–85 × 2.5–3.5 µm, filiform, tapering towards base, slightly curved, hyaline to subhyaline, 7–10-septate	Terrestrial	India: New Delhi	Dried twigs of *Ficus infectoria*~~*Ficus infectoria*~~	[38]

Formatted: Not Highlight

O. leptosporum	250–300 µm diam., 1-loculate, immersed or superficial, with a 1–2 mm long neck	70–95 × 5–6 µm, cylindrical	70–80 × 1–1.5 µm, filiform, apex rounded, base acute, hyaline to faintly tinted, straight to slightly curved or sigmoid, 3–7-septate	Submerged	UK: Exeter	Rotten stems of *Umbelliferae* sp.*Umbelliferae*	[4]
O. miyazakiense	Data unavailable	Data unavailable	Data unavailable	Terrestrial	Japan: Kyushu	Decaying litterDecaying litter	[65]
O. palmae	164–320 × 244–288 µm, 1-loculate, partly immersed, with a 180–376 µm long neck	76–96 × 10–14 µm, broadly cylindrical	79–90 × 1.2–2 µm, filiform, tapering at both ends, sigmoid, hyaline, 5-septate	Terrestrial	Philippines: Mt. Makiling	*Calamus ornatusCalamus ornatus*	[46]
O. parasiticum	600–800 µm diam.	100–140 × 9–11 µm	48–70 × 2.5–3.3 µm, 3–9-septate	Terrestrial (parasite)	China	Data unavailable	[9]
O. petrakii	600–750 ×	171–182	152–171 ×	Terrestri	India:	Dead stems of *Vitex*	[36]

Formatted: Highlight

Formatted: Not Highlight

	555–675 µm, 1-loculate, immersed, with a neck	× 12–15 µm, cylindrical	3–4 µm, filiform, apex rounded, base acute, slightly curved or sigmoid, hyaline, multi-septate	al	Karnataka	*negundoVitex negundo*	
O. rhizomorpha	500–900 × 500–750 µm, 1-loculate, superficial, with a 300–600 µm long neck	115–145 × 13–16 µm, clavate	39–49 × 3.5–4.5 µm, narrowly fusiform, slightly curved, hyaline to pale brown, 3-septate	Terrestrial	Kenya	Bark or woodBark or wood	[8]
O. sichuanense	230–350 × 300–7501500 µm, pseudostromatic, 1–5-loculate, semi-immersed to superficial, with long	90–115 × 5–6.5 µm, cylindrical	80–90 × 1–1.5 µm, filiform, slightly curved to sigmoidal, hyaline, aseptate	Submerged	China: Sichuan	Decaying branches of bamboo Bamboo	This study

Formatted: Not Highlight

Formatted: Not Highlight

	necks, up to 1 cm							
O. sorghi	300–400 µm diam., 1-loculate, immersed, with a 350–700 µm long neck	85–110 × 12–14 µm, cylindrical to clavate	75–95 × 3–4 µm, filiform, cylindrical, with rounded apex and slightly thinner rounded base, slightly curved, hyaline, 3–12-septate	Terrestrial	Central African Republic: M' Baiki	*Sorghum vulgareSorghum vulgare*	[4,34]
O. submersum	300–400 × 500–600 µm, 1-loculate, immersed, with a long neck	115–137 × 10–11 µm, cylindrical	87–109 × 3–4 µm, filiform, rounded at both ends, slightly curved or sigmoid, hyaline, multi-septate	Submerged	Thailand	Wood	[2]
O. tambopataense	Data unavailable	Data unavailable	Data unavailable	Terrestrial	Peru	Decaying leaf of palmPalm	[65]
O. tenuisporu	240–625 × 260–775 µm,	82–114 × 4–6	66–94 × 1–1.5 µm,	Submerged	Panama: Barro	Twig	[42]

Formatted: Not Highlight

m	~~Ascomata,~~ 1-loculate, superficial to partially immersed, with a long neck, up to 20 mm	µm, cylindrical to narrowly fusoid	filiform, more broadly rounded at one end than the other, curved to sigmoid, hyaline, 3-septate		Colorado Island		
O. venezuelense	730–890 × 745–868 µm, 1-loculate, partially immersed to superficial, with a 250–800 µm long neck	148–180 × 11–18 µm, cylindrical to narrowly fusoid	130–158 × 2–4 µm, filiform, falcate, more broadly rounded at one end than the other, straight to slightly curved, hyaline, (4)–5–(6)-septate	Submerged	Venezuela: Portuguesa	Wood, herbaceous debris~~Wood and herb~~	[42]
O. zeae	450–650 µm diam., 1-loculate, superficial or partially	55–65 × 7–8 µm, cylindrical to narrowly	39–50 × 2.3–2.5 µm, cylindrical to fusoid, with rounded	Terrestrial	Central African Republic: Boukoko	Dead *Zea mays*~~*Zea mays*~~	[4,33]

Formatted: Not Highlight

	immersed, with a 350–900 µm long neck	fusoid	ends, straight or slightly curved, hyaline, 3-septate				

Table 3. **Morphological comparisons of similar genera to *Ophioceras*.**

Generic name	Ascomata	Asci	Ascospores	References
Aquafiliformis (Magnaporthaceae)	Globose to subglobose, with long beak	Cylindrical to clavate, with an inconspicuous apical ring	Aseptate, filiform, hyaline	[2]
Ceratosphaeria (Ceratosphaeriaceae)	Globose to pyriform, with a long black or yellow neck	Cylindrical, with a conspicuous, non-amyloid, apical ring	Multi-septate, filiform, hyaline	[2]
Gaeumannomycella (Magnaporthaceae)	Subglobose to elliptical, with a lateral, central cylindrical neck	Cylindrical to elongated clavate, apical ring not observed	0–3-septate, narrowly fusiform, hyaline	[62]
Gaeumannomyces (Magnaporthaceae)	Subglobose to elliptical, with a cylindrical neck	Cylindrical to elongated clavate, with an apical refringent ring	Indistinctly septate, filiform, hyaline to pale brown	[61]
Kohlmeyeriopsis (Magnaporthaceae)	Ellipsoid, with a long cylindrical periphysate neck	Fusoid to cylindrical, with a large apical ring	Indistinctly septate, filamentous, tapering towards the base, hyaline	[1,66]
Muraeriata (Magnaporthaceae)	Lageniform to globose, with long beak	Cylindrical to ventricose, with a tall, narrow, apical ring	3-septate, narrowly fusiform, ends slightly curved, hyaline	[8]

Neogaeumannomyces (Magnaporthaceae)	Globose to subglobose, with a long, periphysate neck	Cylindrical, with an apical ring	2–3-septate, filiform to long fusiform, hyaline	[60]
Ophioceras (Ophioceraceae)	Subglobose to ampulliform, with a long cylindrical periphysate neck	Subcylindrical to narrowly fusoid or clavate asci with a small, refractive, non-amyloid apical ring	Aseptate to multi-septate, filiform, narrowly fusiform, hyaline pale brown or olivaceous	[2,8, this study]
Slopeiomyces (Magnaporthaceae)	Globose, with periphysate neck bearing hyphae	Clavate, with a non-amyloid apical ring	3–4-septate cylindrical to fusoid, tapering somewhat to base, hyaline	[1]

References

Formatted: Numbering: Continuous

1. Klaubauf S, Tharreau D, Fournier E, Groenewald JZ, Crous PW, de Vries RP, et al. Resolving the polyphyletic nature of *Pyricularia* (Pyriculariaceae). Stud Mycol. 2014;79:85–120. doi: 10.1016/j.simyco.2014.09.004
2. Luo ZL, Hyde KD, Liu JK, Maharachchikumbura SSN, Jeewon R, Bao DF, et al. Freshwater Sordariomycetes. Fungal Divers. 2019;99:451–660. doi: 10.1007/s13225-019-00438-1
3. Hyde KD, Norphanphoun C, Maharachchikumbura SSN, Bhat DJ, Jones EBG, Bundhun D, et al. Refined families of Sordariomycetes. Mycosphere. 2020;11:305–1059. doi: 10.1017/10.5943/mycosphere/11/1/7

4. Walker J. *Gaeumannomyces*, *Linocarpon*, *Ophiobolus* and several other genera of scolecospored Ascomycetes and *Phialophora* conidial states, with a note on hyphopodia. Mycotaxon. 1980;11:1–129.

5. Chen W, Shearer CA, Crane JL. Phylogeny of *Ophioceras* spp. based on morphological and molecular data. Mycologia. 1999;91:84–94. doi: 10.1080/00275514.1999.12060995

6. Wijayawardene NN, Hyde KD, Rajeshkumar KC, Hawksworth DL, Madrid H, Kirk PM, et al. Notes for genera: Ascomycota. Fungal. Divers. 2017;86:1–594. doi: 10.1007/s13225-017-0386-0

7. Saccardo PA. Sylloge Pyrenomycetum, Vol. II. Sylloge Fungorum. 1883;2:1–813.

8. Huhndorf SM, Greif M, Mugambi GK, Miller AN. Two new genera in the Magnaporthaceae, a new addition to *Ceratosphaeria* and two new species of *Lentomitella*. Mycologia. 2008;100:940–955. doi: 10.3852/08-037

9. Thongkantha S, Jeewon R, Vijaykrishna D, Lumyong S, McKenzie EHC, Hyde KD. Molecular phylogeny of Magnaporthaceae (Sordariomycetes) with a new species, *Ophioceras chiangdaoense* from *Dracaena loureiroi* in Thailand. Fungal Divers. 2009;34:157–173. doi: 10.1002/yea.1657

10. Hu DM, Cai L, Hyde KD. Three new ascomycetes from freshwater in China. Mycologia. 2012;104:1478–1489. doi: 10.3852/11-430

11. Index Fungorum. ~~2020~~ http://www.indexfungorum.org/Names/Names.asp. 2021 (~~accessed on~~ accessed on 8 April 2021~~17 June 2020~~).

12. Hu HL, Fournier J, Jeewon R, Bahkali AH, Hyde KD. Revisiting the taxonomy of *Daruvedia bacillata*. Mycotaxon. 2010;114:135–144. doi: 10.5248/114.135

13. NCBI: National Center for Biotechnology Information (GenBank)~~GenBank database~~. ~~2002~~

https://www.ncbi.nlm.nih.gov/nuccore/. 2020 http://www.indexfungorum.org/Names/Names.asp (Accessed accessed on 10 August 2020).

14. Hernández-Restrepo M, Bezerra JDP, Tan YP, Wiederhold N, Crous PW, Guarro J, et al. Re-evaluation of *Mycoleptodiscus* species and morphologically similar fungi. Persoonia. 2019;42:205–227. doi: 10.3767/persoonia.2019.42.08

15. Dai DQ, Phookamsak R, Wijayawardene NN, Li WJ, Bhat DJ, Xu JC, et al. Bambusicolous fungi. Fungal Divers. 2017;82:1–105. doi: 10.1007/s13225-016-0367-8

16. Jayasiri SC, Hyde KD, Ariyawansa HA, Bhat DJ, Buyck B, Cai L, et al. The Faces of Fungi database: fungal names linked with morphology, phylogeny and human impacts. Fungal Divers. 2015;74:3–18. doi: 10.1007/s13225-015-0351-8

17. White TJ, Bruns T, Lee SJWT, Taylor JW. Amplification and direct sequencing of fungal ribosomal RNA genes for phylogenetics. PCR—Protocols and Applications. 1990;18:315–322.

18. Vilgalys R, Hester M. Rapid genetic identification and mapping of enzymatically amplified ribosomal DNA from several *Cryptococcus* species. J Bacteriol. 1990;172:4238−4246. doi: 10.1128/jb.172.8.4238-4246.1990

19. Rehner SA. Primers for elongation factor 1-alpha (EF1-alpha). 2001. Available from: http://ocid.NACSE.ORG/research/deephyphae/EF1primer.pdf.

20. Jiang HB, Phookamsak R, Xu JC, Karunarathna SC, Mortimer PE, Hyde KD. Taxonomic and phylogenetic characterizations reveal three new species of *Mendogia* (Myriangiaceae, Myriangiales). Mycol Prog. 2020;19:41–51. doi: 10.1007/s11557-019-01540-z

21. Katoh K, Rozewicki J, Yamada KD. Mafft online service: multiple sequence alignment,

interactive sequence choice and visualization. Brief Bioinform. 2017. doi: 10.1093/bib/bbx108

22. Hall T. BioEdit version 5.0.6. North Carolina State University. 2001.

23. Stamatakis A. RAxML-VI-HPC: maximum likelihood-based phylogenetic analyses with thousands of taxa and mixed models. Bioinformatics. 2006;22:2688–2690. doi: 10.1093/bioinformatics/btl446

24. Silvestro D, Michalak I. raxmlGUI: a graphical front-end for RAxML. Org Divers Evol. 2011;12:335–337. doi: 10.1007/s13127-011-0056-0

25. Miller MA, Pfeiffer W, Schwartz T. Creating the CIPRES Science Gateway for inference of large phylogenetic trees. In: Gateway Computing Environments Workshop 2010 (GCE). 2010; pp.1–8. doi: 10.1109/GCE.2010.5676129

26. Nylander J. MrModeltest2 v. 2.3 (Program for selecting DNA substitution models using PAUP*). Evolutionary Biology Centre, Uppsala. 2008.

27. Ronquist F, Huelsenbeck JP. MrBayes 3: Bayesian phylogenetic inference under mixed models. Bioinformatics. 2003;19:1572–1574. doi: 10.1093/bioinformatics/btg180

28. Rannala B, Yang Z. Probability distribution of molecular evolutionary trees: a new method of phylogenetic inference. J Mol Evol. 1996;43:304–311. doi: 10.1007/BF02338839

29. Zhaxybayeva O, Gogarten JP. Bootstrap, Bayesian probability and maximum likelihood mapping: exploring new tools for comparative genome analyses. Genomics. 2002;3:1–15. doi: 10.1186/1471-2164-3-4

30. Perera RH, Maharachchikumbura SSN, Ariyawansa HA, Bahkali AH, Jones EBG, Al-Sadi AM, et al. Two new *Pseudohalonectria* species on beech cupules (*Fagus sylvatica*) and a new genus to accommodate *P. suthepensis*. Phytotaxa. 2016;278:115–131. doi:

10.11646/phytotaxa.278.2.2

31. Ellis JB, Everhart BM. The North American Pyrenomycetes. 1892;1–793.

32. Teng SJ. Notes on Sphaeriales from China. Sinensia. 1934;4:359–449.

33. Saccas AM. À propos de quelques champignons nouveaux parasites et saprophytes sur mais. Revue de Pathologe et d'Entomologie Agricole de France. 1951;30:161–196.

34. Saccas AM. Les champignons parasites des sorghos (Sorghum vulgare) et des penicillaires (Pennisetum typhoideum) en Afrique Equatoriale Française. Agronomie Tropicale. 1954;9:263–301.

35. Saccas AM. Étude de la Flore Cryptogamique des Caféirs en Afrique Centrale. 1981;1–522.

36. Tilak ST, Kale SB. Contribution to our knowledge of Ascomycetes of India - XX. Sydowia. 1969;23:17–23.

37. Conway KE, Barr ME. Classification of *Ophioceras dolichostomum*. Mycotaxon. 1977;5:376–380.

38. Lal SP. A new species of *Ophioceras* Sacc. from India. Kavaka. 1987;15:7–8.

39. Hyde KD. Tropical Australian freshwater fungi. IV. *Halosarpheia aquatica* sp. nov., *Garethjonesia lacunosispora* gen. et. sp. nov. and *Ophioceras dolichostomum* (Ascomycetes). Aust Syst Bot. 1992;5:407–414. doi: 10.1071/SB9920407

40. Hyde KD, Goh TK. Fungi on submerged wood in the Riviere St Marie-Louis, the Seychelles. S Afr J Bot. 1998;64:330–336. doi: 10.1016/S0254-6299(15)30920-0

41. Hyde KD, Goh TK. Fungi on submerged wood in Lake Barrine, north Queensland, Australia. Mycol Res. 1998;102:739–749. doi: 10.1017/S0953756297005868

42. Shearer CA, Crane JL, Chen W. Freshwater Ascomycetes: *Ophioceras* species. Mycologia.

1999;91:145–156. doi: 10.1080/00275514.1999.12061004

43. Matsushima T. Matsushima Mycological Memoirs 9. *Matsushima*. Mycol Mem. 2001;10:1–214.

44. Tsui CKM, Hyde KD, Fukushima K. Fungi on submerged wood in the Koito River, Japan. Mycoscience. 2001;44:55–59. doi: 10.1007/s10267-002-0083-y

45. Tsui CKM, Hyde KD, Hodgkiss IJ. Longitudinal and temporal distribution of freshwater ascomycetes and dematiaceous hyphomycetes on submerged wood in the Lam Tsuen River, Hong Kong. J N Am Benthol Soc. 2001;20:533–549.

46. Tsui CKM, Leung YM, Hyde KD, Hodgkiss IJ. Three new *Ophioceras* species (Ascomycetes) from the tropics. Mycoscience. 2001;42:321–326. doi: 10.1007/BF02461213

47. Cai L, Ji KF, Hyde KD. Variation between freshwater and terrestrial fungal communities on decaying bamboo culms. Antonie Van Leeuwenhoek. 2006;89:293–301. doi: 10.1007/s10482-005-9030-1

48. Abdel-Aziz FA. Freshwater fungi from the River Nile, Egypt. Mycosphere. 2016;7:741–756. doi: 10.5943/mycosphere/7/6/4

49. Farr DF, Rossman AY. Fungal Databases, U.S. National Fungus Collections, ARS, USDA. 2020. Available online: https://nt.ars-grin.gov/fungaldatabases/ (accessed on 1 June 2020).

50. Höhnel FV. Fragmente zur Mykologie: VI. Mitteilung (Nr. 182 bis 288). Sitzungsberichte der Kaiserlichen Akademie der Wissenschaften Math.-naturw. Klasse Abteilung I. 1909;118:275–452.

51. Tsui CKM, Hyde KD, Hodgkiss IJ. Biodiversity of fungi on submerged wood in Hong Kong streams. Aquat Microb Ecol. 2000;21:289–298. doi: 10.3354/ame021289

52. Ho WH, Hyde KD, Hodgkiss IJ. ~~(2001) ~~Fungal communities on submerged wood from streams in Brunei, Hong Kong, and Malaysia. Mycol Res. 2001;105:1492–150~~105:1492–1501~~. doi: 10.1017/S095375620100507X

53. Ho WH, Yanna, Hyde KD, Hodgkiss IJ. Seasonality and sequential occurrence of fungi on wood submerged in Tai Po Kau Forest Stream, Hong Kong. In: Fungal Succession (eds. K.D. Hyde and E.B.G. Jones). Fungal Divers. 2002;10:21–43.

54. Cai L, Zhang KQ, McKenzie EHC, Hyde KD. Freshwater fungi from bamboo and wood submerged in the Liput River in the Philippines. Fungal Divers. 2003;13:1–12. doi: 10.1016/S1567-1356(03)00107-7

55. Species Fungorum. ~~2020 ~~http://www.speciesfungorum.org/Names/Names.asp. 2021. (accessed on 8 April 2021).~~(accessed on 20 June 2020).~~

56. Hyde KD, Jeewon R, Chen YJ, Bhunjun CS, Calabon MS, Jiang HB, et al. The numbers of fungi: is the descriptive curve flattening? Fungal Divers. 2020. doi: 10.1007/s13225-020-00458-2

57. Maharachchikumbura SSN, Hyde KD, Jones EBG, McKenzie EHC, Bhat DJ, Dayarathne MC, et al. Fungal Divers. 2016;79:1–317. doi: 10.1007/s13225-016-0369-6

58. Hyde KD, Maharachchikumbura SSN, Hongsanan S, Samarakoon MC, Lücking R, Pem D, et al. The ranking of fungi: a tribute to David L. Hawksworth on his 70th birthday. Fungal Divers. 2017;84:1–23. doi: 10.1007/s13225-017-0383-3

59. Hongsanan S, Maharachchikumbura SSN, Hyde KD, Samarakoon MC, Jeewon R, Zhao Q, et al. An updated phylogeny of Sordariomycetes based on phylogenetic and molecular clock evidence. Fungal Divers. 2017;84:25–41. doi: 10.1007/s13225-017-0384-2

60. Liu JK, Hyde KD, Jones EBG, Ariyawansa HA, Bhat DJ, Boonmee S, et al. Fungal diversity notes 1–110: taxonomic and phylogenetic contributions to fungal species. Fungal Divers. 2015;72:1–197. doi: 10.1007/s13225-015-0324-y
61. Hernández-Restrepo M, Groenewald JZ, Elliott ML, Canning G, McMillan VE, Crous PW. Take-all or nothing. Stud Mycol. 2016;83:19–48. doi: 10.1016/j.simyco.2016.06.002
62. Crous PW, Schumacher RK, Akulov A, Thangavel R, Hernández-Restrepo M, Carnegie AJ, et al. New and interesting fungi. 2. Fungal Syst Evol. 2019;3:57–134. doi: 10.3114/fuse.2019.03.06
63. Müller E, Dennis RWG. Fungi venezuelani: VIII: Plectascales, Sphaeriales, Loculoascomycetes. Kew Bulletin. 1965;19:357–386. doi: 10.2307/4108161
64. Hennings P. Fungi monsunenses. Monsunia. 1900;1:137–174.
65. Matsushima T. *Matsushima*. Mycol Mem. 2003; 10. CD ROM [214 pp.+ incl. colour plates].
66. Kohlmeyer J, Volkmann-Kohlmeyer B, Eriksson OE. Fungi on *Juncus roemerianus*. 4. New marine ascomycetes. Mycologia. 1995;87:532–542. doi: 10.1080/00275514.1995.12026565

Supporting information

S1 Fig. RAxML tree generated from an analysis of the LSU matrix of taxa in Magnaporthales.

S2 Fig. RAxML tree generated from an analysis of the LSU-ITS matrix of taxa in Magnaporthales.

Dear Editor in PLOS ONE

Re: PONE-D-20-37408

Morphological and phylogenetic appraisal of *Ophioceras* (Ophioceraceae, Magnaporthales)

Please find the attached revised version of our manuscript "Morphological and phylogenetic appraisal of *Ophioceras* (Ophioceraceae, Magnaporthales)".

Those reviewer's comments were highly insightful and enable us to greatly improve the quality of our manuscript. Our point-by-point responses to each of the comments of the reviewers are mentioned in the following pages.

We prepare two revisions with track changes and without track changes. The manuscript is revised based on the reviewers and coauthors comments and checked the English grammar by native English speaker. We hope that revised manuscript is sufficient to make suitable for publication in PLOS ONE.

We shall look forward to hearing you at your earliest convenience.

Yours sincerely,

Response to Reviewer #1

Q: L33-36 Remove description from the abstract. The introduction already starts with it.

A: Thank you very much for your comment. We have removed the generic description from the abstract.

Q: L166-168 Remove them from "The USA". I suggest double checking the use of "the" across the text. There are some sentences that sound a bit odd, but nothing particularly problematic.

A: We have removed "the" from "The USA" in all entries. Please see the revised master text.

Q: Fig1. Try to convert to png. Jpg files lose resolution. Blue characters with green background might be difficult to see. I suggest inverting the colors (yellow-green). Tree is missing support values for some nodes, especially the node that separates Pseudohalonectriaceae and Ceratosphaeraceae+Ophioceraceae.

A: Tiff format of picture is the requirement of PLOS ONE. We have change blue characters to red. The missing support values are lower than 70% ML or 0.90 PP, thus, we delete these. But for values on the node that separates Pseudohalonectriaceae and Ceratosphaeraceae+Ophioceraceae, we will add in the updated phylogenetic tree.

Q: L249 Didymobotryum should be capitalized, as it refers to a genus. Also, I think it would be adequate to describe what Didymobotryum-like means.

A: We have changed the generic name to be capitalized and the explanation of Didymobotryum-like has already described in text.

Response to Reviewer #2

Q: 33-36 Are all this characters essential for the delimitation of the group? It would be nice If the authors could shorten this.

A: We have removed the generic description following the reviewer#1.

Q: L37 we collect submerged decaying culm of bamboo from Sichuan, ChinaAdd "a"

A: We have improved the sentence.

Q: L38-39 …and examined an *Ophioceras* species occurring on this specimen. Substrate?

A: We have improved the sentence.

Q: L39-40 We determined the taxon based on morphological characteristics and the combined LSU, RPB1 and ITS sequence analyses. Change "determined" to "delimited"

A: We have changed the word to "delimited" and improved the sentence.

Q: L44 …*Ceratosphaerella castillensis* and *C. rhizomorpha*… Are any of these species the type of genus?

A: We have added "(generic type)" after *Ceratosphaerella castillensis*, the type of *Ceratosphaerella*.

Q: L42-43 *Ceratosphaerella* resembles *Ophioceras*, but differs in the ascospore characters. This info could be ommited in the abstract.

A: We have removed this sentence from the abstract and modified the sentence.

Q: L52 The familial concept sounds unusual

A: We have changed to other words.

Q: L52-55 The familial concept is recognized as black, immersed to superficial, globose to subglobose, perithecial ascomata with long, periphysate necks, 8-spored, unitunicate, subcylindrical to narrowly fusoid asci with an apical ring, and filiform, hyaline to olivaceous, septate ascospores without sheaths [1,2,3]. what about the J- in the abstract?

A: a J-, apical ring is included in the description.

Q: L59 The initial accommodated *O. bacillatum*… change "initial" to "initially"

A: We have already changed it.

Q: L66 However, *O. bacillatum* is currently treated as *Daruvedia bacillata* (Cooke)… Is this relevant for the discussion of the results presented in this MS?

A: We have removed the sentence.

Q: L67-68 Eleven species of *Ophioceras* have molecular data in GenBank, and the LSU and ITS are mostly available for this genus what does it mean? LSU and ITS are the regions with greater number of sequences in the genus? Or that LSU and ITS are available to the majority of the species of the genus. I suggest rewriting the sentence.

A: We would like to inform that mostly LSU and ITS sequence data are available for eleven species of the genus. We have rewritten the sentence.

Q: L78-80 *Mycoleptodiscus* formed a clade separated from the other families in Magnaporthales. Currently, Mycoleptodiscus has been placed in Muyocopronaceae (Muyocopronales). Is this relevant for the discussion here? If it is, maybe a previous context about this genus should be presented.

A: We have removed the sentence.

Q: L81-86 whole paragraph I suggest not revealing all the results of the MS here. I would be better presenting it as the aims of the study. For ex: Test the phylogenetic relationship of *Ceratosphaerella* (or something like that); and something related to the study/identification of the sp. nov.

A: We have rewritten this paragraph.

Q: L92 Fungal fruiting bodies change to "Ascomata"

A: We have changed it to "Pseudostromata" as our fungus forms pseudostromata.

Q: L95-96 …by squash mount technique… change "squash" to "squashing"

A: We have changed it.

Q: L101 Measurements of pseudostromata, locules… number of structures measured per specimen?

A: We have added (n = 10-20) it means that we measure 10-20/structure.

Q: L111 The holotypic specimen of *Ophioceras sichuanense* is conserved… I would not mention the name of new sp. here.

A: We have deleted "of *Ophioceras sichuanense*".

Q: L138 …, our new isolate is closest with species of *Ophioceras*,… It is a result

A: We have moved it to the results part.

Q: L139 …with the unidentified fungal endophyte isolate 4583 (86.79% similarity)… this similarity seems very low.

A: It blast result of ITS has shown that our new species is closely related to the

unidentified fungal endophyte isolate 4583 but the similarity is very low as the reviewer's comment. However, the new taxon as also close to other taxa in Magnaporthales. The second closest taxon is Gaeumannomycella sp. isolate 15 (86.09% similarity), third closest taxon is *Ophioceras* sp. strain F0687 (86.05% similarity). Thus, ITS nucleotide blast result of our new isolate indicates our species should be in Magnaporthales. Although this similarity seems very low, it does not influence to reveal accurate position of our new isolate based on phylogenetic analyses of Magnaporthales. Preliminary analysis of ITS has also shown that our strains group in *Ophioceras*.

Q: L140 …clarify the phylogenetic placement of the new taxon… consider avoid "reveal" the new taxon here

A: We have changed "new taxon" to "new isolate" here.

Q: L147 if there are any conflicts between the tree topologies what about TEF?

A: Only 29 strains out of 66 strains have TEF seq data. Preliminary analysis by including TEF in our dataset does not showed significantly different tree topology comparing with the combined dataset of LSU-RPB1-ITS. Therefore, we exclude this gene locus from our analyses to avoid the undetermined characters or gaps.

Q: L147-149 The concatenated LSU-ITS and LSU-RPB1-ITS sequence datasets were further analyzed based on maximum-likelihood (ML) and Bayesian inference (BI) criteria for comparing the tree topologies. what do you mean with "comparing"?

A: We have rewritten the sentence.

Q: L155-156 GTR+I+G substitution model under the Akaike Information Criterion (AIC) was the best-fit evolutionary models for each locus result?

A: We have stated the results of evolution model after test here for further analyze BI criterion. This is a preliminary result for further phylogenetic analyses. Therefore, we did not state it in the results part.

Q: L147-149 The concatenated LSU-ITS and LSU-RPB1-ITS sequence datasets were further analyzed based on maximum-likelihood (ML) and Bayesian inference (BI) criteria for comparing the tree topologies. what about TEF and SSU?

A: The SSU and TEF1-α loci do not include in the present analyses due to the less available sequences of SSU and TEF1-α loci in our complied dataset and thus, these two loci were excluded to avoid missing data or gaps which may affect the tree topology.

Q: L171 Table 1 Detailed information of fungal taxa used in the present phylogenetic analyses incorporated with the GenBank accession numbers. delete "the present" and "incorporated with the GenBank accession numbers"

A: We have deleted "the present" and "incorporated with the GenBank accession numbers" from the sentence.

Q: Table1 where are SSU and TEF?

A: This table is included final sequence data that we used to infer the phylogenetic tree. Therefore, SSU and TEF are not included in this table.

Q: Table 1 *Ophioceras castillensis* (as *Ceratosphaeria castillensis*) the same for the other

A: "(as *Ceratosphaeria castillensis*)" and "(as *Ceratosphaerella rhizomorpha*)" have already been added after the new combined names.

Q: L183-188 Nomenclature Is this necessary?

A: It is the journal format.

Q: L204 …Magnaporthales including the new taxon… delete "including the new taxon"

A: We have deleted it.

Q: L204-205 *Myrmecridium schulzeri* (CBS 100.54) and *M. sorbicola* (CBS 143433) were selected as the outgroup taxa.method

A: We have moved this sentence from the results to method.

Q: L221-222 This phylogenetic result is in agreements with other previous studies by Huhndorf et al. [8], Perera et al. [30] and Luo et al. [2]. discussion

A: We have removed the sentence and discussed in the discussion part.

Q: L216-218 The tree topology from ML analysis showed similar results with the BI analysis and a comparing LSU-ITS, and LSU-RPB1-ITS phylograms were also similar in overall topologies (S2 Fig). after the sentence, add "Thus, we are going to use the LSU-RPB1-ITS topology for further discussion."

A: We have revised the sentences to be "Thus, we are going to use the LSU-RPB1-ITS topology for further discussion."

Q: L218-220 Phylogenetic analyses of the LSU-RPB1-ITS sequence matrix revealed that *Ophioceras sichuanense* (KUN-HKAS 107677 and KUMCC 20-0213) formed an independent lineage basal to *Ophioceras* in Ophioceraceae with high statistical support (100% ML, 1.00 PP; Fig 1). Delete *"Ophioceras sichuanense"* and add "the specimens investigated here"

A: We have revised the sentence to add "the investigated specimen (KUN-HKAS 107677) and its pure culture (KUMCC 20-0213) are grouped together".

Q: L224-225 However, these authors did not give an explanationon the phylogenetic results for *Ceratosphaerella*. Delete the sentence

A: We have deleted it.

Q: L219-220 …form an independent lineage basal to *Ophioceras* in Ophioceraceae with high statistic support (100% ML, 1.00 PP; Fig 1). After the sentence, add "In

light of these results and xxx, we propose a new species …"

A: We have added and modified this sentence.

Q: L221-222 *Ceratosphaerella* formed a separated clade far away from taxa in Magnaporthaceae and is grouped with other *Ophioceras* species in Ophioceraceae with moderate statistic support (81% ML, 0.91 PP; Fig 1). Distantly related?

A: We have modified this sentence.

Q: L221-222 *Ceratosphaerella* formed a separated clade far away from taxa in Magnaporthaceae and is grouped with other *Ophioceras* species in Ophioceraceae with moderate statistic support (81% ML, 0.91 PP; Fig 1). It should be better explained.

A: The phylogenetic analysis is rewritten with more explaination.

Q: Comment for result of "Molecular phylogeny part" I strongly suggest to present a more detailed description of the obtained topology. At least present the number and name of the recovered families.

A: Based on reviewer's suggestion, we have modified this part.

Q: Fig 1 legend change "Phylogram of RAxML analysis" to "Maximum likelihood tree"

A: We have changed it to "Maximum likelihood tree revealed by RAxML analysis"

Q: Fig 1 legend delete "The tree is rooted with *Myrmecridium schulzeri* (CBS 100.54) and *M. sorbicola* (CBS 143433)"

A: We have deleted it.

Q: L234 Ophioceraceae description What is new in this description? Is it necessary to present the full description of a known taxon. Does the features of the combined species change the circumscription of the group?

A: We have removed the familial description.

Q: L262 *Ophioceras* description same description as *Ophioceraceae*

A: We have emended the generic description.

Q: L299-300 Therefore, Ceratosphaerella is likely to to a synonym of *Ophioceras* instead of a genus in Ophioceraceae. you are not sure?

A: We have changed the word and modified the sentence.

Q: L300-303 Through ML and BI phylogenetic analyses based on a concatenated LSU-RPB1-ITS sequence matrix, *Ceratosphaerella castillensis* (SMH 1865) which was previously treated in Magnaporthaceae, is phylogenetically close to *C. rhizomorpha*

and clustered with *Ophioceras* in Ophioceraceae. I suggest to move the phylogenetic "comments" to the discussion.

A: These phylogenetic results are strongly support for our idea (*Ceratosphaerella* is a synonym of *Ophioceras*). Therefore, we think they should be remained in the notes of *Ophioceras*. In addition, we have also discussed phylogenetic result of *Ceratosphaerella* in the discussion.

Q: L300-303 Through ML and BI phylogenetic analyses based on a concatenated LSU-RPB1-ITS sequence matrix, *Ceratosphaerella castillensis* (SMH 1865) which was previously treated in Magnaporthaceae, is phylogenetically close to *C. rhizomorpha* and clustered with *Ophioceras* in Ophioceraceae. Change "with" to "within"

A: We have changed the word.

Q: L337-344 notes of *Ophioceras sichuanense* what about the phylogenetically related species? What are the differences and resemblances?

A: As requested from reviewer, we have modified the notes of *O. sichuanense*.

Q: L369-371 Hence, molecular data from the epitype of *C. castillensis* is required to confirm the conspecificity of the type specimen and Huhndorf's collection (SMH1865) as well as its phylogenetic position in *Ophioceras*. Are you questioning one of your main results?

A: In this sentence, we would like to discuss that even though *C. castillensis* is synonymized under *Ophioceras* based on current phylogenetic analyses in current study. But as mentioned above that the type specimen has not yet been sequenced. Therefore, this will bring an open question how to make sure that this species is real *C. castillensis* as it is not the extype strain. Therefore, the epitype of *C. castillensis* should be designated for confirm and support our result. However, we remove this sentence in introduction part and rewritten the discussion.

Q: L363-381 notes of *Ophioceras castillensis* I suggest to fucus the notes on the morphological characteristics and the comparisons related to it. The remaining info would be better suited in the discussion.

A: We have moved the paragraph to the introduction.

Q: L375-377 In this study, *Ceratosphaerella castillensis* is transferred to *Ophioceras* as *O. castillensis* based on concatenated LSU-RPB1-ITS analyses coupled with morphological similarity of the ascomata and ascomatal wall which is typical *Ophioceras*. Perhaps only with one LSU sequence

A: We have discussed the phylogenetic status of this species based on our final phylogenetic analyses.

Q: L380-381 Detailed morphologically comparison and the habitats of taxa in *Ophioceras* are described in a synopsis Table 2. Change "morphologically" to "morphological"

A: We have changed it.

Q: Fig 3 legend delete “≡ *Ceratosphaeria castillensis*”

A: We have deleted it.

Q: Fig 4 legend I am not sure if I understood this figure. It is a draw based in the original photographs? I checked the original article and the photographs are informative. I mean, what is the input of your figure? Same for fig 3.

A: We have provided the line drawing based on the type specimen here for a more convenient checking on morphological features of the species.

Q: L413 …and estimated in Hyde et al. [3]… “estimated” Is this the correct word?

A: We have changed “estimated” to “are accepted”.

Q: L410-417I believe that the information in this paragraph would be better in the introduction. It could reinforce the problematic and gap of knowledge that this manuscript aims to shed light.

A: We have moved the paragraph to the introduction.

Q: L420-425 Interesting information, but what is the relevance of this in relation to your results?

A: Although this information is not relevant to our results in this study. However, we provided this information as addition for further studies with fungal evolution approach and it will be useful for future taxonomic classification in a higher rank.

Q: L450-452 Preliminary phylogenetic analysis based on a single LSU gene and a concatenated LSU-ITS gene region have always shown that *O. castillensis* and *O. rhizomorpha* formed a stable clade within *Ophioceras* (S1 and S2 Figs). I cannot see the difference from the fig 1. In fig. 1 *O. castillensis* and *O. rhizomorpha* are grouped within *Ophioceras*.

A: We have revised the sentence to “In the present study, we synonymize *Ceratosphaerella* under *Ophioceras* based on molecular phylogeny coupled with similar ascomatal morphology. Phylogenetic analysis based on the LSU dataset (Fig. S1) and the concatenated LSU-ITS (Fig. S2) and the LSU-RPB1-ITS (Fig. 1) sequence datasets have always shown that *O. castillensis* (= *C. castillensis*) and *O. rhizomorpha* (≡ *C. rhizomorpha*) formed a stable clade within *Ophioceras*”.

Q: L452-453 Thus, we cannot exclude the possibility that Ceratosphaerella is a distinct genus in Ophioceraceae If it is true, your new species is not an Ophioceras, right? I don’t understand the point here, please clarify.

A: We have clarified the sentence.

Q: L465-466 It may be because of that there are only 14 species available in Species Fungorum [55] and the molecular data of…delete “of that there are only 14 species

available in Species Fungorum [55] and the”

A: We have deleted these words.

Q: L469-470 The new collections of *Pseudohalonectria* species are needed to obtain molecular data for solving the affinities within the genus. There is a lot of uncertainty like this in your discussion. I would not enter in this field (not directly relataded to your problem) with vague information.

A: We have removed the sentence. However, we have provided the discussion that some information may be not related to our aims because we would like to provide more information of the genera in our target family and related families in the same order, the taxonomic problem and future work for the genera in this group.

Q: comment for discussion I suggest to keep the focus of the discussion in Ophioceraceae

A: We have modified our discussion.

Q: comment for Table 2 It would be possible to include the substrate?

A: We have modified the table.
