# Supplementary material for: Morphological and phylogenetic appraisal of Ophioceras (Ophioceraceae, Magnaporthales)
Source: PLoS One. 2021 Aug 25;16(8):e0253853. doi: 10.1371/journal.pone.0253853 (PMC8386851; doi:10.1371/journal.pone.0253853)
Supplement: Attachment — Submitted filename: Response to Reviewers.docx [file pone.0253853.s004.docx]

Dear Editor in PLOS ONE

Re: PONE-D-20-37408

**Morphological and phylogenetic appraisal of *Ophioceras* (Ophioceraceae, Magnaporthales)**

Please find the attached revised version of our manuscript “Morphological and phylogenetic appraisal of *Ophioceras* (Ophioceraceae, Magnaporthales)”.

Those reviewer’s comments were highly insightful and enable us to greatly improve the quality of our manuscript. Our point-by-point responses to each of the comments of the reviewers are mentioned in the following pages.

We prepare two revisions with track changes and without track changes. The manuscript is revised based on the reviewers and coauthors comments and checked the English grammar by native English speaker. We hope that revised manuscript is sufficient to make suitable for publication in PLOS ONE.

We shall look forward to hearing you at your earliest convenience.

Yours sincerely,

**Response to Reviewer #1**

Q: L33-36 Remove description from the abstract. The introduction already starts with it.

A: Thank you very much for your comment. We have removed the generic description from the abstract.

Q: L166-168 Remove them from “The USA”. I suggest double checking the use of "the" across the text. There are some sentences that sound a bit odd, but nothing particularly problematic.

A: We have removed “the” from “The USA” in all entries. Please see the revised master text.

Q: Fig1. Try to convert to png. Jpg files lose resolution. Blue characters with green background might be difficult to see. I suggest inverting the colors (yellow-green). Tree is missing support values for some nodes, especially the node that separates Pseudohalonectriaceae and Ceratosphaeraceae+Ophioceraceae.

A: Tiff format of picture is the requirement of PLOS ONE. We have change blue characters to red. The missing support values are lower than 70% ML or 0.90 PP, thus, we delete these. But for values on the node that separates Pseudohalonectriaceae and Ceratosphaeraceae+Ophioceraceae, we will add in the updated phylogenetic tree.

Q: L249 Didymobotryum should be capitalized, as it refers to a genus. Also, I think it would be adequate to describe what Didymobotryum-like means.

A: We have changed the generic name to be capitalized and the explanation of Didymobotryum-like has already described in text.

**Response to Reviewer #2**

Q: 33-36 Are all this characters essential for the delimitation of the group? It would be nice If the authors could shorten this.

A: We have removed the generic description following the reviewer#1.

Q: L37 we collect submerged decaying culm of bamboo from Sichuan, China	Add “a”

A: We have improved the sentence.

Q: L38-39 …and examined an *Ophioceras* species occurring on this specimen.	Substrate?

A: We have improved the sentence.

Q: L39-40 We determined the taxon based on morphological characteristics and the combined LSU, RPB1 and ITS sequence analyses.	Change “determined” to “delimited”

A: We have changed the word to “delimited” and improved the sentence.

Q: L44 …*Ceratosphaerella* *castillensis* and *C.* *rhizomorpha…*	Are any of these species the type of genus?

A: We have added “(generic type)” after *Ceratosphaerella castillensis*, the type of *Ceratosphaerella*.

Q: L42-43 *Ceratosphaerella* resembles *Ophioceras*, but differs in the ascospore characters.	This info could be ommited in the abstract.

A: We have removed this sentence from the abstract and modified the sentence.

Q: L52 The familial concept	sounds unusual

A: We have changed to other words.

Q: L52-55 The familial concept is recognized as black, immersed to superficial, globose to subglobose, perithecial ascomata with long, periphysate necks, 8-spored, unitunicate, subcylindrical to narrowly fusoid asci with an apical ring, and filiform, hyaline to olivaceous, septate ascospores without sheaths [1,2,3].	what about the J- in the abstract?

A: a J-, apical ring is included in the description.

Q: L59 The initial accommodated *O. bacillatum*…	change “initial” to “initially”

A: We have already changed it.

Q: L66 However, *O. bacillatum* is currently treated as *Daruvedia bacillata* (Cooke)…	Is this relevant for the discussion of the results presented in this MS?

A: We have removed the sentence.

Q: L67-68 Eleven species of *Ophioceras* have molecular data in GenBank, and the LSU and ITS are mostly available for this genus	what does it mean? LSU and ITS are the regions with greater number of sequences in the genus? Or that LSU and ITS are available to the majority of the species of the genus. I suggest rewriting the sentence.

A: We would like to inform that mostly LSU and ITS sequence data are available for eleven species of the genus. We have rewritten the sentence.

Q: L78-80 *Mycoleptodiscus* formed a clade separated from the other families in Magnaporthales. Currently, Mycoleptodiscus has been placed in Muyocopronaceae (Muyocopronales).	Is this relevant for the discussion here? If it is, maybe a previous context about this genus should be presented.

A: We have removed the sentence.

Q: L81-86 whole paragraph	I suggest not revealing all the results of the MS here. I would be better presenting it as the aims of the study. For ex: Test the phylogenetic relationship of *Ceratosphaerella* (or something like that); and something related to the study/identification of the sp. nov.

A: We have rewritten this paragraph.

Q: L92 Fungal fruiting bodies	change to “Ascomata”

A: We have changed it to “Pseudostromata” as our fungus forms pseudostromata.

Q: L95-96 …by squash mount technique…	change “squash” to “squashing”

A: We have changed it.

Q: L101 Measurements of pseudostromata, locules…	number of structures measured per specimen?

A: We have added (n = 10-20) it means that we measure 10-20/structure.

Q: L111 The holotypic specimen of *Ophioceras sichuanense* is conserved…	I would not mention the name of new sp. here.

A: We have deleted “of *Ophioceras sichuanense*”.

Q: L138 …, our new isolate is closest with species of *Ophioceras*,…	It is a result

A: We have moved it to the results part.

Q: L139 …with the unidentified fungal endophyte isolate 4583 (86.79% similarity)…	this similarity seems very low.

A: It blast result of ITS has shown that our new species is closely related to the unidentified fungal endophyte isolate 4583 but the similarity is very low as the reviewer’s comment. However, the new taxon as also close to other taxa in Magnaporthales. The second closest taxon is Gaeumannomycella sp. isolate 15 (86.09% similarity), third closest taxon is *Ophioceras* sp. strain F0687 (86.05% similarity). Thus, ITS nucleotide blast result of our new isolate indicates our species should be in Magnaporthales. Although this similarity seems very low, it does not influence to reveal accurate position of our new isolate based on phylogenetic analyses of Magnaporthales. Preliminary analysis of ITS has also shown that our strains group in *Ophioceras*.

Q: L140 …clarify the phylogenetic placement of the new taxon…	consider avoid “reveal” the new taxon here

A: We have changed "new taxon” to “new isolate” here.

Q: L147 if there are any conflicts between the tree topologies	what about TEF?

A: Only 29 strains out of 66 strains have TEF seq data. Preliminary analysis by including TEF in our dataset does not showed significantly different tree topology comparing with the combined dataset of LSU-RPB1-ITS. Therefore, we exclude this gene locus from our analyses to avoid the undetermined characters or gaps.

Q: L147-149 The concatenated LSU-ITS and LSU-RPB1-ITS sequence datasets were further analyzed based on maximum-likelihood (ML) and Bayesian inference (BI) criteria for comparing the tree topologies.	what do you mean with “comparing”?

A: We have rewritten the sentence.

Q: L155-156 GTR+I+G substitution model under the Akaike Information Criterion (AIC) was the best-fit evolutionary models for each locus	result?

A: We have stated the results of evolution model after test here for further analyze BI criterion. This is a preliminary result for further phylogenetic analyses. Therefore, we did not state it in the results part.

Q: L147-149 The concatenated LSU-ITS and LSU-RPB1-ITS sequence datasets were further analyzed based on maximum-likelihood (ML) and Bayesian inference (BI) criteria for comparing the tree topologies.	what about TEF and SSU?

A: The SSU and TEF1-α loci do not include in the present analyses due to the less available sequences of SSU and TEF1-α loci in our complied dataset and thus, these two loci were excluded to avoid missing data or gaps which may affect the tree topology.

Q: L171 Table 1 Detailed information of fungal taxa used in the present phylogenetic analyses incorporated with the GenBank accession numbers.	delete “the present” and “incorporated with the GenBank accession numbers”

A: We have deleted “the present” and “incorporated with the GenBank accession numbers” from the sentence.

Q: Table1	where are SSU and TEF?

A: This table is included final sequence data that we used to infer the phylogenetic tree. Therefore, SSU and TEF are not included in this table.

Q: Table 1 *Ophioceras castillensis*	(as *Ceratosphaeria castillensis*) the same for the other

A: “(as *Ceratosphaeria castillensis*)” and “(as *Ceratosphaerella rhizomorpha*)” have already been added after the new combined names.

Q: L183-188 Nomenclature	Is this necessary?

A: It is the journal format.

Q: L204 …Magnaporthales including the new taxon…	delete “including the new taxon”

A: We have deleted it.

Q: L204-205 *Myrmecridium schulzeri* (CBS 100.54) and *M. sorbicola* (CBS 143433) were selected as the outgroup taxa.	method

A: We have moved this sentence from the results to method.

Q: L221-222 This phylogenetic result is in agreements with other previous studies by Huhndorf et al. [8], Perera et al. [30] and Luo et al. [2].	discussion

A: We have removed the sentence and discussed in the discussion part.

Q: L216-218 The tree topology from ML analysis showed similar results with the BI analysis and a comparing LSU-ITS, and LSU-RPB1-ITS phylograms were also similar in overall topologies (S2 Fig).	after the sentence, add “Thus, we are going to use the LSU-RPB1-ITS topology for further discussion.”

A: We have revised the sentences to be “Thus, we are going to use the LSU-RPB1-ITS topology for further discussion.”

Q: L218-220 Phylogenetic analyses of the LSU-RPB1-ITS sequence matrix revealed that *Ophioceras sichuanense* (KUN-HKAS 107677 and KUMCC 20-0213) formed an independent lineage basal to *Ophioceras* in Ophioceraceae with high statistical support (100% ML, 1.00 PP; Fig 1).	Delete *“Ophioceras sichuanense”* and add “the specimens investigated here”

A: We have revised the sentence to add “the investigated specimen (KUN-HKAS 107677) and its pure culture (KUMCC 20-0213) are grouped together”.

Q: L224-225 However, these authors did not give an explanationon the phylogenetic results for *Ceratosphaerella*.	Delete the sentence

A: We have deleted it.

Q: L219-220 …form an independent lineage basal to *Ophioceras* in Ophioceraceae with high statistic support (100% ML, 1.00 PP; Fig 1).	After the sentence, add “In light of these results and xxx, we propose a new species …”

A: We have added and modified this sentence.

Q: L221-222 *Ceratosphaerella* formed a separated clade far away from taxa in Magnaporthaceae and is grouped with other *Ophioceras* species in Ophioceraceae with moderate statistic support (81% ML, 0.91 PP; Fig 1).	Distantly related?

A: We have modified this sentence.

Q: L221-222 *Ceratosphaerella* formed a separated clade far away from taxa in Magnaporthaceae and is grouped with other *Ophioceras* species in Ophioceraceae with moderate statistic support (81% ML, 0.91 PP; Fig 1).	It should be better explained.

A: The phylogenetic analysis is rewritten with more explaination.

Q: Comment for result of “Molecular phylogeny part”	I strongly suggest to present a more detailed description of the obtained topology. At least present the number and name of the recovered families.

A: Based on reviewer’s suggestion, we have modified this part.

Q: Fig 1 legend	change “Phylogram of RAxML analysis” to “Maximum likelihood tree”

A: We have changed it to “Maximum likelihood tree revealed by RAxML analysis”

Q: Fig 1 legend	delete “The tree is rooted with *Myrmecridium schulzeri* (CBS 100.54) and *M. sorbicola* (CBS 143433)”

A: We have deleted it.

Q: L234 Ophioceraceae description	What is new in this description? Is it necessary to present the full description of a known taxon. Does the features of the combined species change the circumscription of the group?

A: We have removed the familial description.

Q: L262 *Ophioceras* description	same description as *Ophioceraceae*

A: We have emended the generic description.

Q: L299-300 Therefore, Ceratosphaerella is likely to to a synonym of *Ophioceras* instead of a genus in Ophioceraceae.	you are not sure?

A: We have changed the word and modified the sentence.

Q: L300-303 Through ML and BI phylogenetic analyses based on a concatenated LSU-RPB1-ITS sequence matrix, *Ceratosphaerella castillensis* (SMH 1865) which was previously treated in Magnaporthaceae, is phylogenetically close to *C.* *rhizomorpha* and clustered with *Ophioceras* in Ophioceraceae.	I suggest to move the phylogenetic “comments” to the discussion.

A: These phylogenetic results are strongly support for our idea (*Ceratosphaerella* is a synonym of *Ophioceras*). Therefore, we think they should be remained in the notes of *Ophioceras*. In addition, we have also discussed phylogenetic result of *Ceratosphaerella* in the discussion.

Q: L300-303 Through ML and BI phylogenetic analyses based on a concatenated LSU-RPB1-ITS sequence matrix, *Ceratosphaerella castillensis* (SMH 1865) which was previously treated in Magnaporthaceae, is phylogenetically close to *C.* *rhizomorpha* and clustered with *Ophioceras* in Ophioceraceae.	Change “with” to “within”

A: We have changed the word.

Q: L337-344 notes of *Ophioceras sichuanense*	what about the phylogenetically related species? What are the differences and resemblances?

A: As requested from reviewer, we have modified the notes of *O. sichuanense*.

Q: L369-371 Hence, molecular data from the epitype of *C. castillensis* is required to confirm the conspecificity of the type specimen and Huhndorf’s collection (SMH1865) as well as its phylogenetic position in *Ophioceras*.	Are you questioning one of your main results?

A: In this sentence, we would like to discuss that even though *C. castillensis* is synonymized under *Ophioceras* based on current phylogenetic analyses in current study. But as mentioned above that the type specimen has not yet been sequenced. Therefore, this will bring an open question how to make sure that this species is real *C. castillensis* as it is not the extype strain. Therefore, the epitype of *C. castillensis* should be designated for confirm and support our result. However, we remove this sentence in introduction part and rewritten the discussion.

Q: L363-381 notes of *Ophioceras castillensis*	I suggest to fucus the notes on the morphological characteristics and the comparisons related to it. The remaining info would be better suited in the discussion.

A: We have moved the paragraph to the introduction.

Q: L375-377 In this study, *Ceratosphaerella castillensis* is transferred to *Ophioceras* as *O.* *castillensis* based on concatenated LSU-RPB1-ITS analyses coupled with morphological similarity of the ascomata and ascomatal wall which is typical *Ophioceras*.	Perhaps only with one LSU sequence

A: We have discussed the phylogenetic status of this species based on our final phylogenetic analyses.

Q: L380-381 Detailed morphologically comparison and the habitats of taxa in *Ophioceras* are described in a synopsis Table 2.	Change “morphologically” to “morphological”

A: We have changed it.

Q: Fig 3 legend	delete “≡ *Ceratosphaeria castillensis*”

A: We have deleted it.

Q: Fig 4 legend	I am not sure if I understood this figure. It is a draw based in the original photographs? I checked the original article and the photographs are informative. I mean, what is the input of your figure? Same for fig 3.

A: We have provided the line drawing based on the type specimen here for a more convenient checking on morphological features of the species.

Q: L413 …and estimated in Hyde et al. [3]…	“estimated” Is this the correct word?

A: We have changed “estimated” to “are accepted”.

Q: L410-417	I believe that the information in this paragraph would be better in the introduction. It could reinforce the problematic and gap of knowledge that this manuscript aims to shed light.

A: We have moved the paragraph to the introduction.

Q: L420-425 Interesting information, but what is the relevance of this in relation to your results?

A: Although this information is not relevant to our results in this study. However, we provided this information as addition for further studies with fungal evolution approach and it will be useful for future taxonomic classification in a higher rank.

Q: L450-452 Preliminary phylogenetic analysis based on a single LSU gene and a concatenated LSU-ITS gene region have always shown that *O. castillensis* and *O.* *rhizomorpha* formed a stable clade within *Ophioceras* (S1 and S2 Figs).	I cannot see the difference from the fig 1. In fig. 1 *O. castillensis* and *O. rhizomorpha* are grouped within *Ophioceras*.

A: We have revised the sentence to “In the present study, we synonymize *Ceratosphaerella* under *Ophioceras* based on molecular phylogeny coupled with similar ascomatal morphology. Phylogenetic analysis based on the LSU dataset (Fig. S1) and the concatenated LSU-ITS (Fig. S2) and the LSU-RPB1-ITS (Fig. 1) sequence datasets have always shown that *O. castillensis* (= *C.* *castillensis*) and *O.* *rhizomorpha* (≡ *C.* *rhizomorpha*) formed a stable clade within *Ophioceras*”.

Q: L452-453 Thus, we cannot exclude the possibility that Ceratosphaerella is a distinct genus in Ophioceraceae	If it is true, your new species is not an Ophioceras, right? I don’t understand the point here, please clarify.

A: We have clarified the sentence.

Q: L465-466 It may be because of that there are only 14 species available in Species Fungorum [55] and the molecular data of…	delete “of that there are only 14 species available in Species Fungorum [55] and the”

A: We have deleted these words.

Q: L469-470 The new collections of *Pseudohalonectria* species are needed to obtain molecular data for solving the affinities within the genus.	There is a lot of uncertainty like this in your discussion. I would not enter in this field (not directly relataded to your problem) with vague information.

A: We have removed the sentence. However, we have provided the discussion that some information may be not related to our aims because we would like to provide more information of the genera in our target family and related families in the same order, the taxonomic problem and future work for the genera in this group.

Q: comment for discussion	I suggest to keep the focus of the discussion in Ophioceraceae

A: We have modified our discussion.

Q: comment for Table 2	It would be possible to include the substrate?

A: We have modified the table.
