# Supplementary material for: Morphological and phylogenetic appraisal of Ophioceras (Ophioceraceae, Magnaporthales)
Source: PLoS One. 2021 Aug 25;16(8):e0253853. doi: 10.1371/journal.pone.0253853 (PMC8386851; doi:10.1371/journal.pone.0253853)
Supplement: Attachment — Submitted filename: Response to Reviewers.docx [file pone.0253853.s006.docx]

Dear Editor and reviewers

Re: PONE-D-20-37408

**Morphological and phylogenetic appraisal of *Ophioceras* (Ophioceraceae, Magnaporthales)**

Please find the attached revised version of our manuscript “Morphological and phylogenetic appraisal of *Ophioceras* (Ophioceraceae, Magnaporthales)”.

Those reviewer’s comments were highly insightful and enable us to greatly improve the quality of our manuscript. Our point-by-point responses to each of the comments of the reviewers are mentioned in the following pages.

We prepare two revisions with track changes and without track changes. The manuscript is revised based on the reviewers and coauthors comments and checked the English grammar by native English speaker. We hope that revised manuscript is sufficient to make suitable for publication in PLOS ONE.

We shall look forward to hearing you at your earliest convenience.

Yours sincerely,

**Response to Reviewer**

L34: Remove “in this study”

**Response**: We have removed “in this study” from our manuscript.

L70-76: This information could be omitted “Huhndorf et al. [8] introduced Ceratosphaerella castillensis (≡ Ceratosphaeria castillensis) when the new genus Ceratosphaerella was introduced, and this species was designated as the generic type. Huhndorf et al. [8] examined the isotype of Ceratosphaeria castillensis compared with the morphology of their collection (SMH1865) and confirmed the conspecific status based only on morphological characteristics. Molecular data of C. castillensis was obtained from Huhndorf’s collection (SMH1865) but the type specimen of C. castillensis has not yet been sequenced”.

**Response**: We have removed this paragraph from our manuscript.

L 89-94: It has been suggested to not present all the results of the paper here. I suggest

not revealing all the results of the MS here. I would be better presenting it as the aims of the study. For ex: Test the phylogenetic relationship of *Ceratosphaerella* (or something like that); and something related to the study/identification of the sp. nov.

**Response**: We have changed the paragraph based on reviewer comment as “This study aims to introduce the novel *Ophioceras* taxon on a submerged bamboo branch in Sichuan Province, China and resolve the congeneric status of *Ceratosphaerella* and *Ophioceras* in Ophioceraceae based on a morpho-molecular approach”

L125: If neither TEF and SSU were used in tree, they should not appear here.

**Response**: We have removed TEF and SSU from the method part DNA extraction, amplification and sequencing.

L146: Is this the correct word?

**Response**: We have changed the word from “perform” to “generate” which mean produce (a set or sequence of items) by performing specified mathematical or logical operations on an initial set.

L157-160: There is no reason to mention SSU and TEF in this paper “It is notable that the SSU and TEF1-α loci do not include in the present analyses due to the less available sequences of SSU and TEF1-α loci in our complied dataset and thus, these two loci were excluded to avoid missing data or gaps which may affect the tree topology.”

**Response**: We have removed these sentences from our manuscript.

L180: Cannot access it again. Authors should present a "preliminary" link for the reviewers.

**Response**: We have resubmitted the alignment and tree to TreeBase and it can be accessed following the link as highlight in the manuscript.

L216: It is not closely.

**Response**: We have changed the word from “closely related” to “similar”

L253: Delete “revealed by RAxML analysis”

**Response**: We have already deleted it.

L266: Make sure that it is in accordance with the code as proposed here.

**Response**: We have checked the emendation and also asked help for Prof. Paul Kirk to check this emendation to confirm that it is in accordance with the code.

L406-411: I still believe that this information is should not be here. “The studies of fungal ranking based on molecular clock evidence showed that Ophioceraceae shares a common ancestor with Ceratosphaeriaceae at 65 MYA in Magnaporthales (130–190 MYA) and the subclass Diaporthomycetidae (247–250 MYA) was the youngest subclass in Sordariomycetes (130–250 MYA) [3,58]. However, the stem age and crown age of the familial ranking are not flexible depending on taxon sampling, gene selection in data matrix, models and parameters selection as well as the calibration points [58,59].”

**Response**: We have removed this paragraph from our manuscript.

L434: Wrong

**Response**: We have already changed the symbol of basionym.
